# Two structurally mobile regions control the conformation and function of metamorphic meiotic HORMAD proteins

**DOI:** 10.1038/s41467-026-72656-6

**Published:** 2026-05-19

**Authors:** Consuelo Barroso, Josh P. Prince, Punam Rattu, Mariana Sacerdoti, Daimona Koundé, Nuria Ferrandiz, Pablo Lopez-Jimenez, Syma Khalid, Enrique Martinez-Perez

**Affiliations:** 1 MRC Laboratory of Medical Sciences, London, UK; 2 Department of Biochemistry, Oxford University, Oxford, UK; 3 Imperial College Faculty of Medicine, London, UK; 4 Present Address: Centro de Investigacion del Cancer, CSIC, Salamanca, Spain

**Keywords:** Chromosomes, Protein folding

## Abstract

Metamorphic HORMA domain proteins (HORMADs) nucleate protein complex formation by refolding their mobile safety belt region to bind short closure motifs on interactors. Meiotic HORMADs (mHORMADs) bind proteinaceous axial elements to orchestrate complex chromosomal events that underpin fertility, including pairing and recombination between homologous chromosomes. However, the mechanisms supporting the diverse roles of mHORMADs remain unclear. Here, we show that mHORMADs have a second structurally mobile region, the β5-αC loop, which controls mHORMAD conformation and function. Molecular dynamics and in vivo approaches show that functional specialisation of *C. elegans* paralogs HTP-1 and HTP-2 depends on the interplay between their β5-αC loop and safety belt. The β5-αC loop can interact with the same HORMA core surface as the safety belt and mutations that hinder this interaction prevent HTP-1 binding to closure motifs in vitro and axis loading of HTP-1 and its paralog HTP-3 in vivo. Structural predictions of mHORMADs from yeast, plants, and mammals suggest that the β5-αC loop HORMA core interaction is a conserved feature of mHORMADs. Our study reveals that mHORMADs have expanded the bimodal folding landscape first identified in Mad2, paving the way to elucidate how non-canonical HORMAD conformations control meiotic chromosome function to ensure fertility.

## Abstract (web summary)

Metamorphic HORMA-domain proteins are essential components of meiotic chromosomes required for fertility. Here, the authors show that meiotic HORMADs contain a flexible loop region that is essential for controlling the conformational landscape of these proteins and their loading to chromosomes.

## Introduction

Accurate formation of haploid gametes from diploid germ cells during meiosis is essential for the fertility of sexually-reproducing organisms. Key to this process is the formation of inter-homologue crossover events during the prolonged prophase of the first meiotic division^1^. Crossovers, together with sister chromatid cohesion provided by the cohesin complex, provide the basis of chiasmata, physical attachments between homologous chromosomes that ensure their correct orientation on the first meiotic spindle^2^. Defects in the processes that promote chiasma formation result in sterility and the formation of aneuploid gametes, a cause of birth defects and miscarriages in humans.

The formation of chiasmata requires deliberate induction of DNA double strand breaks (DSBs) to initiate meiotic recombination, pairing and synapsis between homologous chromosomes, and repair of DSBs using the homologue as a repair template. These events in turn depend on the assembly of proteinaceous axial elements containing cohesin and a group of conserved proteins characterised by the presence of a HORMA domain (HORMADs) at the onset of meiotic prophase^3^. Meiotic HORMADs (mHORMADs) are essential for key chromosomal transactions of meiosis, including: DSB formation, pairing and synapsis of homologous chromosomes, repair of DSBs, and quality control mechanisms that monitor pairing and recombination intermediates to control meiotic progression^4^. Our mechanistic understanding of how mHORMADs promote this series of meiotic events is limited, but, similar to other HORMAD proteins, mHORMADs are thought to act as platforms for the assembly of protein complexes, thus the timing of their activity must be tightly regulated. Unicellular eukaryotes express a single mHORMAD, exemplified by Yeast Hop1^5^, but some multicellular organisms have undergone expansion of this protein family, including mammals (HORMAD1 and HORMAD2)^6–8^ and *C. elegans* (HTP-3, HTP-1, HTP-2, and HIM-3)^9–12^. In vivo mutant analysis provides evidence for functional specialisation between mHORMAD paralogs in these organisms^10,11,13–15^, but a mechanistic understanding of how these mHORMAD paralogs displaying highly identical amino acid sequences and protein structure have acquired functional specialisation is currently lacking.

The HORMA domain consists of a short and flexible N-terminus, a stable core of three α helices (α A-C) and a three-stranded β-sheet (β4-6), plus a flexible C-terminal domain called the safety belt. The two β sheets situated at the edges of the core (β6 and β5) serve as interaction surfaces for the flexible safety belt, giving HORMADs the ability to acquire at least two different folding configurations that determine the activity status of the protein^16^. This two-state behaviour is best understood for the spindle assembly checkpoint protein Mad2. Mad2 exists in an inactive and partner-free open conformation in which the safety belt interacts with β6 on the side of the core, and a partner-bound closed conformation where the safety belt moves across the HORMA core to interact with β5 and to wrap around a 6-10 amino acid closure motif (CM) on an interacting partner, thereby forming active Mad2-containing protein complexes^17,18^. This two-state behaviour is also present in Rev7^19^, a HORMAD component of different DNA repair complexes^20^. Similar to Mad2 and Rev7, mHORMADs also acquire a closed conformation bound to CMs^21^ and although Hop1 carries a chromatin-binding domain on its C-terminus^22,23^, binding a CM on axis-bound interactors is the main mechanism by which mHORMADs are recruited to axial elements^24,25^. However, in contrast to Mad2 and Rev7, a stable open conformation has not been identified in mHORMADs^16^, which adopt a partially-unfolded unbuckled conformation where the safety belt is disengaged from the core of the HORMA domain^25^. In addition, the flexible loop connecting β5 to αC is typically longer in mHORMADs than in Mad2^24^ (Supplementary Fig. 1) and its deletion has opposing conformational effects in Mad2 (stabilisation of an open conformation^17^) and yeast Hop1 (stabilisation of a closed conformation^25^). Therefore, although the two-state behaviour of Mad2 is thought of as a paradigm for HORMADs, the conformation dynamics of mHORMADs and how this correlates with their different functions remain poorly understood.

In contrast to Mad2, which consists exclusively of the HORMA domain, mHORMADs have additional N- and C-terminal domains flanking their HORMA domain that act as recruiting platforms for proteins controlling multiple aspects of meiotic chromosome metabolism^24,26–28^. Current models to explain mHORMADs’ mode of action suggest that the main role of the HORMA domain is targeting of the protein to axial elements by binding CMs on axis-bound proteins, while the extended C-terminus serves as a platform to recruit proteins that drive pairing, recombination, and checkpoint control. Then, removal of mHORMADs from the axis, a process that in most organisms is mediated by the ATPase remodeller protein Pch2 (Trip13)^29,30^, terminates activities driven by mHORMADs’ interactors. According to this model, the HORMA domain plays a relatively passive role once mHORMADs are bound to axial elements. However, recent reports show that the HORMA domain itself serves as an interaction surface to recruit recombination proteins to axial elements^31,32^. Thus, the HORMA domain of mHORMADs can support multiple functions simultaneously, suggesting that its conformational landscape may be more complex than previously thought. Moreover, differences in the behaviour of the HORMA domain of highly identical mHORMAD paralogs could potentially underpin their functional specialisation.

Here, we combine functional in vivo and in vitro studies with molecular dynamics modelling and protein folding prediction to investigate the functional specialisation of *C. elegans* HORMAD paralogs HTP-1 and HTP-2, which display 82% identity at the amino acid level. First, we demonstrate that major functional differences between these proteins are due to 7 amino acid changes in the safety belt region. Second, we identify the interplay between the safety belt and the extended β5-αC loop as key for controlling mHORMAD conformation and demonstrate that the β5-αC loop is essential for HTP-1 function in vivo. Third, structural predictions and in vivo analysis of *C. elegans* HTP-3 reveal that the β5-αC loop can interact with the HORMA core, forming a novel “loop engaged conformation” that is required for HTP-3 loading to chromosomes. Finally, structural prediction of mHORMADs in other organisms, including plants and mammals, suggest that the ability of the extended β5-αC loop to control the conformation of mHORMADs is a conserved feature of these proteins. We propose a model in which the position of the β5-αC loop determines different mHORMAD conformations that are essential for the successful execution of the meiotic programme and therefore for fertility.

## Results

### HTP-2 is unable to functionally replace HTP-1

HTP-1 and HTP-2 are 82% percent identical at the amino acid level and the crystal structures of their HORMA domains show that they appear nearly identical^24^. Despite this, previous analysis of single and double mutant worms carrying the *htp-1*(*gk174*) and *htp-2*(*tm2543*) alleles suggested that HTP-2 is unable to functionally replace HTP-1. While meiosis appears largely unaffected in *htp-2*(*tm2543*) mutants, *htp-1*(*gk174*) mutants display defects in homologue pairing, SC assembly, recombination, and checkpoint control of meiotic progression^10,11^. Analysis of *htp-1*(*gk174*) *htp-2*(*tm2543*) double mutants suggests that HTP-2 supports homology-independent SC assembly and together with HTP-1 controls the spatial release of cohesin during the first meiotic division^13,26^. In addition, immunostaining with antibodies recognising both HTP-1 and HTP-2 in wild-type (WT) controls, *htp-1(gk174)* mutants, and *htp-2(tm2543)* mutants indicated that HTP-1 is more abundant than HTP-2 on pachytene chromosomes^13^, suggesting that differences in chromosome loading may explain the inability of HTP-2 to functionally replace HTP-1. To clarify the contribution of HTP-1 and HTP-2 to crossover formation and whether this is determined by the relative amounts of these proteins on meiotic chromosomes, we used CRISPR to: First, create *htp-1* and *htp-2* alleles deleting the whole coding region of both genes (referred to as *htp-1∆* and *htp-2∆*, respectively). Second, create *htp-1::FLAG* and *htp-2::FLAG* alleles carrying a FLAG tag before the STOP codon. Third, substitute the *htp-1* gene (sequence between start codon and STOP codon) with the corresponding sequence from the *htp-2* gene, resulting in worms that carry two copies of *htp-2* (referred to as *twice htp-2*) in which the additional copy of *htp-2* is expressed under the endogenous *htp-1* promoter and 3’ UTR.

**Fig. 1:**
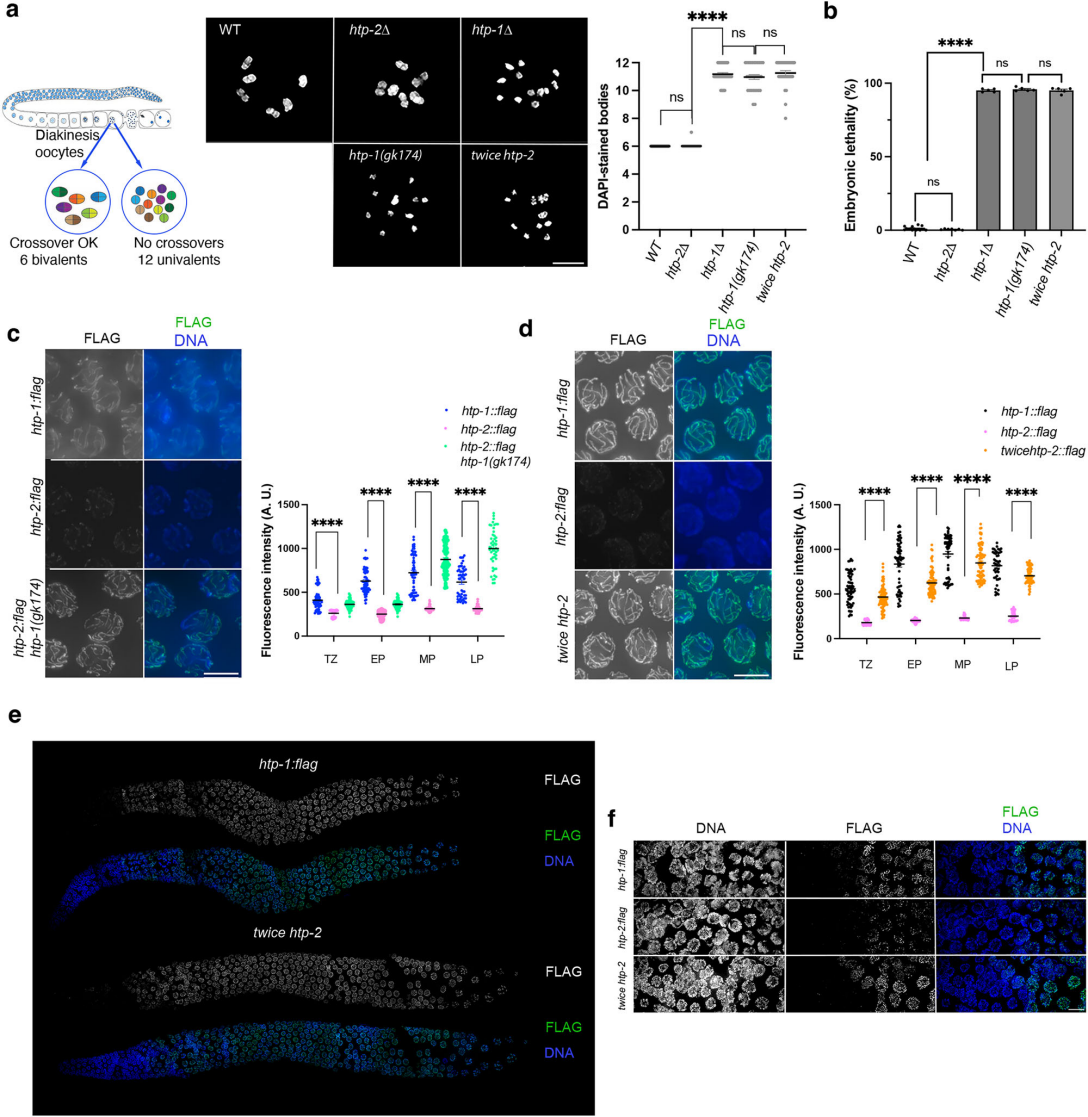
HTP-1 and HTP-2 are functionally distinct. **a** Cartoon of chromatin bodies visible in WT (6 bivalents) and a crossover deficient mutant (12 univalents) diakinesis oocytes. Projections of diakinesis oocytes from indicated genotypes stained with DAPI. 6 DAPI-stained bodies indicate normal crossover formation, while complete absence of crossover results in 12 DAPI-stained bodies. Graph shows quantification of number of DAPI-stained bodies per genotype (*n* = 30 (WT); *n* = 65 (*htp-2∆*); *n* = 35 (*htp-1∆*); *n* = 30 (*htp-1(gk174)*); *n* = 31 (*twice htp-2*), error bars indicate mean with 95% CI, p values were calculated using a two-tailed Mann-Whitney U test (**** indicates p < 0.0001). Note large crossover failure in *htp-1∆*, *htp-1(gk174)*, and *twice htp-2* mutants. **b** Quantification of embryonic lethality in strains of indicated genotypes. Number of worms and embryos analysed per genotype: WT = 15, 3421; *htp-2∆* = 7, 1842; *htp-1∆* = 5, 878; *htp-1(gk174)*= 5, 727; *twice htp-2* = 5, 1165, error bars indicate mean with 95% CI, *p* values were calculated using a two-tailed Mann-Whitney U test (**** indicates p < 0.0001). **c**, **d** Non-deconvolved projections of pachytene nuclei from indicated genotypes stained with anti-FLAG antibodies and DAPI. Graphs show intensity of anti-FLAG staining in nuclei of the indicated germline regions (transition zone (TZ), early pachytene (EP); mid pachytene (MP), and late pachytene (LP) and genotypes). Number of nuclei analyzed (c): *htp-1::FLAG*: 62 (TZ), 60 (EP), 61 (MP), 51 (LP); *htp-2::FLAG*: 60 (TZ), 60 (EP), 60 (MP), 50 (LP); *htp-2::FLAG htp-1(gk174)*: 60 (TZ), 60 (EP), 61 (MP), 49 (LP). Number of nuclei analyzed (d): *htp-1::FLAG*: 62 (TZ), 60 (EP), 60 (MP), 46 (LP); *htp-2::FLAG*: 62 (TZ), 64 (EP), 61 (MP), 45 (LP); *twice htp-2::FLAG*: 83 (TZ), 83 (EP), 82 (MP), 63 (LP). Error bars indicate mean with 95% CI, *p* values were calculated using a two-tailed Mann-Whitney U test (**** indicates *p* < 0.0001). **e** Projections of germlines stained with anti-FLAG antibodies and DAPI. **f** Deconvolved images of transition zone nuclei from indicated genotypes showing that FLAG associates with similar intensity and timing in *htp-1::FLAG* and *twice htp-2::FLAG* germlines. Scale bar = 5 µm in all panels. Source data are provided as a Source Data file.

The *C. elegans* genome contains six pairs of homologous chromosomes, which are observed as six DAPI-stained bodies in diakinesis oocytes of WT worms due to the formation of crossover events between all pairs of homologous chromosomes (Fig. 1a). In contrast, failure in crossover formation manifests in the presence of seven to twelve DAPI-stained bodies in diakinesis oocytes depending on the number of homologue pairs failing to undergo crossover formation. Oocytes of WT controls and *htp-2∆* mutants displayed six DAPI-stained bodies, confirming that HTP-2 is not required for crossover formation (Fig. 1a). In contrast, oocytes of *htp-1(gk174)*, *htp-1∆*, and *twice htp-2* mutants displayed an average of 10.9, 11.1, and 11.2 DAPI-stained bodies respectively, indicating a severe failure in crossover formation (Fig. 1a). We further corroborated this result by measuring embryonic lethality, which arises as a consequence of defective crossover formation between autosomes, and the incidence of male progeny, which provides a readout of crossover failure between the X chromosomes during meiosis. While WT controls and *htp-2∆* mutants displayed very low levels of embryonic lethality and male progeny, *htp-1(gk174)*, *htp-1∆*, and *twice htp-2* mutants displayed over 95% embryonic lethality and high levels of male progeny (Fig. 1b). Thus, HTP-2 alone, even when simultaneously expressed from its endogenous locus and the *htp-1* locus, is not capable of supporting crossover formation. We also confirmed that, as reported for the *htp-1*(*gk174*) *htp-2*(*tm2543*) double mutant^13^, *htp-1∆ htp-2∆* double mutants displayed reduced and delayed SC assembly compared with *htp-1∆* single mutants (Supplementary Fig. 2a), consistent with HTP-2 supporting homology-independent SC assembly.

Next, we used the FLAG-tagged versions of *htp-1* and *htp-2* in immunostaining experiments to determine the relative abundance of HTP-1 and HTP-2 and to compare the loading pattern of both proteins through meiotic prophase. Worms homozygous for *htp-1::FLAG* or *htp-2::FLAG* in otherwise WT genetic backgrounds displayed normal levels of crossovers and embryonic viability, confirming that FLAG tagging did not affect protein function (Supplementary Fig. 2b-c). Comparing anti-FLAG staining intensity in germlines of *htp-1::FLAG* and *htp-2::FLAG* homozygous worms demonstrated that during WT meiosis, the amount of HTP-1 associated with meiotic chromosomes was significantly higher than that of HTP-2 at all stages of meiotic prophase (Fig. 1c). To determine if the presence of HTP-1 affects the loading of HTP-2, we also measured the intensity of HTP-2::FLAG staining in germlines lacking HTP-1. Removal of HTP-1 caused a clear increase in the amount of HTP-2 associated with meiotic chromosomes, especially in nuclei at later stages of meiotic prophase (Fig. 1c). Although HTP-2::FLAG levels were also increased in transition zone and early pachytene nuclei of *htp-1* mutants, the intensity of the FLAG signal did not reach levels observed for that of HTP-1::FLAG (Fig. 1c). In contrast to the large increase in HTP-2 loading seen in *htp-1* mutants, removing HTP-2 only induced modest changes in HTP-1 levels (Supplementary Fig. 2d). Thus, HTP-2 loading is limited by HTP-1 but not vice versa.

Given that HTP-1 plays key roles in ensuring correct homologue pairing and synapsis during early meiotic prophase^10,11^, we wondered whether increasing the levels of chromosome-bound HTP-2 during early prophase would be sufficient to functionally replace HTP-1. Thus, we also imaged germlines from *twice htp-2* mutants in which we added a FLAG tag to the *htp-2* copy expressed from the *htp-1* locus, while the endogenous *htp-2* locus remained untagged. These germlines displayed a striking increase in the intensity of HTP-2::FLAG compared to WT *htp-2::FLAG* worms (Fig. 1d). In fact, the staining intensity and loading pattern of HTP-2::FLAG in germlines of *twice htp-2* were very similar to the intensity levels and loading pattern of HTP-1::FLAG observed in WT *htp-1::FLAG* germlines, including in transition zone nuclei (Fig. 1e-f). Crucially, however, *twice htp-2* mutants were not competent in crossover formation (Fig. 1a). Therefore, increasing the amount of HTP-2 that associates with chromosomes from the onset of meiosis is not sufficient to functionally replace HTP-1, demonstrating that HTP-1 and HTP-2 are functionally divergent. In addition, our observations also demonstrate that HTP-1 competes with HTP-2 for loading onto axial elements.

### Functional differences between HTP-1 and HTP-2 map to the C-terminal region of the HORMA domain

**Fig. 2:**
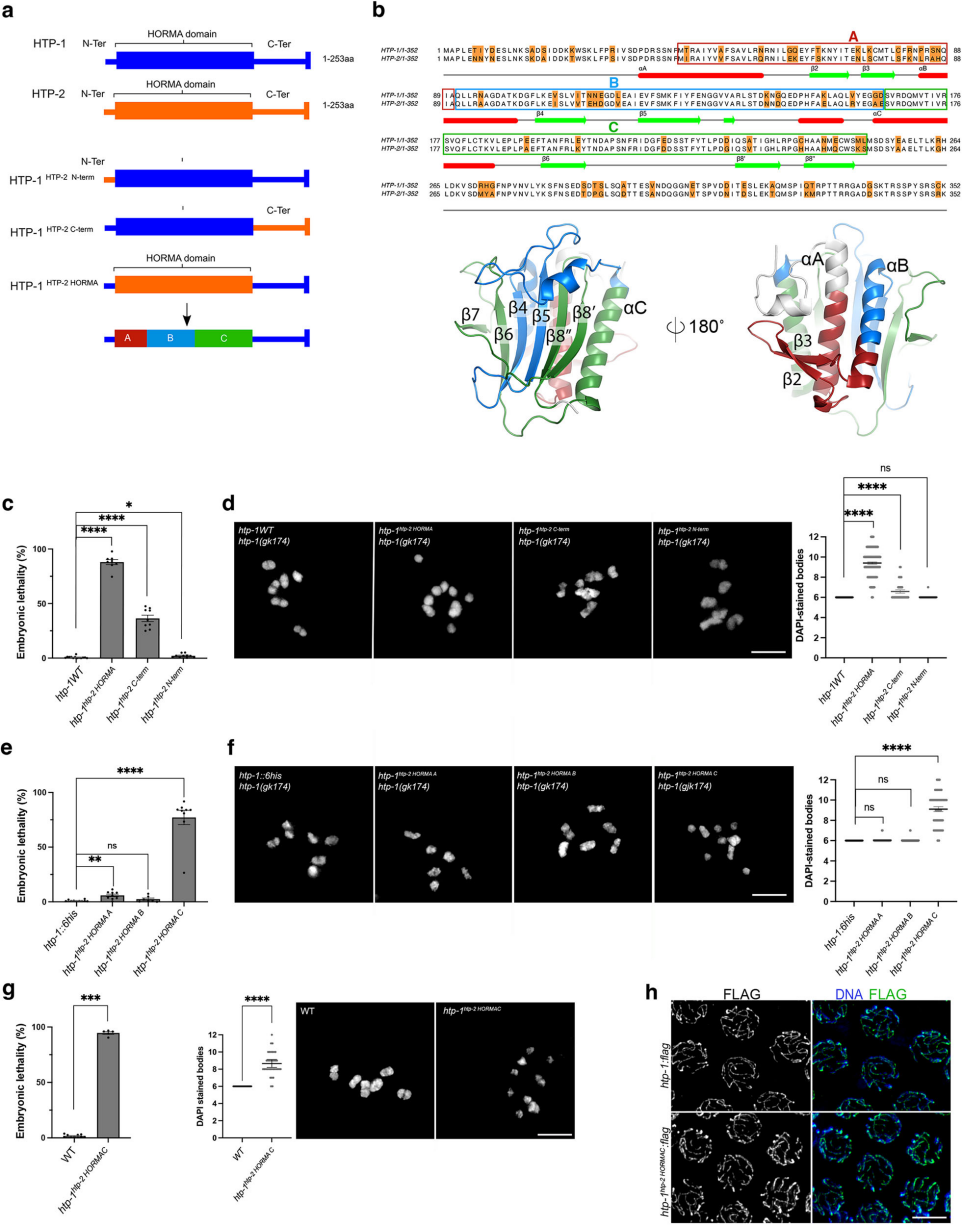
Mapping functional differences between HTP-1 and HTP-2. **a** Domains of HTP-1, HTP-2 and HTP-1-HTP-2 chimeras, including three subdivisions of the HORMA domain. **b** HTP-1 HTP-2 alignment indicating amino acid substitutions and HORMA subdivisions A (red), B (blue), and C (green). Bottom panel shows HTP-1 structure indicating HORMA subdomains by color. **c** Embryonic lethality. Number of worms and embryos analysed per genotype: *htp-1*^*WT*^ *htp-1(gk174)*= 13, 3685; *htp-1*^*htp-2 HORMA*^ *htp-1(gk174)*= 8, 1864; *htp-1*^*htp-2C-term*^ *htp-1(gk174)*= 9, 1888; *htp-1*^*htp-2 N-term*^ *htp-1(gk174)*= 10, 2835, error bars indicate mean with 95% CI, p values were calculated using a two-tailed Mann-Whitney U test (* indicates *p* = 0.0103; **** indicates *p* < 0.0001). **d** DAPI-stained bodies in diakinesis oocytes. Number of oocytes analysed *htp-1*^*WT*^ *htp-1(gk174)*= 39; *htp-1*^*htp-2 HORMA*^ *htp-1(gk174)*= 135; *htp-1*^*htp-2C-term*^ *htp-1(gk174)*= 34; *htp-1*^*htp-2 N-term*^ *htp-1(gk174)*= 60, error bars indicate mean with 95% CI, p values were calculated using a two-tailed Mann-Whitney U test (**** indicates *p* < 0.0001). **e** Embryonic lethality. Number of worms and embryos analysed per genotype: *htp-1*^*WT*^ *htp-1(gk174)*= 10, 2637; *htp-1*^*htp-2 HORMA A*^ *htp-1(gk174)*= 9, 2415; *htp-1*^*htp-2 HORMA B*^ *htp-1(gk174)*= 7, 1578^;^ *htp-1*^*htp-2 HORMAC*^ *htp-1(gk174)*= 9, 1590; error bars indicate mean with 95% CI, *p* values were calculated using a two-tailed Mann-Whitney U test (** indicates *p* = 0.0027; **** indicates *p* < 0.0001). **f** DAPI-stained bodies in diakinesis oocytes. Number of oocytes analysed *htp-1*^*WT*^ *htp-1(gk174)*= 36; *htp-1*^*htp-2 HORMA A*^ *htp-1(gk174)*= 42; *htp-1*^*htp-2 HORMA B*^ *htp-1(gk174)*= 59; *htp-1*^*htp-2 HORMAC*^ *htp-1(gk174)*= 46; error bars indicate mean with 95% CI, *p* values were calculated using a two-tailed Mann-Whitney U test (**** indicates *p* < 0.0001). **g** Embryonic lethality and DAPI-stained bodies in diakinesis oocytes of *htp-1*^*htp-2 HORMAC*^ (CRISPR) mutants. Numbers of worms and embryos analysed per genotype WT = 10, 2496; *htp-1*^*htp-2 HORMAC*^ = 5, 819. Number of oocytes analysed: WT = 30; *htp-1*^*htp-2 HORMAC*^ = 35. Error bars indicate mean with 95% CI, *p* values were calculated using a two-tailed Mann-Whitney U test (*** indicates *p* < 0.0007; **** indicates *p* < 0.0001). **h** Deconvolved projections of pachytene nuclei of stained with anti-FLAG antibodies and DAPI. Scale bar =5 µm in all panels. Source data are provided as a Source Data file.

Having found that HTP-2 is unable to provide HTP-1 functions needed for crossover formation, we reasoned that functional differences between these proteins must be conferred by differences in specific amino acids. In order to map the regions containing these amino acids, we decided to create a series of chimeric HTP-1 proteins in which different domains were replaced with the corresponding HTP-2 sequence. These chimeric proteins were expressed from a single-copy transgene containing the *htp-1* promoter and 3’ UTR and crossed into a *htp-1(gk174)* mutant background to determine whether the chimeric protein supports crossover formation. Meiotic HORMA-domain proteins, including HTP-1 and HTP-2, consist of three broad domains: A short N-terminal domain, the HORMA domain, and a C-terminal domain typically containing a CM (Fig. 2a). As the 60 amino acid differences (out of 352) between HTP-1 and HTP-2 are distributed relatively evenly between the different protein domains (Fig. 2b), we initially created chimeric proteins swapping the N-terminal (HTP-1^HTP-2 N-term^), HORMA (HTP-1^HTP-2 HORMA^), or C-terminal (HTP-1^HTP-2 C-term^) domains (Fig. 2a). Next, we assessed the functionality of these proteins by measuring embryonic lethality and number of DAPI-stained bodies in diakinesis oocytes of worms homozygous for the different transgenes and the *htp-1(gk174)* allele. A transgene expressing a WT version of HTP-1 was included as a positive control. *htp-1*^*htp-2 N-term*^ worms displayed levels of embryonic lethality and numbers of DAPI-stained bodies similar to WT controls (Fig. 2c-d), evidencing that the N-terminus of HTP-1/2 are functionally interchangeable. In contrast, worms expressing HTP-1^HTP-2 HORMA^ displayed 88% embryonic lethality and an average of 9.4 DAPI-stained bodies (Fig. 2c-d), demonstrating that the HORMA domain of HTP-2 does not support HTP-1 function. Finally, *htp-1*^*htp-2 C-term*^ worms displayed 36 % embryonic lethality and a slight increase in the number of DAPI-stained bodies (average of 6.6 versus 6 in controls) (Fig. 2c-d), suggesting the presence of mild meiotic defects. These experiments suggest that amino acid substitutions within the HORMA domain may be responsible for functional differences between HTP-1 and HTP-2.

Next, we divided the HORMA domain into 3 regions (HORMA A, B, and C) containing a similar number of amino acid differences (13, 13, and 10, respectively) (Fig. 2a-b) and created worms expressing the corresponding chimeric HTP-1/HTP-2 proteins from a single copy transgene in a *htp-1(gk174)* background as explained above. *htp-1*^*htp-2 HORMA A*^, *htp-1*^*htp-2 HORMA B*^, and *htp-1*^*WT*^ worms displayed low levels of embryonic lethality (Fig. 2e) and were competent in crossover formation as indicated by the number of DAPI-stained bodies in diakinesis oocytes (Fig. 2f). In contrast, *htp-1*^*htp-2 HORMA C*^ worms displayed high levels of embryonic lethality (Fig. 2e) and an average of 9.1 DAPI-stained bodies in diakinesis oocytes (Fig. 2f), demonstrating a severe crossover defect. Therefore, residues between E191 and L250, which expand the ß6 to ß8” regions of HTP-1’s HORMA domain (Fig. 2b) are critical for the ability of HTP-1 to ensure crossover formation. To corroborate this finding, we used CRISPR to introduce the 10 amino acid substitutions contained in the *htp-1*^*htp-2 HORMA C*^ transgene into the endogenous *htp-1* locus. *htp-1*^*htp-2 HORMA C*^ (CRISPR) mutants displayed 95% embryonic lethality and univalents in diakinesis oocytes (Fig. 2g), replicating our observations of worms expressing the *htp-1*^*htp-2 HORMA C*^ transgene in a *htp-1(gk174)* background. We also used CRISPR to introduce a FLAG tag before the STOP codon of HTP-1^HTP-2 HORMA C^ in order to monitor its loading to meiotic chromosomes. Anti-FLAG staining confirmed that HTP-1^HTP-2 HORMA C^::FLAG loaded to chromosomes throughout meiotic prophase (Fig. 2h). Therefore, the 10 amino acid substitutions present in HTP-1^HTP-2 HORMA C^ preclude HTP-1 from ensuring normal crossover formation despite substantial loading to meiotic chromosomes.

### HTP-1^HTP-2 HORMA C^ is deficient in promoting pairing, synapsis, and checkpoint regulation of meiotic progression

**Fig. 3:**
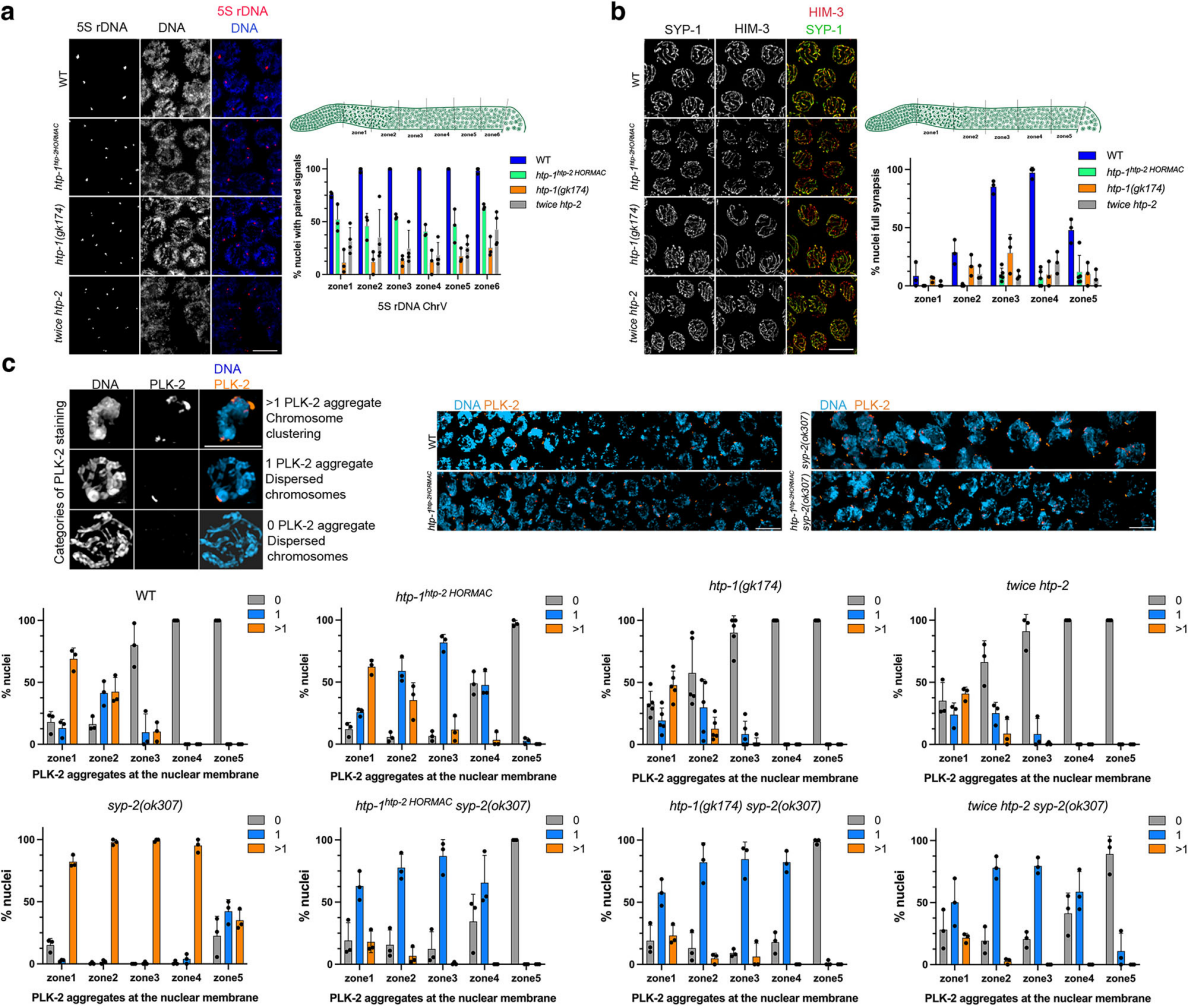
*htp-1*^*htp-2 HORMAC*^ mutants display multiple meiotic defects. **a** FISH (5S rDNA probe) images of pachytene nuclei and quantification of nuclei with paired signals in zones indicated in cartoon. Number of nuclei (3 to 4 germlines per genotype) analysed per zone= 124, 83, 72, 77, 64, 62 (WT); 107, 98, 90, 75, 81, 83 (*htp-1*^*htp-2 HORMAC*^); 100, 84, 70, 69, 74, 94 (*htp-1(gk174));* 99, 78, 63, 45, 49, 66 (*twice htp-2*). Error bar indicates mean with SD. **b** Projections of pachytene nuclei stained with anti-HIM-3 (axial element) and anti-SYP-1 (SC component) antibodies. Graph shows quantification of the percentage of nuclei displaying full synapsis (complete overlap of HIM-3 and SYP-1 signals) in zones indicated in top cartoon. Number of nuclei (3 to 5 germlines per genotype) analysed per zone = 88, 108, 97, 97, 74 (WT); 201, 248, 215, 194, 140 (*htp-1*^*htp-2 HORMAC*^); 119, 123, 117, 89, 79 (*htp-1(gk174));* 134, 146, 125, 104, 107 (*twice htp-2*). Error bars indicate mean with SD. Note SC defect in all *htp-1* mutants. **c** Left-hand side panels show single nuclei representing the three scored categories according to PLK-2 staining and chromatin morphology. Right-hand side panels show examples of projections of nuclei in transition zone and early pachytene regions of the germline stained with anti-PLK-2 antibodies and DAPI. Graphs show quantification of the % of nuclei with a given number of PLK-2 aggregates in five zones along the germline as indicated in the cartoon shown in b. Note that all *htp-1* mutants fail to accumulate nuclei with multiple PLK-2 aggregates, even in a *syp-2* mutant background. Number of nuclei (3 to 5 germlines per genotype) analysed per zone= 185, 173, 146, 129, 123 (WT); 131, 126, 109, 88, 104 (*htp-1*^*htp-2 HORMAC*^); 298, 265, 283, 236, 199 (*htp-1(gk174));* 162, 142, 145, 123, 123 (*twice htp-2*); 182, 180, 150, 128, 71 (*syp-2*); 158, 132, 123, 92, 69 (*htp-1*^*htp-2 HORMAC*^ *syp-2*); 127, 101, 99, 75, 71 (*htp-1(gk174) syp-2);* 162, 139, 138, 128, 128 (*twice htp-2 syp-2*). Error bars indicate mean with SD. Scale bar = 5 µm in all panels. Source data are provided as a Source Data file.

HTP-1 ensures crossover formation by participating in multiple meiotic events, including initial homologue recognition, SC assembly, checkpoint regulation of meiotic progression, and formation and repair of DSBs^10,11^. Thus, we next sought to clarify if the crossover defect observed in *htp-1*^*htp-2 HORMA C*^ mutants was due to defects in these events. WT and *htp-1(gk174)* mutants were included as positive and negative controls, respectively. We also included *twice htp-2* mutant worms in these experiments to clarify if increased levels of HTP-2 loading could improve any of the defects observed in *htp-1(gk174)* mutants, where only HTP-2 expressed from the *htp-2* locus is present. First, we assessed homologue pairing using fluorescence in situ hybridisation (FISH) to monitor the pairing of an interstitial region on chromosome V between the onset of meiotic prophase (transition zone nuclei) and late pachytene by dividing this section of the germline into six equal-size regions and counting the number of nuclei with paired signals in each zone (Fig. 3a). As expected, in WT control germlines pairing levels reached nearly a 100% for most of meiotic prophase (Fig. 3a). In contrast, *htp-1(gk174)*, *twice htp-2*, and *htp-1*^*htp-2 HORMA C*^ mutants displayed severely reduced levels of pairing compared to WT controls (Fig. 3a). Interestingly, pairing levels were slightly higher in *htp-1*^*htp-2 HORMA C*^ mutants than in *htp-1(gk174)* and *twice htp-2* mutants, suggesting that HTP-1^HTP-2 HORMA C^ retains a low level of pairing-promoting activity. Next, we used antibodies against axis protein HIM-3^9^ and SC component SYP-1^33^ to monitor the progression of synapsis between transition zone and late pachytene by dividing this region of the germline into five equal-size regions. Nuclei displaying HIM-3 stretches that did not colocalise with SYP-1 staining were scored as unsynapsed, while nuclei in which all HIM-3 stretches overlapped with SYP-1 signal were scored as synapsed. In WT controls the number of nuclei displaying full synapsis increased as nuclei progress into pachytene, reaching nearly 100 % by mid pachytene before decreasing as nuclei start the process of desynapsis in late pachytene (Fig. 3b). In contrast, the percentage of nuclei displaying full synapsis in *htp-1*^*htp-2 HORMA C*^ mutants never rose above 19% and overall was very similar to the pattern observed in *twice htp-2* mutants, evidencing a clear defect in SC assembly in both mutants (Fig. 3b). Moreover, simultaneous FISH and imaging of SYP-1 demonstrated that, similar to *htp-1* null mutants^10,11^, *htp-1*^*htp-2 HORMA C*^ mutants displayed extensive non homologous synapsis (Supplementary Fig. 3).

HTP-1 is an essential component of quality control mechanisms that orchestrate early meiotic progression by monitoring pairing and recombination intermediates to modulate the activity of the CHK-2 kinase, which induces DSB formation and chromosome movements required for pairing and synapsis^10,11,34–39^. One such mechanism monitors SC assembly to delay exit from early meiotic stages characterised by high levels of CHK-2 activity, which induce chromosome movements and DSB formation, until all homologue pairs achieve synapsis. A second HTP-1-dependent mechanism monitors the presence of crossover-fated recombination events between all homologue pairs to regulate exit from DSB-formation competent stages characterised by intermediate levels of CHK-2 activity^40,41^. These checkpoints arrest nuclei at the stage of meiotic prophase when the primary defect is identified: leptotene-zygotene (high CHK-2 activity) in the case of synapsis defects and early pachytene (intermediate CHK-2 activity) in the case of impaired recombination^35,42^. During leptotene and zygotene CHK-2 promotes chromosome movements, inducing chromosome clustering that can be readily observed by the crescent-shape appearance of chromatin in these nuclei (Fig. 3c). Targets of CHK-2 in this process include pairing center-binding (PCB) proteins that associate with the chromosomal end tethered to the nuclear envelope^35,43^. This induces recruitment of PLK-2 to PCB proteins, forming dynamic PLK-2 aggregates that modify nuclear envelope components to promote homologue pairing and synapsis^44,45^. Autosome-associated PLK-2 aggregates are dismantled once all homologue pairs achieve synapsis, but the PLK-2 aggregate associated with the paired X-chromosomes persists until crossover-fated events are formed on all homologue pairs^39^. Thus, mutants with synapsis defects accumulate nuclei with multiple PLK-2 aggregates, while mutants deficient in crossover formation but proficient in synapsis accumulate nuclei with a single PLK-2 aggregate. Crossover deficient mutants, regardless of their competence for SC assembly, also display CHK-2-dependent persistence of proteins that promote DSB formation, such as DSB-1 and DSB-2^40,41^.

Given the defect in SC assembly that we observed in *htp-1*^*htp-2 HORMA C*^ mutants (Fig. 3b), we reasoned that if HTP-1^HTP-2 HORMA C^ is competent in monitoring synapsis to regulate CHK-2 activity, then markers of chromosome movement should persist through the pachytene region. In WT controls, nuclei with multiple PLK-2 aggregates and chromosome clustering were most abundant in zone 1 (transition zone -leptotene and zygotene-) and 2 (early pachytene) and mostly lacking in zones 3 to 5 (Fig. 3c). Similarly, nuclei with multiple PLK-2 aggregates and chromosome clustering were largely restricted to zones 1 and 2 of *htp-1*^*htp-2 HORMA C*^, *htp-1(gk174)*, and *twice htp-2* mutants (Fig. 3c and Supplementary Fig. 4a), despite the synapsis defect observed in all three mutants (Fig. 3b). In contrast, *syp-2* mutants, which lack an essential component of the SC^46^ and were used as a positive control, displayed nearly a 100% of nuclei in zones 1 to 4 with multiple PLK-2 aggregates and chromosome clustering. Interestingly, *htp-1*^*htp-2 HORMA C*^, but not *htp-1(gk174)* or *twice htp-2*, mutants displayed an accumulation of nuclei with a single PLK-2 focus in zones 3 and 4 (Fig. 3c), suggesting persistence of intermediate levels of CHK-2 activity. As the temporal extension of CHK-2 activity in response to synapsis defects depends on the severity of the synapsis defect^35^, we wondered whether levels of SC assembly in *htp-1*^*htp-2 HORMA C*^, *htp-1(gk174)*, and *twice htp-2* mutants were sufficient to prevent CHK-2 activity extension. Thus, we crossed all three mutants into a *syp-2* mutant background to eliminate SC assembly. However, complete asynapsis failed to restore the accumulation of nuclei with multiple PLK-2 aggregates and chromosome clustering in *htp-1*^*htp-2 HORMA C*^, *htp-1(gk174)*, and *twice htp-2* mutants (Fig. 3c and Supplementary Fig. 4a). Instead, the three double mutants accumulated nuclei with 1 PLK-2 focus, suggesting that these mutants can only sustain intermediate levels of CHK-2 activity in the absence of synapsis (Fig. 3c).

We next investigated if *htp-1*^*htp-2 HORMA C*^ and *twice htp-2* mutants were competent in delaying exit from DSB-competent stages characterised by intermediate levels of CHK-2 activity when crossover formation is impaired. To achieve this, we monitored the presence of DSB-2, a marker of DSB competence whose removal from chromosomes is coupled to the successful formation of crossover-fated events on all chromosomes^40^. We included *htp-1(gk174)* and *syp-2* mutants as negative and positive controls for checkpoint activity, respectively. *htp-1*^*htp-2 HORMA C*^ mutants displayed a significant expansion of DSB-2-positive nuclei compared to WT controls, while *twice htp-2* mutants showed levels similar to *htp-1(gk174)* (Supplementary Fig. 4b). Thus, the recombination checkpoint, which delays exit from prophase stages with intermediate levels of CHK-2 activity when crossover formation is impaired, appears to be functional in *htp-1*^*htp-2 HORMA C*^ mutants but not in *twice htp-2* mutants. We investigated if the extended activity of DSB-2 seen in *htp-1*^*htp-2 HORMA C*^ mutants correlated with increased recombination intermediates by monitoring RAD-51 foci, which label intermediates downstream of DSB formation and upstream of crossover designation. Numbers of RAD-51 foci wereincreased in *htp-1*^*htp-2 HORMA C*^ mutants compared to *twice htp-2* and *htp-1(gk174)* mutants (Supplementary Fig. 4c), correlating with the extended region of DSB-2 activity observed in *htp-1*^*htp-2 HORMA C*^ mutants and suggesting that *htp-1*^*htp-2 HORMA C*^ mutants undergo increased DSB formation compared to *twice htp-2* and *htp-1(gk174)* mutants. Interestingly, numbers of RAD-51 foci in zones 2 and 3 were lower in *htp-1*^*htp-2 HORMA C*^ mutants than in wild-type controls, suggesting that HTP-1^htp-2 HORMA C^ is not fully competent for the DSB formation role proposed for HTP-1^10,11^.

The defects in pairing, synapsis, and checkpoint control of meiotic prophase observed in *htp-1*^*htp-2 HORMA C*^ mutants reveal that HTP-1^HTP-2 HORMA C^ is functionally closer to HTP-2 than HTP-1. Our findings also reveal that functional differences between HTP-1 and HTP-2 are not due to differences in loading to axial elements, as increasing levels of HTP-2 loading is not sufficient to functionally replace HTP-1’s roles in pairing, synapsis, and checkpoint control of meiotic prophase.

### The safety belt region of the HORMA domain controls functional differences between HTP-1 and HTP-2

**Fig. 4:**
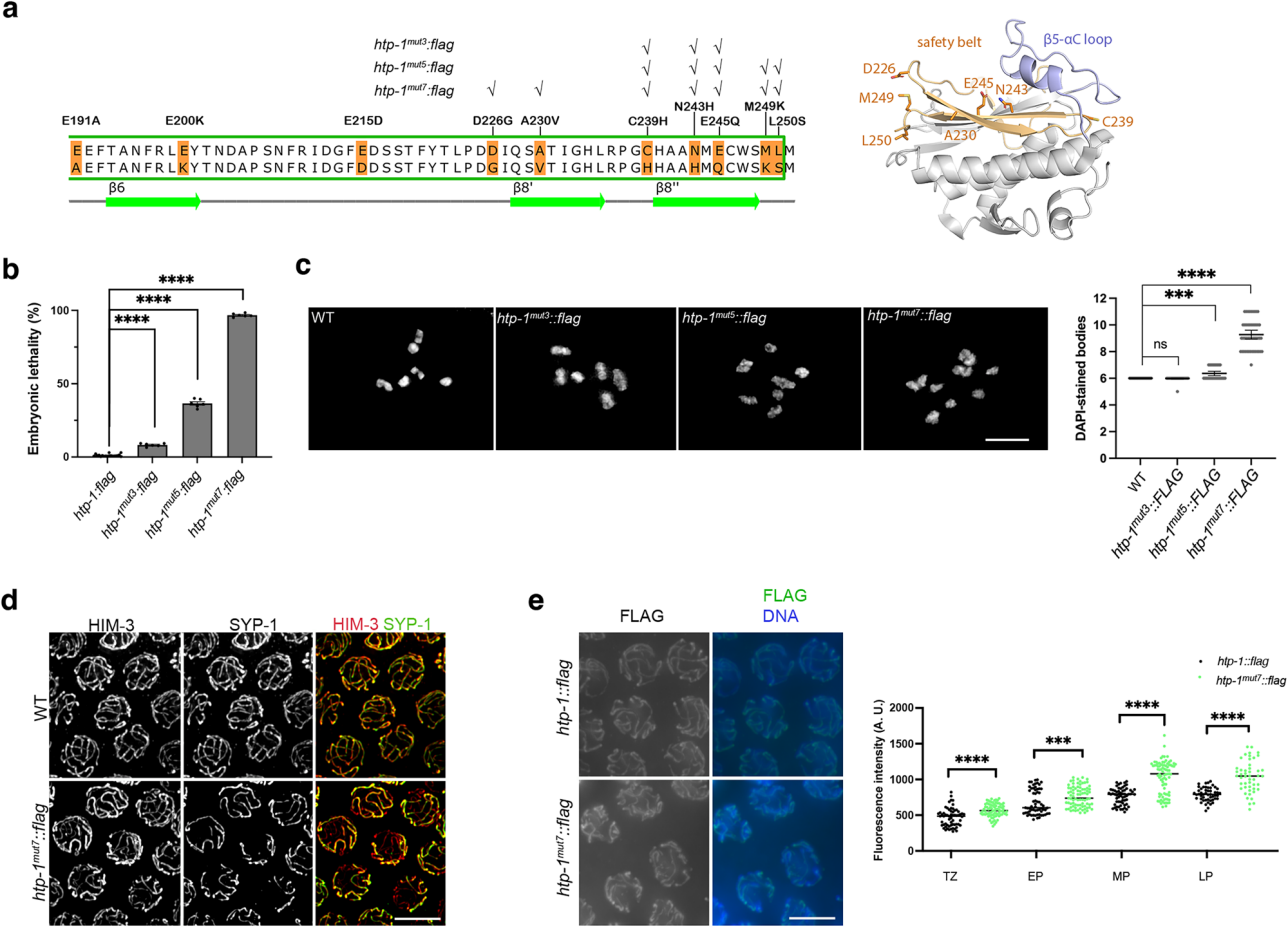
Amino acid substitutions in the safety belt region of HTP-1 induce meiotic defects. **a** Alignment of HTP-1 and HTP-2 indicating amino acid substitutions (highlighted in orange) and secondary structure features of both proteins. Tick symbols above alignment indicate amino acid substitutions included in each HTP-1 mutant protein. Right-hand panel shows the position of the amino acid substitutions in the crystal structure of HTP-1 HORMA domain in the closed conformation (PDB 4TZQ). **b** Quantification of embryonic lethality in strains of indicated genotypes. Number of worms and embryos analysed per genotype: *htp-1::FLAG* = 18, 5198; *htp-1*^*mut3*^*::FLAG* = 6, 1781; *htp-1*^*mu5*^*::FLAG* = 6, 1508; *htp-1*^*mut7*^*::FLAG* = 6, 871, error bars indicate mean with 95% CI, p values were calculated using a two-tailed Mann-Whitney U test (**** indicates *p* < 0.0001). **c** Projections of diakinesis oocytes of indicated genotypes stained with DAPI. 6 DAPI-stained bodies indicate normal crossover formation, while higher numbers indicate a crossover defect. Graph shows quantification of number of DAPI-stained bodies per genotype WT = *30*; *htp-1*^*mut3*^*::FLAG* = 50; *htp-1*^*mu5*^*::FLAG* = 36; *htp-1*^*mut7*^*::FLAG* = 48; error bars indicate mean with 95% CI, *p* values were calculated using a two-tailed Mann-Whitney U test (*** indicates *p* < 0.0003; **** indicates *p* < 0.0001). **d** Projections of pachytene nuclei stained with anti-HIM-3 (axial element) and anti-SYP-1 (SC component) antibodies. Lines displaying only HIM-3 staining (red signal) indicate the presence of unsynapsed regions, which are only detected in *htp-1*^*mut7*^*::FLAG* mutants. **e**) Non-deconvolved projections of pachytene nuclei from indicated genotypes stained with anti-FLAG antibodies and DAPI. Images were acquired and adjusted with the same settings for both genotypes. Graphs show intensity of anti-FLAG staining in nuclei of the indicated germline regions (transition zone (TZ), early pachytene (EP), mid pachytene (MP), and late pachytene (LP) and genotypes). Number of nuclei analyzed (three to four germlines per genotype)): *htp-1::FLAG*: 62 (TZ), 63 (EP), 62 (MP), 50 (LP); *htp-1*^*mut7*^*::FLAG*: 80 (TZ), 81 (EP), 85 (MP), 48 (LP). Error bars indicate mean with 95% CI, p values were calculated using a two-tailed Mann-Whitney U test (*** indicates *p* < 0.0009; **** indicates *p* < 0.0001). Note increased levels of HTP-1^mut7^::FLAG binding compared to HTP-1::FLAG. Scale bar =5 µm in all panels. Source data are provided as a Source Data file.

Having demonstrated that HTP-1^HTP-2 HORMA C^ is unable to provide key HTP-1 functions, we set out to further narrow down specific residues responsible for the functional differences between HTP-1 and HTP-2. The 10 amino acid substitutions present in HTP-1^HTP-2 HORMA C^ are contained in the interval between E191 and L250. A mutant strain carrying the E191A and E200K substitutions did not show obvious meiotic defects (Supplementary Fig. 5), and given the similarity between aspartic (D) and glutamic acid (E) we considered that the E215D substitution was unlikely to induce a strong effect. Thus, we focused on the 7 remaining mutations located between D226 and L250, corresponding to the safety belt of the HTP-1/2 HORMA domain, by creating mutants containing 3, 5, and 7 HTP-1-HTP-2 substitutions (Fig. 4a). We found a clear additive effect when comparing these mutants, with embryonic lethality and crossover defects increasing with the number of substitutions (Fig. 4b-c). Similar to *htp-1*^*htp-2 HORMA C*^ mutants, *htp-1*^*mut7*^ displayed 96% embryonic lethality and an average of 9.2 DAPI-stained bodies in diakinesis oocytes (Fig. 4b-c). Thus, we conclude that the crossover defect initially observed in *htp-1*^*htp-2 HORMA C*^ mutants is due to the seven amino acid substitutions present on the safety belt region. Moreover, similar to *htp-1*^*htp-2 HORMA C*^ mutants, *htp-1*^*mut7*^ mutants also display a strong defect in SC assembly, with pachytene nuclei displaying multiple unsynapsed regions (Fig. 4d). The meiotic defects observed in *htp-1*^*mut7*^ mutants were not due to impaired loading to axial elements, as levels of HTP-1^mut7^ protein were higher than those observed for WT HTP-1 (Fig. 4e). Thus, the safety belt region of HTP-1, which is involved in the binding of HTP-1 to the CMs in HIM-3 and HTP-3^24^, is key for HTP-1 functions, but not simply by promoting chromosomal loading. These findings identify the safety belt region as key to explain the functional differences between HTP-1 and HTP-2.

### Molecular dynamics simulations uncover different behaviour of HTP-1 and HTP-2 HORMA domains

**Fig. 5:**
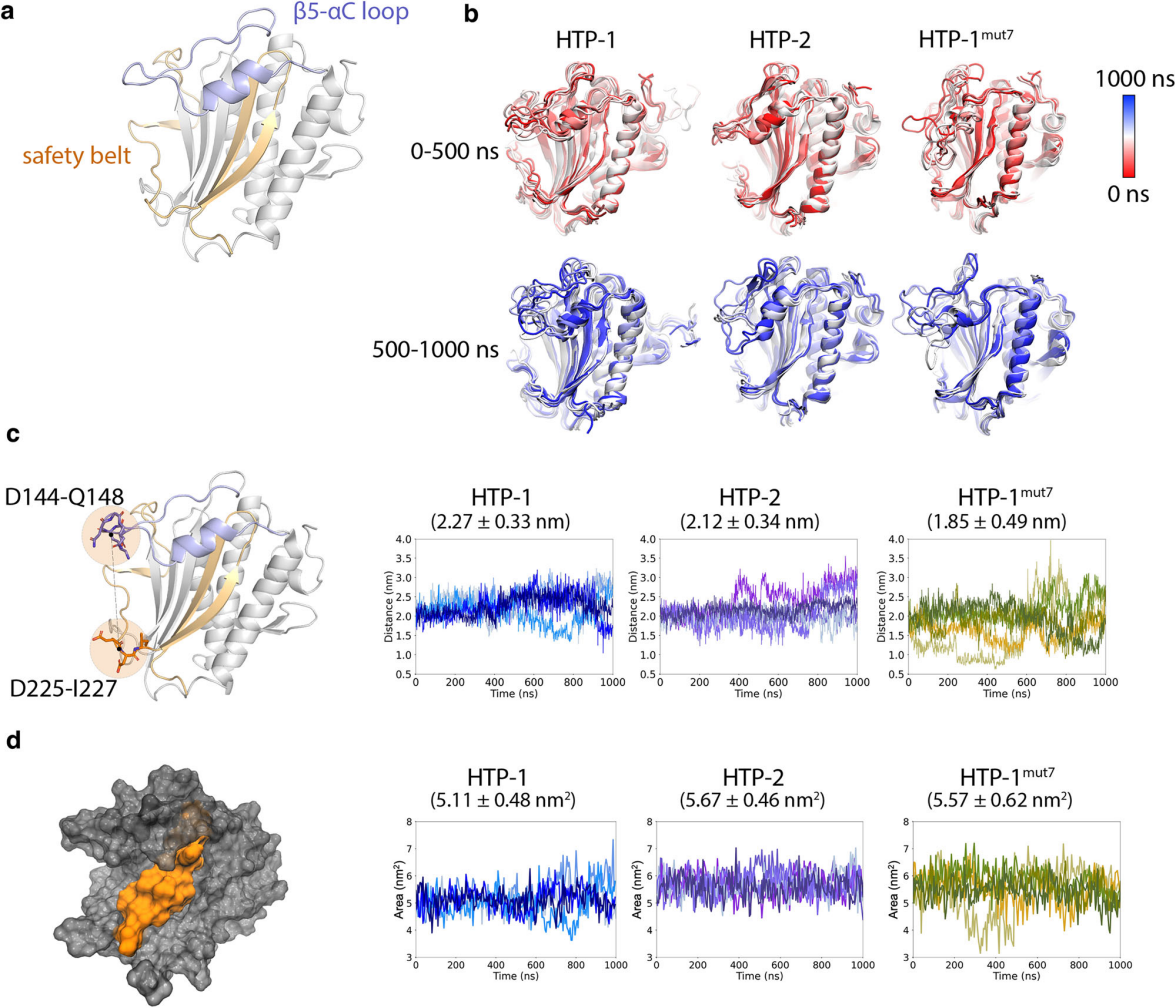
Molecular dynamics simulations of HTP-1, HTP-2, and HTP-1^mut7^ predict different dynamic behaviours. **a** Crystal structure of HTP-1’s HORMA domain in the closed conformation (PDB 4TZQ), highlighting the β5-αC loop (blue) and the safety belt (orange). **b** Protein dynamics of HTP-1, HTP-2, and HTP-1^mut7^ over the course of the 1000 ns simulation (replica 1). Frames every 100 ns were superimposed and aligned using the protein backbone, excluding residues within the β5-αC loop. **c** Distance measurements between the centre of mass of residues D144-Q148 in the β5-αC loop and D225-I227 in the safety belt (left, highlighted in orange) in 5 independent 1000 ns simulations. The mean distance across all simulations is reported above each plot. Note in HTP-1^mut7^ the decrease in distance between the β5-αC loop and the safety belt residues during the simulations, and the shorter mean distance compared to HTP-1 and HTP-2. **d** Solvent accessible surface area (SASA) measurements of the safety belt β8’ and β8” (left, orange) in 5 independent 1000 ns simulations. The mean SASA across all simulations is reported above each plot.

Given that HTP-1 and HTP-2 are highly similar in protein structure and interact with the same CMs on HTP-3 and HIM-3^24^, our findings above suggest that the dynamic regulation of HTP-1/2’s HORMA domain may underscore functional differences between these highly similar paralogs. We tested this hypothesis using equilibrated molecular dynamics (MD) simulations to compare the behaviour of HTP-1, HTP-2, and HTP-1^mut7^ HORMA domain (residues 17-253) over a 1000 ns interval. Simulations were based on the crystal structures of HTP-1’s (PDB 4TZQ) and HTP-2’s (PDB 4TZM) HORMA domain in the closed conformation^24^. Simulations were run without the bound CMs and repeated five independent times. Over the course of the simulations, despite lacking their bound CMs, all three proteins retained their closed unliganded conformation (empty closed conformation from now on) (Fig. 5a-b), in agreement with ColabFold predicted structures for HTP-1 and HTP-2 in the absence of external CMs (Supplementary Fig. 6a). To identify regions of greatest flexibility, we calculated the root mean square fluctuation (RMSF), which measures the average deviation of each residue from its starting position over the course of the simulation (Supplementary Fig. 6b). These revealed a high degree of flexibility in residues within the N-terminus, the β2-β3 hairpin, the β5-αC loop, and those in the β6-β8’ loop within the safety belt. The highest flexibility (excluding the very N and C-termini) was found in residues 140-150 corresponding to the β5-αC loop (Supplementary Fig. 6b), which is longer in mHORMADs than in Mad2 or Rev7 (Supplementary Fig. 1). This was also evident in the root mean square deviation (RMSD) measurements of residues 131-163, which suggested the β5-αC loop in HTP-1^mut7^ showed the greatest degree of movement from its starting position (Supplementary Fig. 6c). Differences in the range of movement of the β5-αC loop between HTP-1, HTP-2, and HTP-1^mut7^ can be appreciated by comparing superimposed time frames every 100 ns from the five independent simulations, which show that while the loop mostly remained upwards (away from the core of the of the HORMA domain) in HTP-1, in HTP-2 and particularly in HTP-1^mut7^ the loop moved downwards sitting on top of the safety belt formed by β8’ and β8” (Supplementary Fig. 7). To quantify the movement of the β5-αC loop, we measured the distance between the centre of mass of residues in the β5- αC loop (D144 to Q148) and a group of residues on the safety belt towards which the extended loop moves downwards (D225 to I227). The mean distance between these two residue groups (measured from five independent 1000 ns simulations) confirmed a slight reduction in HTP-2 (2.12 ± 0.34 nm) and a more pronounced reduction in HTP-1^mut7^ (1.85 ± 0.49 nm) compared to HTP-1 (2.27 ± 0.33 nm) (Fig. 5c). A closer look at the simulations suggested the increased downward motion identified in the β5-αC loops of HTP-1^mut7^ and HTP-2 were likely influenced, at least in part, by the residues at positions 239 and 243 (Supplementary Fig. 6d-e). In the simulations of both HTP-1^mut7^ and HTP-2, the distance between the α-carbon of H239 to that of G135 fluctuated substantially compared to α-carbon of C239 in HTP-1 and increased from a mean distance of 0.68 ± 0.07 nm in HTP-1 to 0.82 ± 0.13 nm and 0.82 ± 0.16 nm in HTP-1^mut7^ and HTP-2, respectively. This difference is likely a consequence of the amino acid substitution from cysteine in HTP-1 to histidine in HTP-1^mut7^ and HTP-2 (Supplementary Fig. 6d). The increased flexibility at the end of β5 in HTP-2 and HTP-1^mut7^, was accompanied by an additional movement within the small α-helix at the opposite end of the β5- αC loop. During all five simulations of HTP-1^mut7^ and three simulations of HTP2, this α-helix twisted to form an alternate position (Supplementary Fig. 6d), which was stabilised by interactions between H243, H152 and F153.

The findings above suggest that the β5-αC loop is a highly dynamic region of HTP-1/2 even when the HORMA domain is in a closed conformation. Moreover, in HTP-1^mut7^ and HTP-2 simulations, the downwards movement places the loop in close physical proximity to the safety belt region (Fig. 5b and Supplementary Fig. 7), suggesting that the loop could act to stabilise the closed conformation of the HORMA domain by interfering with the conformational changes that the safety belt is expected to undergo in the unlocking of closed-conformation HTP-1/2. A potential role of the β5-αC loop in stabilising the closed, CM-bound, conformation via interactions with residues on the safety belt has previously been proposed for HIM-3^24^. To determine the effects of this β5-αC loop movement on the safety belt region in HTP-1, HTP-1^mut7^, and HTP-2 we compared the solvent accessible surface area (SASA) of the safety belt (I227 to S248) in these simulations of the three proteins. This revealed a decreased SASA of HTP-1’s safety belt (5.11 ± 0.48 nm^2^) compared to that of HTP-2’s (5.67 ± 0.46 nm^2^) and HTP-1^mut7^ (5.57 ± 0.62 nm^2^) (Fig. 5e). Thus, in the closed conformation, the safety belt of HTP-2 is more solvent exposed, which could explain its decreased association to the axis in the presence of HTP-1. Although the SASA of HTP-1^mut7^ begins at a level comparable to that of HTP-2, the larger downward motion of the β5-αC loop over the course of the simulations results in a larger decrease in SASA in HTP-1^mut7^ compared to HTP-2. This presumably protects the protein from release and could explain the increase abundance seen associating with the chromosome axis in vivo.

In summary, the simulations of HTP-1, HTP-2, and HTP-1^mut7^ identify the β5-αC loop as a highly flexible region and suggest that the interplay between the β5-αC loop and other regions of HTP-1/2’s HORMA domain may be an important factor in determining the functions of these proteins.

### The β5-αC loop regulates HORMADs association with axial elements

**Fig. 6:**
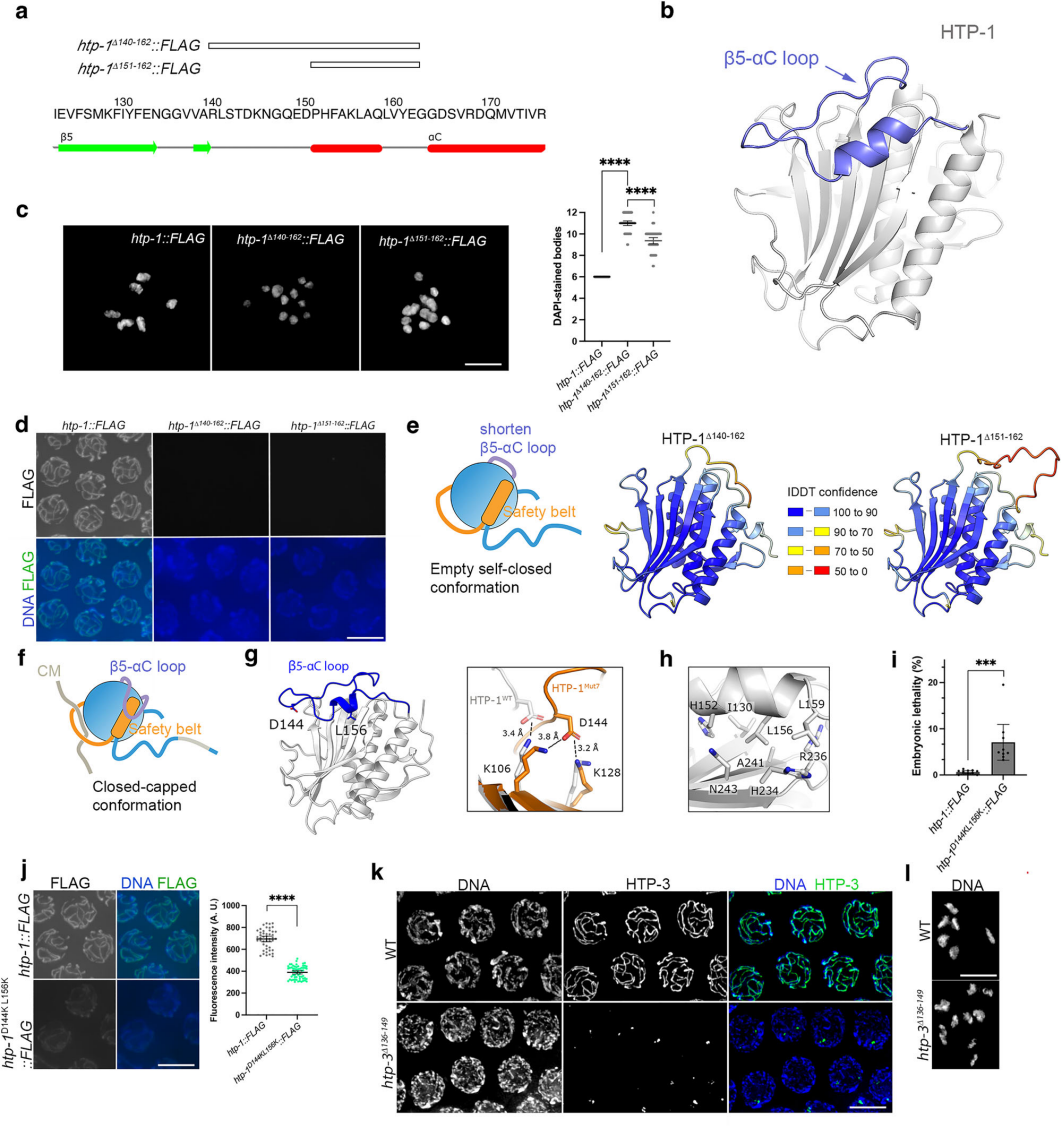
The β5-αC loop of HTP-1 and HTP-3 is required for axis loading and crossover formation. **a** Cartoon indicating amino acids deleted in two HTP-1 β5-αC loop mutant proteins. **b** Closed conformation model of HTP-1 indicating the position of the β5-αC loop. **c** Projections of diakinesis oocytes stained with DAPI and quantification of number of DAPI-stained bodies: *htp-1::FLAG* = 40; *htp-1*^*∆140-162*^*::FLAG* = 57; *htp-1*^*∆151-162*^*::FLAG* = 52, error bars indicate mean with 95% CI, *p* values were calculated using a two-tailed Mann-Whitney U test (**** indicates *p* < 0.0001). **d** Non-deconvolved projections of pachytene nuclei from indicated genotypes stained with anti-FLAG antibodies and DAPI. **e** Cartoon of mHORMAD in an empty closed conformation (circle: HORMA core, purple line (β5-αC loop), orange line (safety belt region) and ColabFold predictions of indicated HTP-1 loop deletion mutants, which display an empty self-closed conformation. **f** Cartoon of mHORMAD in a closed-capped conformation. **g** HTP-1 structure showing the position of D144 and L156 and panel showing interactions between D144 and K106 or K128 in the MD simulations of WT HTP-1 and HTP-1^mut7^. **h** HTP-1 structure displaying the position of L156 on the short helix at the C-terminus of β5-αC loop, sitting in a hydrophobic pocket. **i** Embryonic lethality in *htp-1::FLAG* controls and *htp-1*^*D144K L156K*^*::FLAG* mutants. Number of worms and embryos analysed per genotype: *htp-1::FLAG* = 6, 1373; *htp-1*^*D144K L156K*^*::FLAG* = 9, 1840, error bars indicate mean with 95% CI, *p* values were calculated using a two-tailed Mann-Whitney U (*** indicates *p* < 0.0004). **j** Non-deconvolved projections of pachytene nuclei from indicated genotypes stained with anti-FLAG antibodies and DAPI. Graph shows intensity of anti-FLAG staining in pachytene nuclei. 47 nuclei were analysed in *htp-1::FLAG* and 60 in *htp-1*^*D144K L156K*^*::FLAG*, error bars indicate mean with 95% CI, *p* values were calculated using a two-tailed Mann-Whitney U test (**** indicates *p* < 0.0001). **k** Projections of pachytene nuclei of indicated genotype stained with anti-HTP-3 pS285 antibodies and DAPI. Note that HTP-3^*∆136-149*^ fails to form axial elements. **l** Projections of diakinesis oocytes of indicated genotypes stained with DAPI. Scale bar = 5 µm in all panels. Source data are provided as a Source Data file.

Our MD simulations suggest that the β5-αC loop may play an important role in controlling the conformation of HTP-1/2’s HORMA domain. We started investigating the functionality of this domain by creating CRISPR mutants carrying either a whole (HTP-1^∆140-162^::FLAG) or partial (HTP-1^∆151-162^::FLAG) deletion of the β5-αC loop region (Fig. 6a-b). Crossover formation was impaired in both *htp-1*^*∆140-162*^ and *htp-1*^*∆151-162*^ mutants, as evidenced by the presence of elevated numbers of DAPI-stained bodies in diakinesis oocytes (average of 11 and 9.4 respectively, compared to 6 in controls) (Fig. 6c). Interestingly, differences in the number of DAPI-stained bodies between *htp-1*^*∆140-162*^ and *htp-1*^*∆151-162*^ mutants are statistically significant (Fig. 6c), evidencing that HTP-1^∆151-162^ retains some crossover-promoting activity. Both *htp-1*^*∆140-162*^ and *htp-1*^*∆151-162*^ mutants showed severe synapsis defects, with almost a 100% of pachytene nuclei containing unsynapsed regions (Supplementary Fig. 8a). Given this, we next tested whether HTP-1^∆140-162^ and HTP-1^∆151-162^ were capable of providing checkpoint control of SC assembly by delaying meiotic prophase in the presence of unsynapsed chromosomes. Staining with anti-PLK-2 antibodies revealed that *htp-1*^*∆140-162*^ mutants failed to accumulate nuclei with PLK-2 aggregates, while *htp-1*^*∆151-162*^ mutants displayed accumulation of nuclei with PLK-2 aggregates but only into the early pachytene region of the germline (Supplementary Fig. 8b). Thus, HTP-1-dependent checkpoint control of meiotic progression is partially functional in *htp-1*^*∆151-162*^ mutants, but is impaired in *htp-1*^*∆140-162*^ mutants. Surprisingly, however, anti-FLAG staining revealed that both mutant proteins failed to load to axial elements in levels that we could detect cytologically (Fig. 6d), revealing that the β5-αC loop is essential for HTP-1 loading to axial elements and that despite its failure in axis loading, HTP-1^∆151-162^ retains some HTP-1 functionality.

The ColabFold predicted structures of HTP-1^∆151-162^ and HTP-1^∆140-162^ acquire an “empty closed” conformation, similar to WT HTP-1 (Fig. 6e and Supplementary Fig. 8c). Thus, these structural predictions fail to clarify why HTP-1 loop mutants are not loaded to axial elements. Our MD simulations shown in Fig. 5 suggest that in its most downward position, the β5-αC loop comes into close proximity of the safety belt, potentially stabilising the closed conformation. We refer to this as a “closed capped” conformation (Fig. 6f). By examining our simulations to identify residues expected to promote or stabilise the lowered position of the β5-αC loop, we found that D144 within the β5-αC loop is predicted to form a stable interaction with K106 on β4 (Fig. 6g). In the HTP-1^mut7^ simulations, D144 also comes into close proximity to residue K128 on β5, potentially further stabilising the lowered position of the loop (Fig. 6g). In addition, L156 on the short alpha helix at the C-terminal region of the β5-αC loop anchors this region to the HORMA domain by sitting in a hydrophobic pocket (Fig. 6h). We reasoned that introducing a charge reversal at position 144 (D144K) and introducing a charge at position 156 (L156K), would disrupt these interactions and interfere with the downwards movement of the β5-αC loop. Using CRISPR, we generated an *htp-1*^*D144K L156K*^ allele to investigate the effect of these mutations in vivo. *htp-1*^*D144K L156K*^ mutants displayed increased levels of embryonic lethality, synapsis defects, and the accumulation of nuclei with PLK-2 aggregates in the pachytene region, although most nuclei contained 6 bivalents (Fig. 6i and Supplementary Fig. 8d-f). Importantly, loading of HTP-1^D144K L156K^::FLAG to pachytene axial elements was significantly reduced (Fig. 6j), providing further evidence that the β5-αC loop region modulates the loading of HTP-1 and suggesting that it may do so by interacting with residues in the HORMA core.

As the presence of an extended β5-αC loop is a conserved feature of meiotic HORMADs, we asked if this region is also required for chromosomal loading of HORMADs beyond HTP-1 by deleting the β5-αC loop of HTP-3. Similar to HTP-1, deletion of the β5-αC loop in HTP-3 hindered chromosomal loading and crossover formation (Fig. 6k-l). Thus, the β5-αC loop is required to enable chromosomal loading of meiotic HORMADs.

### Closure motif binding and the β5-αC loop are required for HTP-1 stability in vitro and in vivo

**Fig. 7:**
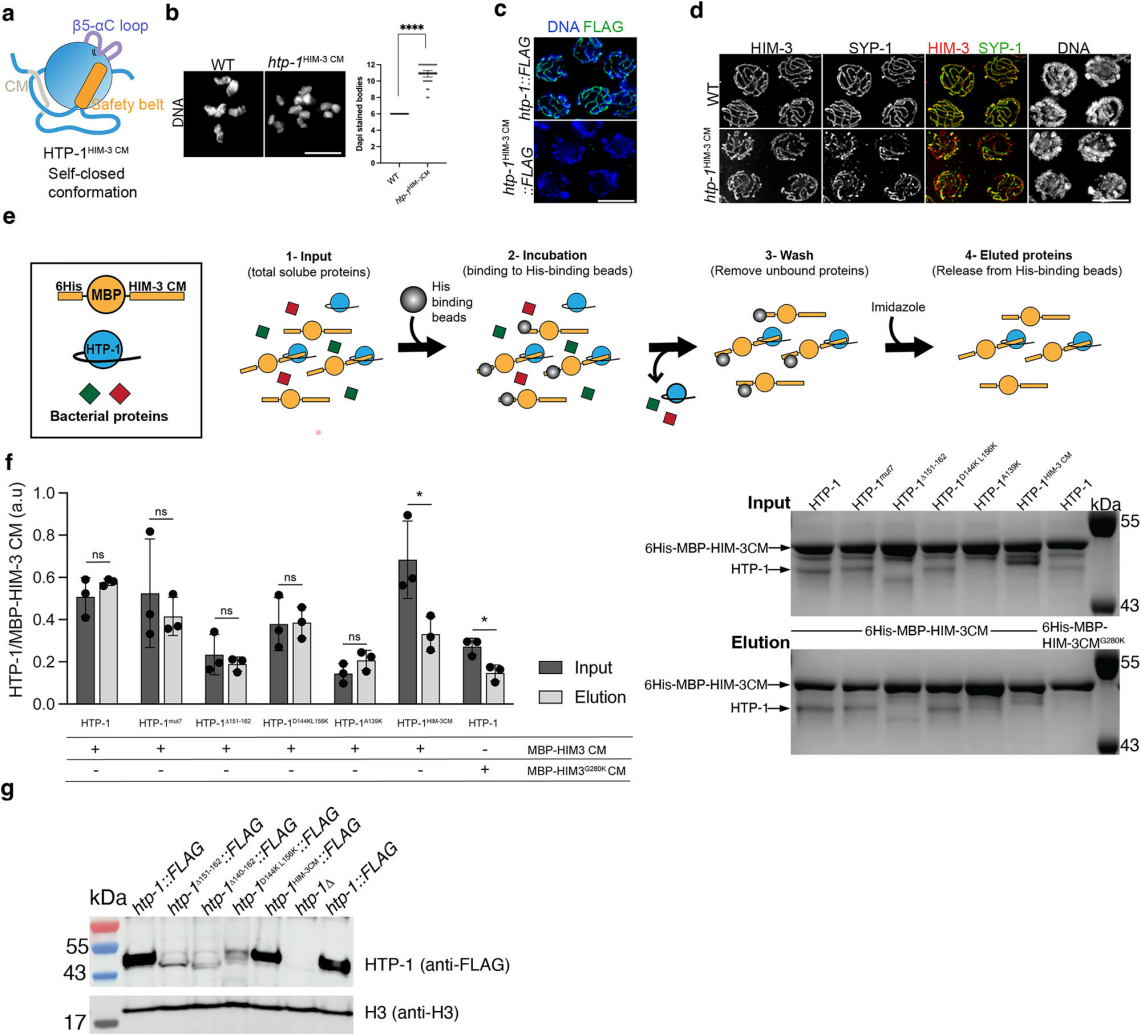
The position of the β5-αC loop and the safety belt determine the conformation of HTP-1 and HTP-3. **a** Cartoon of mHORMAD in a self-closed conformation binding to a closure motif on its C-terminal tail. **b** Projections of diakinesis oocytes of indicated genotypes stained with DAPI. Graph shows quantification of number of DAPI-stained bodies per genotype: WT = 24; *htp-1*^*HIM-3 CM*^ = 34, error bars indicate mean with 95% CI, p values were calculated using a two-tailed Mann-Whitney U test (**** indicates *p* < 0.0001). **c** Projections of pachytene nuclei of indicated genotype stained with anti-FLAG antibodies (HTP-1) and DAPI, note absence of axis staining in *htp-1*^*HIM-3 CM*^ mutants. **d** Projections of pachytene nuclei of indicated genotype stained with anti-HIM-3 (axis component) and anti-SYP-1 (SC component) antibodies and DAPI. Note extensive asynapsis in *htp-1*^*HIM-3 CM*^ mutants. Scale bar = 5 µm in all panels. **e** Diagram of pulldown experiment from total soluble protein extracts from *E. coli* cultures co-expressing different versions of HTP-1 and 6His-MBP-HIM-3CM. **f** Graph shows quantification of protein band intensity of indicated HTP-1 versions comparing the initial amount of HTP-1 in total protein extracts (input) with the amount of HTP-1 following 6His pulldown (elution) from 3 gels. HTP-1 amount are presented as a fraction of 6His-MBP-HIM-3CM levels from the same experiment. Error bars indicate mean with SD, *p* values were calculated using an unpaired two tailed t-test (* indicates *p* < 0.05). Note that highest levels of HTP-1 in input extracts correspond to HTP-1^HIM-3CM^ and that WT HTP-1 input levels are reduced when co-expressed with the mutant version of 6His-MBP-HIM-3CM^G280K^. Gels on the right-hand panels show examples of input (top) and elution (bottom) protein extracts. The same amount of total protein was loaded in each lane and gels were stained with Coomassie blue**. g**) Western blot of total proteins extracts from 100 worms of indicated genotypes probed with anti-FLAG antibodies. Note the reduced HTP-1 proteins levels in the three loop mutants (*htp-1*^*∆140-162*^*::FLAG*, *htp-1*^*∆151-162*^*::FLAG*, and *htp-1*^*D144K L156K*^*::FLAG*) but that HTP-1 levels in extracts from *htp-1*^*HIM-3 CM*^*::FLAG* mutants are similar to WT (*htp-1::FLAG*) controls. Source data are provided as a Source Data file.

To clarify how the β5-αC loop promotes chromosome loading of mHORMADs we used an in vitro system based on the ability of HTP-1 to bind the CM located at the C-terminus of HIM-3^24^. We created bacterial strains co-expressing different mutant versions of HTP-1 and a 6His-MBP-HIM-3CM fusion protein to compare their ability to bind HIM-3’s CM. In addition to WT HTP-1, we expressed the loop mutants HTP-1^∆151-162^, HTP-1^D144K L156K^, HTP-1^A139K^ (see below), the HTP-1^mut7^ carrying mutations in the safety belt region described in Fig. 4, and a version of HTP-1 carrying HIM-3’s CM at its C-terminus (HTP-1^HIM-3 CM^), reasoning that this protein may lead to the formation of a “self-closed” HTP-1 (Fig. 7a) that could interfere with binding CMs on other proteins. In agreement with this possibility, a CRISPR-generated strain expressing *htp-1*^*HIM-3 CM*^ from the endogenous *htp-1* locus displayed no detectable HTP-1 loading to axial elements, resulting in impaired chiasma formation and extensive SC defects (Fig. 7b-d), reminiscent of phenotypes observed in *htp-1∆* mutants (Fig. 1a and Supplementary Fig. 2b). However, unlike *htp-1∆* mutants, *htp-1*^*HIM-3 CM*^ mutant germlines accumulate nuclei with clustered chromosomes and multiple PLK-2 aggregates (Supplementary Fig. 9a). Thus, although HTP-1^HIM-3 CM^ fails to load to axial elements, it appears to retain some HTP-1 functionality as it delays meiotic progression in the presence of synapsis defects.

The different HTP-1 versions were co-expressed with 6His-MBP-HIM-3 CM in *E. coli* and the pool of CM-bound HTP-1 was recovered following pull down of 6His-MBP-HIM-3CM from total soluble protein extracts (Fig. 7e). MBP fused to a mutant version of HIM-3 CM that prevents HTP-1 binding in vivo and in vitro (G280K)^24^ was used as a negative control for HTP-1 binding. Comparison of total HTP-1 levels (input) in the three conditions expressing WT HTP-1 provided important insight into the effect of CM binding in protein stability: HTP-1^HIM-3 CM^ displayed the highest protein levels in input extracts, while levels of HTP-1 were severely reduced when it was co-expressed with the G280K mutant version of HIM-3 CM (Fig. 7f). This suggests that binding to a CM in cis (HTP-1^HIM-3 CM^) or in trans (HTP-1 bound to MBP-HIM-3 CM) is essential for HTP-1 stability in bacteria. In support of this, the amount of HTP-1 was very similar before and after 6His-MBP-HIM-3CM pulldown, suggesting that most HTP-1 present in the input extracts is bound to 6His-MBP-HIM-3 CM (*i.e*. input extracts contain very little amounts of unbound HTP-1). In contrast, the amount of HTP-1^HIM-3 CM^ was significantly reduced after 6His-MBP-HIM-3 CM pulldown, consistent with most HTP-1^HIM-3 CM^ engaging in a, stable, self-closed conformation that interferes with binding the CM linked to 6His-MBP. Input HTP-1 levels were strongly reduced for the loop mutants HTP-1^∆151-162^ and HTP-1^A139K^ (see below), but in both cases HTP-1 levels were similar before and after MBP pulldown, consistent with most HTP-1 in input extracts being associated with MBP-HIM-3 CM (Fig. 7f). HTP-1^D144K L156K^ displayed a slight reduction in input levels, while input levels of HTP-1^mut7^ were similar to WT HTP-1, in agreement with our observations in pachytene axial elements (Figs. 6j and 4e). Levels of these proteins were similar before and after MBP pulldowns, consistent with most HTP-1^D144K L156K^ and HTP-1^mut7^ detected in input extracts being bound to MBP-HIM-3 CM. Importantly, the amount of 6His-MBP-HIM-3 CM was similar between all conditions and was higher than HTP-1 amount in all co-expression experiments, showing that MBP-HIM-3 CM levels was not a limiting factor in determining HTP-1 protein levels. These findings show that, when expressed in bacteria, HTP-1 stability requires CM binding, even in the case of WT HTP-1. Therefore, HTP-1 mutations that interfere with CM binding would be expected to reduce protein levels when expressed in bacteria.

Prompted by our observations with bacterially-expressed HTP-1, we investigated whether mutations in HTP-1’s β5-αC loop that eliminate (HTP-1^∆140-162^, HTP-1^∆151-162^) or reduce (HTP-1^D144K L156K^) loading to axial elements also affect protein levels in vivo. To test whether impaired axis loading per se affects protein levels, we also included in this analysis worms expressing HTP-1^HIM-3 CM^, which fails to bind axial elements (Fig. 7c), but can bind the CM at its C-terminus (Fig. 7f). We performed total protein extracts from *htp-1::FLAG* (WT), *htp-1*^*∆151-162*^*::FLAG*, *htp-1*^*∆140-162*^*::FLAG, htp-1*^*D144K L156*^*::FLAG*, and *htp-1*^*HIM-3 CM*^*::FLAG* worms. Western blot probing with anti-FLAG antibodies demonstrated that protein levels were severely reduced for both loop deletion mutants (*htp-1*^*∆151-162*^*::FLAG* and *htp-1*^*∆140-162*^*::FLAG*) and reduced to a lesser extent for *htp-1*^*D144K L156*^ mutants (Fig. 7g and Supplementary Fig. 9b). These observations align well with our finding that HTP-1^D144K L156K^ is detected at reduced levels at axial elements (Fig. 6j), while no axis signal was detected for HTP-1^∆140-162^ or HTP-1^∆151-162^ (Fig. 6d). Strikingly, despite its failure in axis loading (Fig. 7c), levels of HTP-1^HIM-3 CM^ were similar to control WT HTP-1 (Fig. 7g and Supplementary Fig. 9b), reminiscent of our observations in vitro showing the high stability of HTP-1^HIM-3 CM^ (Fig. 7f). The high protein levels of HTP-1^HIM-3 CM^ demonstrate that impaired loading to axial elements per se is not sufficient to reduce HTP-1 levels, suggesting that the primary reason for the reduced levels of HTP-1 loop mutants may be its reduced ability to bind CMs to acquire a stable, CM-bound, closed conformation.

### Mutant analysis of β5-αC loop in HTP-3 and HTP-1 identifies key residues required for axis loading and protein stability

**Fig. 8:**
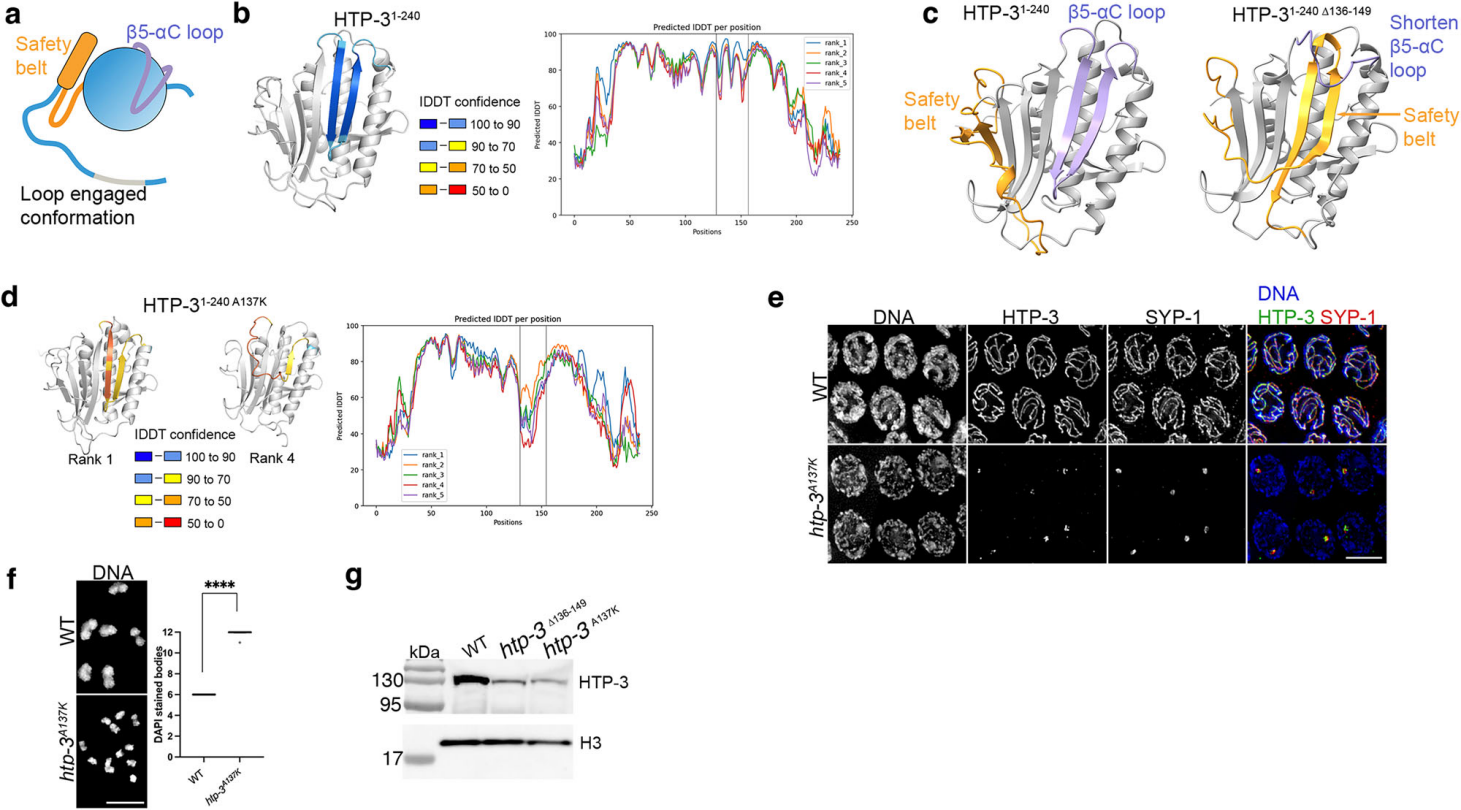
The β5-αC loop is required for HTP-3 function. **a** Cartoon of mHORMAD in a loop engaged conformation. **b** ColabFold prediction of WT HTP-3 HORMA domain indicating IDDT confidence for the β5-αC loop according to colors indicated on key. Graph shows the IDDT confidence per position with vertical lines indicating the position of the β5-αC loop and colors indicating IDDT of five different models (ranks 1-5). **c**) Predicted structures of HTP-3^1-240^ and HTP-3^1-240 *∆136-149*^ (β5-αC loop deleted) indicating the position of the safety belt (orange) and the β5-αC loop (purple). Note that deletion of the β5-αC loop results in relocation of the safety belt to form an empty closed conformation. **d** ColabFold prediction of HTP-3^A137K^ HORMA domain indicating IDDT confidence for the β5-αC loop according to colors indicated on key. Models corresponding to rank 1 and rank 4 are shown. Graph shows the IDDT confidence per position with vertical lines indicating the position of the β5-αC loop and colors indicating IDDT of five different models (ranks 1-5). Note decreased IDDT confidence for the position of the β5-αC loop in HTP-3^1-240 A137K^ compared to HTP-3^1-240^ in (**a**). **e** Projections of pachytene nuclei of indicated genotype stained with anti-HTP-3 pS285 and anti-SYP-1 (SC component) antibodies and DAPI. Note that HTP-3^A137K^ fails to form axial elements and colocalizes with SYP-1 in nuclear aggregates. **f** Projections of diakinesis oocytes of indicated genotypes stained with DAPI. 6 DAPI-stained bodies indicate normal crossover formation, while higher numbers indicate a crossover defect. Graph shows quantification of number of DAPI-stained bodies per genotype WT = 24; *htp-3*^*A137K*^ = 36, error bars indicate mean with 95% CI, p values were calculated using a two-tailed Mann-Whitney U test (**** indicates *p* < 0.0001). **g** Western blot of total proteins extracts from 100 worms of indicated genotypes probed with anti-HTP-3 antibodies. Note reduced HTP-3 proteins leves in the two loop mutants (*htp-3*^*∆136-149*^ and *htp-3*^*A137K*^). Scale bar **=**5 µm in all panels. Source data are provided as a Source Data file.

The finding that the β5-αC loop is essential for HTP-1 and HTP-3 loading and stability led us to further explore how this flexible region impacts on HORMAD function. We started by predicting the structure of HTP-3’s HORMA domain to determine if, similar to HTP-1, the β5-αC loop of HTP-3 could also move downwards to cap the safety belt region in an empty closed conformation. Interestingly, the β5-αC loop of HTP-3 is predicted to fold down over the backbone of the HORMA domain forming a two-stranded beta sheet that forms hydrogen bonds with residues in β5, taking the place of the safety belt in a canonical closed conformation, while the safety belt region remains unbound to the core (Fig. 8a-b). We refer to this predicted HORMAD conformation as “loop engaged” conformation (Fig. 8a). This structural prediction suggests that the β5-αC loop and the safety belt may compete to bind the same region in the core. Supporting this, HTP-3’s HORMA domain lacking the β5-αC loop (HTP-3^∆136-149^) is predicted to fold into an empty closed conformation (Fig. 8c). Next, we examined the predicted HTP-3 loop engaged structure, aiming to identify residues in the β5-αC loop that may be involved in interactions required for this conformation. We assessed that introducing a charge at position A137, located in the middle of the beta strand running anti-parallel to β5, could destabilise the interaction between the loop and the HORMA core. Introducing the A137K substitution in HTP-3’s loop caused a strong decrease in the confidence of the predicted position of the loop (Fig. 8d). Thus, we generated *htp-3*^*A137K*^ homozygous mutants to investigate the impact of the A137K mutation in vivo. Strikingly, HTP-3^A137K^ completely fails to load to chromosomes, impairing synapsis and chiasma formation (Fig. 8e-f), as expected when HTP-3 is not loaded to axial elements^12^. HTP-3^A137K^ is detected in nuclear aggregates colocalizing with SC component SYP-1 (Fig. 8e), suggesting that HTP-3^A137K^ is present in the nucleus but unable to form axial elements. Western blot analysis showed that HTP-3^A137K^ is present at much lower levels than WT HTP-3, but at levels similar to a mutant protein (HTP-3^∆136-149^) in which most of the β5-αC loop is deleted (Fig. 8g). Thus, the β5-αC loop is also essential for HTP-3 loading to axial elements and this process can be disrupted by a single amino acid substitution (A137K) in the β5-αC loop. Moreover, the finding that the A137K mutation prevents axis loading and reduces HTP-3 protein levels suggests that, similar to our observations in HTP-1, the β5-αC loop of HTP-3 may be required for the acquisition of a stable, CM-bound, closed conformation.

**Fig. 9:**
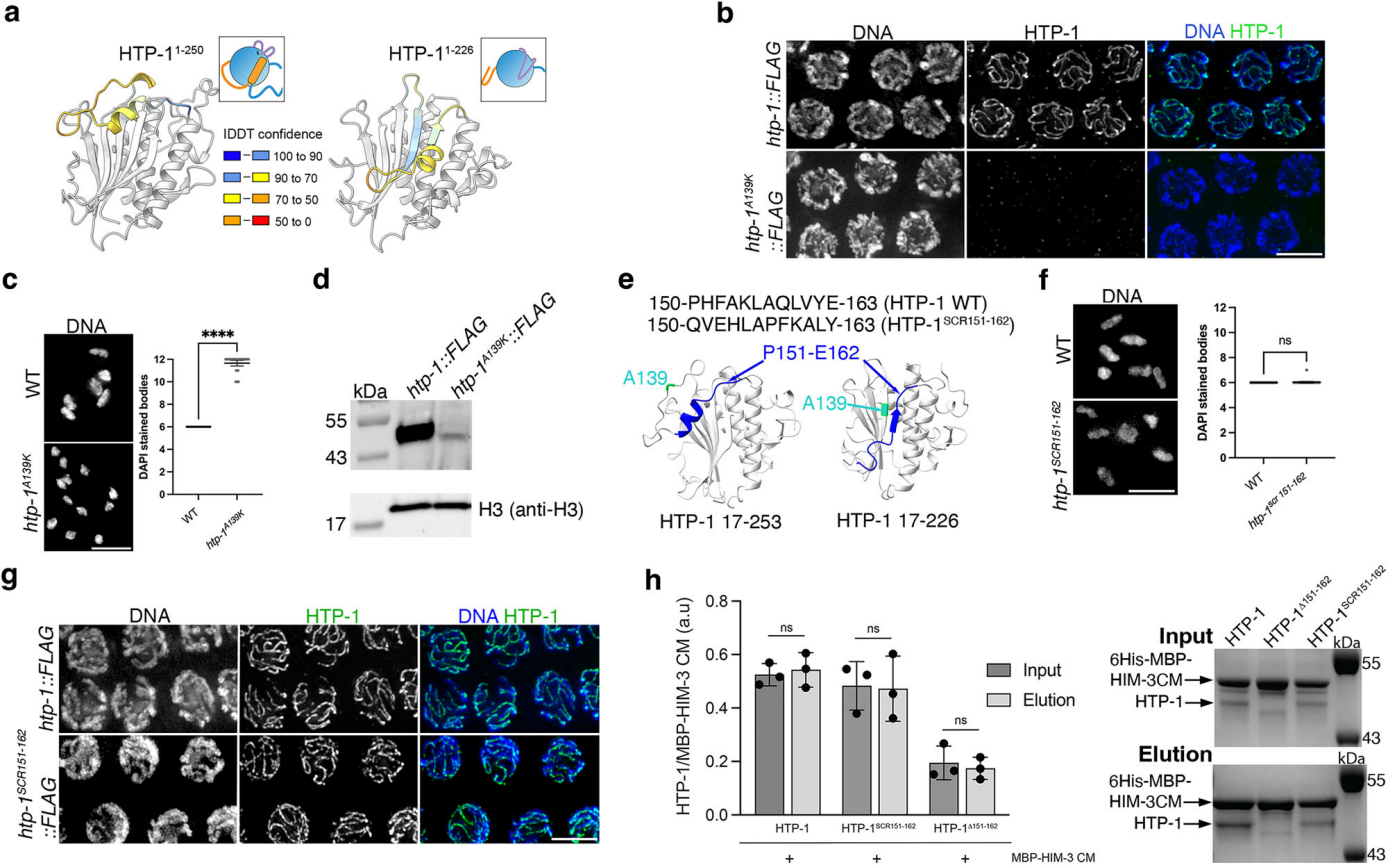
Mutational analysis of HTP-1 β5-αC loop. **a** ColabFold predictions indicating IDDT confidence for the β5-αC loop in HTP-1^1-250^ (models as empty closed conformation) and HTP-1 ^1-226^ (models as loop engaged conformation). **b** Projections of pachytene nuclei of indicated genotype stained with anti-FLAG antibodies (HTP-1) and DAPI. **c** Diakinesis oocytes stained with DAPI (left) and quantification (right). Number of nuclei WT = 22; *htp-1*^*A139K*^ = 32, error bars indicate mean with 95% CI, p values were calculated using a two-tailed Mann-Whitney U test (**** indicates *p* < 0.0001). **d** Western blot of total proteins extracts probed with anti-FLAG antibodies. **e** Predicted structures of WT (left) and safety belt deleted (right) HTP-1 HORMA domain indicating the position of A139 (green) and residues P151 to E162 in the β5-αC loop (blue). Amino acid sequences indicate the WT sequence between P151 and E162 (top) and the scrambled sequence used to create the *htp-1*^*SCR151-162*^ allele (bottom). **f** Diakinesis oocytes of indicated genotypes stained with DAPI (left) and quantification (right). Number of nuclei WT = 25; *htp-1*^*SCR151-162*^ = 30, error bars indicate mean with 95% CI, p values were calculated using a two-tailed Mann-Whitney U test. **g** Projections of pachytene nuclei of indicated genotype stained with anti-FLAG antibodies (HTP-1) and DAPI. Note that HTP-1^SCR 151-162^ associates with axial elements. **h** Graph shows quantification of protein band intensity of indicated HTP-1 versions comparing the initial amount of HTP-1 in total protein extracts (input) with the amount of HTP-1 following 6His pulldown (elution). HTP-1 amounts are presented as a fraction of 6His-MBP-HIM-3CM levels from the same experiment; three different gels were analyzed for each condition, error bars indicate mean with SD, *p* values were calculated using an unpaired two-tailed t-test. Note that highest levels of HTP-1 in input are similar for WT HTP-1 and HTP-1^SCR 151-162^, while levels of HTP-1^∆151-162^ are reduced. Gels on the right-hand panels show examples of input (top) and elution (bottom) protein extracts. The same amount of total protein was loaded in each lane and gels were stained with Coomassie blue. Scale bar =5 µm in all panels. Source data are provided as a Source Data file.

Our findings with HTP-3 led us to ask whether other HORMADs may also adopt a loop engaged conformation. The predicted structures of HTP-1, HTP-2, HIM-3, yeast Hop1, mouse HORMAD1, and *Arabidopsis* ASY1 HORMA domains all adopted an empty-closed conformation, with the C-terminal region of the safety belt folded into a two-stranded beta sheet that interacts with β5, while the loop remains unstructured in an upwards position (Supplementary Figs. 6a, 10a-d). However, as our structural predictions of HTP-3 suggests that the β5-αC loop and the safety belt may compete to bind to the same region of the HORMA core (Fig. 8c), we also run structural predictions after deleting the safety belt region of HTP-1, HIM-3, Hop1, HORMAD1, and ASY1. Deletion of the safety belt region in all these mHORMADs resulted in structural predictions suggesting a downwards movement of the β5-αC loop, which folded into one or two beta strands to form the “loop engaged” conformation by pairing with β5 on the HORMA core (Fig. 9a and Supplementary Fig. 10a-d). We tested the relevance of these predictions by introducing a mutation equivalent to HTP-3 A137K in the β5-αC loop of HTP-1. Similar to *htp-3*^*A137K*^ mutants, the A139K substitution prevented axis loading of HTP-1 in vivo causing a failure on chiasma formation (Fig. 9b-c). Moreover, HTP-1^A139K^ protein levels were severely reduced both in vivo and in vitro (Fig. 7f and Fig. 9d), as previously observed for HTP-1 loop deletion mutants (Fig. 7f-g). The severe reduction in input protein levels in our bacterial co-expression system with MBP-HIM-3 CM suggests that the primary defect of HTP-1^A139K^ is its inability to bind HIM-3’s CM to adopt a stable closed conformation.

According to our structural predictions the main determinant of the loop engaged conformation is the folding of the β5-αC loop into at least one beta strand that runs anti-parallel to, and interacts with, β5 in the HORMA core. In the case of HTP-1, the N-terminal region of the β5-αC loop (residues 135-142) are predicted to fold into a beta strand, while the C-terminal region remains unstructured (Fig. 9a). If this structural prediction is correct, mutations in the N-terminal region of the β5-αC loop that affect its interaction with β5 should interfere with HTP-1 function, as demonstrated for the HTP-1^A139K^ mutant analysis above, while mutations in the C-terminal region would be expected to have a lesser impact. We tested this possibility by creating a *htp-1* mutant in which residues 151-162 were substituted by a scramble sequence (*htp-1*^*SCR151-162*^) that resulted in 12 amino acid substitutions (Fig. 9e). In contrast to the defects in axis loading and chiasma formation seen in *htp-1*^*A139K*^ mutants (Fig. 9b-c), *htp-1*^*SCR151-162*^ mutants displayed HTP-1 loading to axial elements and chiasmata were present in diakinesis oocytes (Fig. 9f-g). Moreover, using our bacterial co-expression system we observed that input and pulldown levels of HTP-1^SCR151-162^ were similar to WT HTP-1 controls and much higher than those of HTP-1^∆151-162^ in which the scrambled sequence was deleted (Fig. 9h). Therefore, while a single amino acid substitution (A139K) in the N-terminal region of the β5-αC loop impairs HTP-1 function in vivo and in vitro, 12 amino acid substitutions in the C-terminal region of the β5-αC loop result in functional HTP-1 in vivo and in vitro. As deletion of the 12 amino acids changed in HTP-1^SCR151-162^ induces severe defects in vivo and in vitro, these findings suggest that the main role of the C-terminal region of HTP-1’s β5-αC loop is to act as a flexible linker that facilitates interaction of the N-terminal region with β5 in the HORMA core.

Overall, our in vivo and in vitro analysis of β5-αC loop mutants guided by our MD experiments and structural predictions suggests that the ability of the flexible β5-αC loop to acquire secondary structure and interact with the HORMA core is a conserved feature of mHORMADs.

## Discussion

Our findings suggest that mHORMADs have expanded the conformation landscape of their HORMA domain beyond the canonical open and closed conformations found in Mad2, with the extended β5-αC loop playing a key role in this process. Our efforts to determine the mechanistic basis for the functional differences between mHORMAD paralogs HTP-1 and HTP-2 show that these are due to 7 amino acid substitutions within the safety belt region of the HORMA domain, and not to differences in time of expression or amount of protein associated with axial elements. By combining functional in vivo and in vitro studies with molecular dynamics modelling and structural predictions we reveal that the interplay between two structurally flexible regions, the β5-αC loop and the safety belt, which can bind to the same region of the HORMA core are key in determining the conformation and function of these proteins. We clarify how paralogs that display nearly identical crystal structures have acquired different functions in vivo and provide evidence that the role of the β5-αC loop in controlling protein conformation and function is likely a conserved feature of mHORMADs. A recent study shows that the β5-αC loop of Mad2 is also a dynamic region that is involved in the open to closed conformational conversion^47^. Together with our findings, this suggests that the involvement of the β5-αC loop in regulating protein conformation is a conserved regulatory aspect of HORMA proteins.

**Fig. 10:**
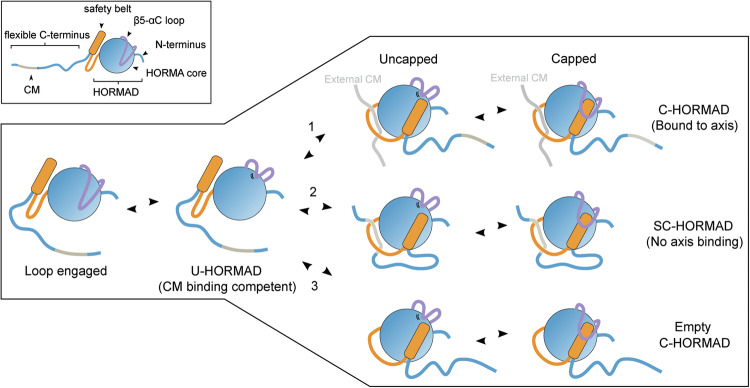
Model: The position of the safety belt and the β5-αC loop determine mHORMAD conformations. CM (Closure motif), U-HORMAD (unbuckled HORMAD), C-HORMAD (closed HORMAD), SC-HORMAD (self-closed HORMAD). See the discussion for a detailed description of how different conformations correlate with HORMAD’s ability to load to axial elements by binding CMs on axis-located proteins.

The model depicted in Fig. 10 summarises how three different positions of the flexible β5-αC loop could lead to the formation of canonical and non-canonical mHORMAD conformations. First, our findings suggest that mHORMADs are capable of forming a loop engaged conformation in which the β5-αC loop moves downwards to interact with β5 on the HORMA core, precluding the safety belt from interacting with this region and therefore interfering with the formation of a canonical closed conformation. Disengaging the β5-αC loop from the HORMA core would lead to an intermediate, such as the previously proposed unbuckled conformation^25^ (U-HORMAD on Fig. 10), that can bind a CM to form a closed conformation bound to a CM (C-HORMAD on Fig. 10). Thus, the loop engaged conformation may act to stabilise a conformation competent to bind a CM, while an interactor bearing a CM is not available. Support for the functional relevance of the loop engaged configuration comes from the observation that a single amino acid substitution (A137K) on the β5-αC loop of HTP-3, as well as the equivalent mutation in HTP-1 (A139K), both predicted to hinder the interaction between the β5-αC loop and the HORMA core, blocks axis loading of HTP-3 and HTP-1 and severely reduce protein levels in vivo and in vitro. In contrast, the C-terminal region of the β5-αC loop of HTP-1, which is not predicted to interact with the HORMA core, tolerates 12 simultaneous amino acid substitutions, suggesting that this flexible region (residues 151-162) acts as a spacer to facilitate the interaction between residues on the N-terminal part of the β5-αC loop and β5. Second, our MD analysis suggests that the β5-αC loop can also move downwards towards the safety belt in the context of a closed conformation, forming a closed capped conformation in which the β5-αC loop is expected to further stabilise the closed conformation. Precedent for this conformation is observed in the structure of closed HIM-3 bound to a HTP-3 CM, in which the β5-αC loop drapes over the safety belt forming ionic and Van der Waals interactions^24^. Third, our MD simulations also suggest that the β5-αC loop can display an upwards position without interacting with the safety belt or the HORMA core. This conformation, referred to as closed uncapped, may represent an intermediate between the closed-capped and unbuckled conformations during the process of binding or releasing CMs on mHORMAD interactors. As depicted in Fig. 10, closed conformations could be formed in cis, by binding to CMs present on the C-terminus of the protein (SC-HORMAD on Fig. 10), in trans, by binding CMs present in other proteins (C-HORMAD on Fig. 10), or even without binding to a CM (empty C-HORMAD on Fig. 9a). The existence of the empty closed conformation is supported by the fact that Mad2^48^, Rev7^19^, and Hop1^25^ can all be purified as empty closed monomers.

Our findings help to clarify why, in contrast to Mad2^48^ and Rev7^19^, a stable open conformation has not been identified for mHORMADs^16,25^. We propose that this is due to the presence of a second structurally flexible region, the extended β5-αC loop, which is much shorter in Mad2 and Rev7 (Supplementary Fig. 1), that can associate with the same HORMA core region as the safety belt. Structural prediction of mHORMAD mutants from different organisms lacking either the safety belt or the β5-αC loop suggests that these two regions could compete to bind the same HORMA core region. For example, *C. elegans* HTP-3 HORMA domain is predicted to adopt a loop engaged conformation with the β5-αC loop interacting with β5 while the safety belt is positioned to the left of β6 on the HORMA core, but deleting the β5-αC loop results in movement and refolding of the safety belt to interact with β5, thereby forming an empty closed conformation. Conversely, the predicted structures of the HORMA domains of *C. elegans* HTP-1, HTP-2 and HIM-3, yeast Hop1, *Arabidopsis* Asy1, and human HORMAD1 all adopted an empty-closed conformation in which the safety belt interacts with β5 and the β5-αC loop is largely unstructured in an upwards position. However, in all these proteins, deletion of the safety belt leads to the prediction of the loop engaged conformation maintained by pairing of the refolded β5-αC loop with β5. Thus, the β5-αC loop and the safety belt may affect the position of each other, and mHORMADs appear to favour conformations in which either of these domains interacts with the HORMA core. For example, expression of Hop1’s HORMA domain in the absence of a CM produces a mix of closed and unbuckled conformations, but expression of a mutant lacking the β5-αC loop displays a single conformation that is consistent with a closed conformation^25^. The strategy of regulating the accessibility of β5 for safety belt binding by modulating the position of a second mobile region is not restricted to mHORMADs. In Mad2, a short β strand (β1) at the N-terminus of the protein pairs with β5 in the open conformation and conversion to closed Mad2 involves displacement and refolding of β1 to allow binding of the safety belt to β5^17,49,50^. Moreover, mutations that hinder the displacement of β1 from β5 lock Mad2 in an open conformation, while mutations that destabilise or eliminate β1 lock Mad2 in a closed conformation, demonstrating how competition for β5 binding between two mobile regions (β1 and the safety belt) controls Mad2 conformation. Thus, the presence of two structurally mobile elements that can be refolded to interact with the same region of the immobile HORMA core appears to be a conserved feature of HORMADs. We propose that in the case of mHORMADs, lengthening of the β5-αC loop has expanded the conformational and functional complexity of these proteins by allowing pairing of the β5-αC loop with β5.

A key finding of our study is that four different mutations (HTP-1^∆140-162^, HTP-1^∆151-162^, HTP-1^D144K L156K^, and HTP-1^A139K^) in the β5-αC loop of HTP-1 reduce or completely prevent axis loading and also severely reduce protein levels in vivo and in vitro. In HTP-3, loop deletion and the A137K mutation, corresponding to HTP-1 A139K, also prevented axis loading and reduced protein levels. Similarly, deletion of the whole loop prevented axis loading and reduced Hop1 levels in vivo in yeast^25^. These observations demonstrate that the loop is essential for axis loading and suggest that axis loading may be required for protein stability. However, we show that expression of HTP-1 carrying a HIM-3 CM at its C-terminus is as stable as WT HTP-1 in vivo and in vitro, but is unable to load to axial elements. Conversely, expression of WT HTP-1 in the presence of a mutant CM that is not bound by HTP-1 severely reduces protein stability in vitro. Moreover, the meiotic defects of worms expressing HTP-1^∆151-162^ (no axis loading) or HTP-1^D144K L156K^ (severely reduced axis loading) are different from those seen in worms lacking HTP-1, demonstrating that these mutations do not fully abrogate HTP-1 function despite their severe effect in overall protein levels. We propose that the primary defect of HTP-1 loop mutants is their inability to bind CMs, which in turn compromises protein stability. Given that HTP-1^A139K^ is as deficient in axis binding and protein stability (in vivo and vitro) as full loop deletion mutants (HTP-1^∆140-162^), we support a model in which interactions between the β5-αC loop and the HORMA core that require A139 are essential to acquire a conformation competent in CM binding, likely the loop engaged conformation. Preventing the interaction between the loop and β5 may result in an empty-closed conformation, analogous to how preventing pairing of β1 with β5 locks Mad2 in a closed conformation. Analysis of loopless Hop1 mutants supports that folding into an empty closed conformation prevents CM binding in vivo^25^. In vitro, loopless Hop1 HORMA domain binds short peptides containing Red1 CM despite forming a closed conformation before the addition of CM peptides. Presumably, this is possible by threading of the CM peptide into the CM binding pocked of closed Hop1. In vivo, however, binding to the CM located in a middle region of Red1 would not be possible without previously unfolding the empty closed conformation. mHORMAD loopless mutants are also expected to be unable to form a closed-capped conformation, which may be important to stabilise a CM-bound closed conformation. Further structural and biophysical studies should elucidate how the loop interacts with the HORMA core to promote a CM binding-competent conformation.

The finding that the lexicon of mHORMADs is not limited to the previously described closed^24,25^ (bound to a CM) and unbuckled^25^ conformations has important implications for elucidating the mechanisms that endow mHORMADS with the ability to control multiple meiotic events. For example, axis-bound mHORMADs are thought to orchestrate quality control of meiotic progression by sensing specific chromosomal states, such as recombination-dependent SC stability^42^, to generate a signal that feeds back on the activity of master meiotic kinases^35,42,51,52^. However, as axis-bound mHORMADs are expected to display a closed conformation bound to a CM^24,25^, it remained unclear if this population of mHORMADs could undergo further morphological changes that may be required to start a signal in response to changes in their local chromosomal environment. Similarly, recent findings suggest that chromosome-bound Hop1 exists in two states, one that promotes DSB formation and is refractory to Pch2 removal and a second that is susceptible to Pch2 and does not promote DSB formation^22^. The nature of these two postulated Hop1 states remains unknown. Our finding that the β5-αC loop remains as a highly flexible region even when HORMADs are in a closed conformation suggest that axis-bound mHORMADs may retain some conformational plasticity. For example, changes in the position of the β5-αC loop of axis-bound HORMADs could change the ability of the loop to act as an interacting surface and expose or hide other interactor-recruiting surfaces. As we have shown that the β5-αC loop of HTP-1 can move downwards to interact with the safety belt, this region is an obvious surface which availability could be regulated by the β5-αC loop. A precedent for this type of interaction is found in closed-conformation human Rev7, where the safety belt provides an interaction surface for Rev1^53^. In addition, both the position of the β5-αC loop and the recruitment of mHORMAD interactors to the loop or to loop-regulated surfaces may be regulated by posttranslational modifications, as HORMADs are known phosphorylation and sumoylation substrates^26,28,54,55^.

In summary, the presence of two flexible protein regions, the β5-αC loop and the safety belt, has expanded the folding complexity of mHORMADs compared to Mad2 or Rev7. We propose that this increased folding complexity, together with the presence of N- and C-terminal domains flanking the HORMA domain endows mHORMADs with the ability to orchestrate many different aspects of meiotic chromosome metabolism, including homologue pairing, recombination, checkpoint control, and release of sister chromatid cohesion. Elucidating how different mHORMAD conformations support specific meiotic events to ensure the formation of haploid gametes remains an important and open question.

## Methods

### *C. elegans* genetics

All *C. elegans* strains were grown on NG agar plates seeded with OP-50 *E. coli* at 20 °C. The N2 Bristol strain was used as the WT reference strain. Transgenic strains containing single-copy transgene insertions of CRISPR-mediated edits were created as described below. Supplementary Table 1 contains the genotype of all strains used in this study.

Strains carrying single copy transgene insertions were created by microinjecting a strain carrying the MosCI transposon at the *ttTi5605* (chromosome II) site^56^. Transgenes expressing chimeric HTP-1/HTP-2 proteins were generated by swapping coding regions in *htp-1* with the corresponding *htp-2* sequence. The amino acid sequence included in each domain is indicated by the first and last amino acid of that domain: N-terminus domain (1-41 aa), C- terminus domain (251-352 aa), HORMA domain (42-250 aa), HORMA A (42-90 aa), HORMA B (91-165 aa) and HORMA C (166-250 aa). Supplementary Table 2 contains a list of transgenes used in this study.

CRISPR-Cas9 mediated genome editing was performed using Cas9 ribonucleoprotein complexes and the co-conversion method using co-editing of *dpy-10* locus as a marker to identify edits^57^. Supplementary Table 3 contains a list of the reagents used for CRISPR-Cas9 genome editing.

### Immunostaining and fluorescence in situ hybridization (FISH)

FISH and immunostaining protocols used in this study were previously described^34^. Briefly, immunostaining was performed in germlines dissected from 19 to 24 h post L4 worms in PBS buffer or EGG buffer (118 mM NaCl, 48 mM KCl_2_, 2 mM CaCl_2,_ 2 mM MgCl_2_, 5 mM HEPES) containing 0.1% Tween and immediately fixed with 1% paraformaldehyde for 5 min. Slides were then snap frozen in liquid nitrogen and following removal of the coverslip, slides were immersed in methanol at -20 °C for at least 1 minute. Slides were then washed three times in PBST (1xPBS, 0.1% Tween) for 5 min and blocked in 0.7% BSA in PBST for 30 min. Primary antibodies were incubated overnight at room temperature or in the cold room for RAD-51 antibodies. After three washes of 10 min in PBST, slides were incubated with secondaries antibodies for 2 h in PBST at room temperature in the dark. Following three washes with PBST, slides were counterstaining with DAPI (2 μg/ml), washed with PBST for 1 h and mounted using Vectashield (Vector). Primary antibodies used in this study: mouse anti-FLAG (M2 monoclonal F1804, Sigma) 1:200, rabbit anti-PLK-2^58^ 1:500, rabbit anti-RAD-51^59^ 1:500, chicken anti-SYP-1^34^ 1:400, rabbit anti-HIM-3^9^ 1:400, rabbit DSB-2^40^, rabbit anti-HTP-3 pS285^60^ 1:1000. Secondary antibodies used in this study: goat anti-rabbit Alexa488-conjugated (Life Technologies), goat anti-chicken Alexa555-conjugated (Life Technologies), and goat anti-mouse Alexa488-conjugated (Life Technologies).

For FISH, germlines were dissected and processed as above, but using 3.7% instead of 1% paraformaldehyde. Following incubation in methanol at -20 °C, slides were washed three times (5 min each) in 2X SSCT (2X SSC 0.1% Tween). Slides were then dehydrated in a series of 70%, 90% and 100% ethanol for three min each and left to dry. The hybridization mix contained 250 ng of labelled probe (5S rDNA locus on chromosome V) in 2 x SSCT, 50 % formamide, 10% w/v dextran sulfate. FISH probes were made by labelling 1 μg of DNA with DIG-nick translation kit (Roche). Following addition of hybridization mix slides were incubated in a thermocycler with the following program: 3 min at 93 °C, 2 min at 72 °C and overnight at 37 °C. Two post-hybridization washes were carried out in 2X SSCT 50% formamide at 37 °C, followed by three 5 min washes in 2X SSCT. Slides were then blocked in 1% BSA 2X SSCT for 1 h. To combine FISH with SYP-1 immunostaining, an overnight incubation with primary chicken anti-SYP-1 antibody in 2xSSCT was performed. The next day, after three washes with 2XSCCT for 10 min, the slides were incubated with goat anti-chicken secondary antibody and Rhodamine-conjugated anti-digoxigenin antibodies (Roche) were added in a 1:100 dilution for 1 h at room temperature in the dark. Slides were finally washed for 30 min and DNA was counterstained with 1 ng/ml DAPI.

Immunostaining and FISH images were acquired as three-dimensional stacks on a Delta Vision system equipped with an Olympus 1×70 microscope using a x100 lens. Images were deconvolved using SoftWoRx 3.0 (Applied Precision) and mounted using Adobe Photoshop.

### Quantification of DAPI-stained chromatin bodies in diakinesis oocytes

Germlines were dissected and stained with DAPI as described in the immunostaining protocol above. The number of DAPI-stained bodies was scored in the two most proximal oocytes (known as -1 and -2) as the level of chromosome condensation in these late diakinesis oocytes facilitates scoring of chromatin bodies.

### Quantification of RAD-51 foci in dissected germlines

Quantification of RAD-51 foci was performed in at least three germlines per genotype. Germlines were divided in six-equal size zones between the start of transition zone and the end of late pachytene and the number of RAD-51 foci was scored per nucleus in each zone. Number of RAD-51 were plotted according the following categories: 0, 1, 2 to 3, 4 to 6, 7 to 12, more than 12, and stretches (agglomerates of RAD-51 signal that were larger than individual rounded foci).

### Quantification of chromosome V FISH signals

FISH signals were quantified by dividing germline into six zones of equal length from the start of transition zone to the end of late pachytene and scoring the number of signals per nucleus. FISH signals were considered paired if a single focus was present in the nucleus and unpaired when two foci were observed in the same nucleus at a distance larger than 0.6 μm (measured in softWoRx Explorer).

### Quantification of SC assembly in dissected germlines

Germlines stained with anti-HIM-3 and anti-SYP-1 antibodies were divided into five zones of equal length from the start of transition zone until the end of late pachytene. HIM-3 and SYP-1 signals were evaluated in individual nuclei of each zone and were scored as synapsed when HIM-3 and SYP-1 signals overlapped along the full length of all chromosomes, while nuclei displaying HIM-3 regions devoid of SYP-1 signal were scored as unsynapsed.

### Quantification of PLK-2 and DSB-2 signals in dissected germlines

Germlines stained with anti-PLK-2 antibodies were divided into five equal-size zones spanning from the start of transition to the end of late pachytene. The number of PLK-2 aggregates per nucleus in each zone was scored as according to the following categories: 0, 1, or more than 1. Quantification of the DSB-2 positive zone of the germline in different genotypes was performed as previously described^40^. Briefly, projections of germlines stained with anti-DSB-2 antibodies and DAPI were divided into vertical rows of nuclei between the onset of transition zone and the end of late pachytene. The extent of the DSB-2 positive zone was determined as the percentage of continuous rows of nuclei in which all or most nuclei displayed DSB-2 staining out of the total rows of nuclei between transition zone and the end of pachytene.

### Quantification of fluorescence signal intensities to measure HTP-1 and HTP-2

Whole-nucleus mean fluorescence intensity was used to compare the fluorescence intensity of anti-FLAG signal in dissected germlines from worms expressing different versions of *htp-1*, and *htp-2* all containing a FLAG just before their STOP codon. Images were acquired on a Delta vision microscope as 3D stacks using the same exposure settings between genotypes to be compared. Quantitative analysis of fluorescence intensity was performed as previously described^61^. Briefly, non-deconvolved images were analysed in Image J using a customized macro. Nuclei were manually circled using the “oval” tool, one nucleus at a time, and the fluorescence intensity of each z-stack slice was measured. The mean fluorescence, displayed as arbitrary units, was calculated after normalising for the number of z-stack slices and the area drawn.

### Statistics and reproducibility

All microscopy experiments were performed at least as two independent replicates per condition/genotype. Between 5 and 20 germlines were observed in each replicate per condition/genotype before acquiring the indicated number of germlines for quantifications or to display representative examples. Statistical analyses were performed using GraphPad Prism (version 10.3.0) software. Sample sizes for cytological analysis were determined based on estimates from preliminary data and on similar studies where whole *C. elegans* germlines or diakinesis oocytes were imaged and used for quantifications. We quantified nuclei from at least 3 different germlines per genotype/condition.

### Quantification of embryonic viability

A single L4 stage hermaphrodite was placed in a plate containing a small drop of OP-50 bacteria, and worms were transferred onto new plates every 12 h. 24 h later, the number of unhatched embryos was scored from each plate. Three days after the parental worm was removed, the total number of hermaphrodite and male progeny reaching L4/ adult stages was scored per plate. Embryonic viability of each biological replicate (at least 5 individual worms were used per genotype) was calculated as the number of hatched eggs over the total number of laid eggs.

### Protein expression and pulldown experiments

HTP-1 (WT and different mutant versions described in Fig. 7f and Fig. 9h) and 6His-MBP-HIM3 CM(RDSPYGLSQGITKKNKD) were co-expressed in BL21 C3013 *E. coli* cells (NEB) using a modified pETDuet-1 plasmid. Pellets from *E. coli* cultures were flash frozen and stored at -80 °C. Pellets to compare the different mutations were then processed at the same time. Cells were resuspended in cold Lysis Buffer (50mMTris-Cl pH 7.4; 200 mM NaCl; 5 mM Imidazole; Roche protease inhibitor without EDTA) and lysed by sonication. Total lysates were centrifuged at 20.000 × g 15 min 4 °C and supernatants were recovered and quantified using Bradford. Between 1.5 to 2 µg of total soluble protein per condition were run in a 12% gel SDS-PAGE to determine the input amounts of HTP-1 and 6His-MBP-HIM-3 CM, then 2 mg of total protein for each condition was incubated with HisPure Cobalt resin for 1.5 h at 4 °C with gentle mixing. Resin was washed 6 times with Wash Buffer (50mM Tris-Cl pH 7.4; 200 mM NaCl; 20 mM Imidazole) and then protein was eluted in 100ul of Elution Buffer (50mM Tris-Cl pH 7.4; 200 mM NaCl; 150 mM Imidazole). Equal amounts of sample for each condition were then run in 12% gel SDS-PAGE stained with Coomassie Blue. The experiment was performed 3 times for each HTP-1 version that was tested.

Quantification of bands corresponding to HTP-1 and 6His-MBP-HIM-3 CM from Coomasie blue-stained gels was performed using ImageJ. Briefly, the same frame size was use for all samples and its corresponding background. Then, pixel values were inverted (255-x; where x is the measured value), background was subtracted and values of the band corresponding to HTP-1 were normalized to the values of the corresponding 6His-MBP-HIM-3 CM band from the same experiment. For each condition, the input and elution was compared using a t test (*p < 0.05; *n* = 3).

#### Western blot from whole-worm protein extracts

100 hermaphrodite worms (24 h post-L4 stage) were collected and placed into 30 µl of TE buffer (10 mM Tris-HCl pH 8, 1 mM EDTA) supplied with complete protease inhibitor cocktail (Roche). Samples were snap-frozen in nitrogen liquid and saved at −80 °C. After thawing, three cycles of freeze and thaw followed by crushing using a pestle were performed until the worms were broken in small pieces. Then, Laemmli buffer supplied with ß-mercaptoethanol was added and samples were boiled at 95 °C for 10 min. Whole extracts were loaded in a precast 4-20% gradient MOPS gel (GenScript Biotech) and proteins were transferred onto nitrocellulose membrane in 1x Tris-Glycine buffer containing 20% of methanol for 1 h at 4 °C and subsequently blocked for 1 h in 5% skim milk TBST (Tris-buffered saline with 0.1% Tween-20) at room temperature. The incubation with primary and secondary antibodies was carried out at 4 °C overnight and 2 h at room temperature, respectively. The following primary antibodies diluted in 5% skim milk TBST were used: guinea pig anti-HTP-3^12^ (1:2500), mouse HRP-conjugated anti FLAG antibody (Sigma, 1:5000) and rabbit anti-Histone 3 (AbCam,1:10000). Additionally, the following secondary antibody conjugated to HRP (5% skim milk TBST) were used: goat anti-guinea pig (Santa Cruz Biotechnology, 1:5000) and goat anti-rabbit (Jackson Laboratories, 1:5000). After exposure to ECL substrates (Bio-Rad), images were rapidly captured with an Amersham ImageQuant 800 system.

### Protein structure prediction methods

Protein structural predictions were performed using ColabFold v1.5.5 AF2 with MMSeqs2. Supposition of different models were performed using PyMOL v2.5.0 (The PyMOL Molecular Graphics System, Version 2.5.0 Schrödinger, LLC) while figures in this manuscript were created using either PyMOL v2.5.0 or ChimeraX v1.6.1.

### Computational modelling and simulation of HTP-1 and HTP-2

The crystal structures of HTP-1 (PDB 4TZQ) and HTP-2 (PDB 4TZM)^24^ were completed by adding the missing residues (residues 6-15, 253) using Modeller 9.02^62^. Residue 84 in HTP-1, which is modified to leucine in the crystal structure, was replaced with proline found in the WT protein using PyMOL v2.5.0 (The PyMOL Molecular Graphics System, Version 2.5.0 Schrödinger, LLC). HTP-1^mut7^ was generated by introducing mutations in WT HTP-1 (D226G, A230V, C239H, N243H, E245Q, M249K, L250S) using PyMOL v2.5.0.

The proteins were solvated by the TIP3P water model and neutralising counterions^63^. Simulations were performed using GROMACS 2018.3^64^ and the CHARMM36m force field^65^. During system setup, histidine residues were modelled in their neutral form, with the Nτ nitrogen farthest from the side chain *α*-carbon protonated in the imidazole ring, consistent with the expected protonation state and the preferred tautomeric form of histidine in proteins at physiological pH^66^. The protein systems were firstly energy minimised using the steepest descent algorithm to relax steric clashes generated during set up. Following this, the systems were equilibrated for a total of 600 ps; the positional restraints (with force constant 1000 kJ mol^−1^ nm^−1^) initially applied to the protein backbone atoms were reduced in a stepwise manner^67^. The production simulations of the unrestrained protein were run for 1000 ns. The average dimensions of the simulation box for all systems are 9.9 × 9.9 × 10.0 nm^3^. For each system, replicate simulations were initiated using coordinates extracted at random time points from the last 100 ps of the equilibration run. The initial coordinates and velocities differ for each replicate simulation.

Simulations were performed using the NPT ensemble, with the pressure maintained semi-isotropically using the Parrinello−Rahman barostat at 1 bar and a time constant of 5 ps^68^, and the temperature sustained at 310 K using the velocity-rescale thermostat and a coupling constant of 0.1 ps^69^. The lengths of all bonds were constrained using the LINCS algorithm enabling a timestep of 2 fs^70^. The Particle Mesh Ewald method was used to treat long-range electrostatic interactions with a short-range cut-off of 1.4 nm^71^. The van der Waals interactions were curtailed at 1.4 nm, with long-range dispersion corrections applied to the pressure and energy. The periodic boundary conditions were applied to all systems in three dimensions, as done previously^67^.

Analyses were performed using GROMACS utilities and locally written code. The molecular graphics images were generated using Visual Molecular Dynamics (VMD) package v1.9.3^72^ and PyMOL v2.5.0. The MD simulation data presented are available at 10.5281/zenodo.20066932.

### Reporting summary

Further information on research design is available in the Nature Portfolio Reporting Summary linked to this article.

## Supplementary information

Supplementary Information

Reporting Summary

Transparent Peer Review file

## Source data

Source Data

## Data Availability

The MD simulation data presented in this study are available at Zenodo (10.5281/zenodo.20066932). The source data underlaying main and Supplementary Figs. are provided in the source data file. *C. elegans* strains created for this study are available from the corresponding author. Source data are provided with this paper.
