# Supplementary material for: Two structurally mobile regions control the conformation and function of metamorphic meiotic HORMAD proteins
Source: Nat Commun. 2026 May 19;17:6615. doi: 10.1038/s41467-026-72656-6 (PMC13381836; doi:10.1038/s41467-026-72656-6)
Supplement: Supplementary file 1 — Supplementary Information [file 41467_2026_72656_MOESM1_ESM.pdf]

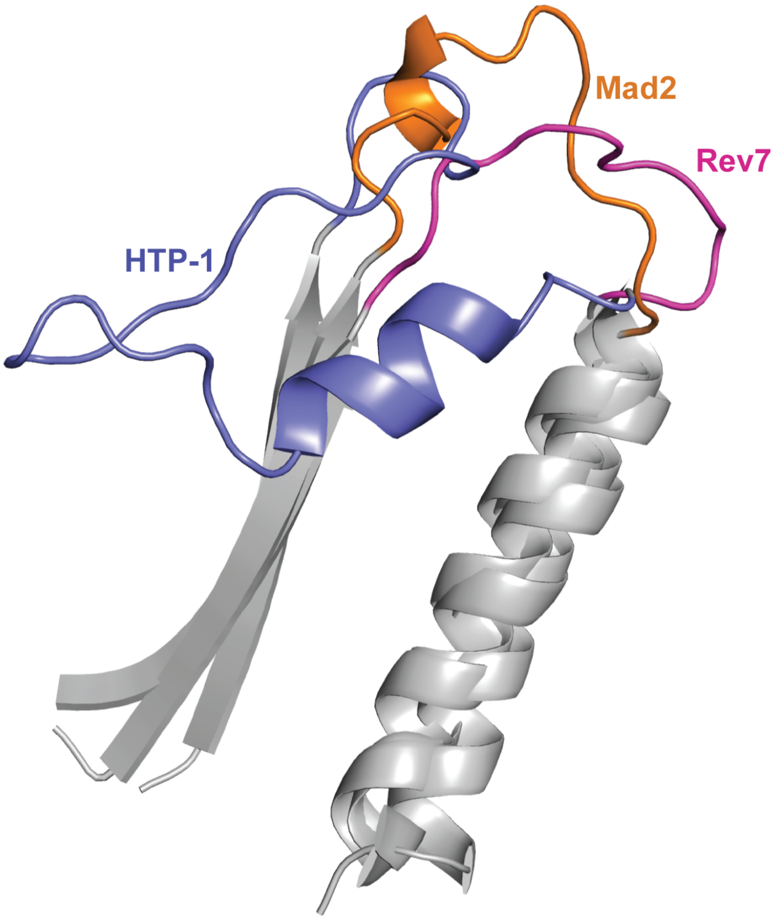

Figure S1. Overlap of the structures of HTP-1, hs Mad2 and hs Rev7 spaning the region between the start of β5 and the end of αC. Note that the loop between β5 and αC is longer in HTP-1 than in Mad2 and Rev7.

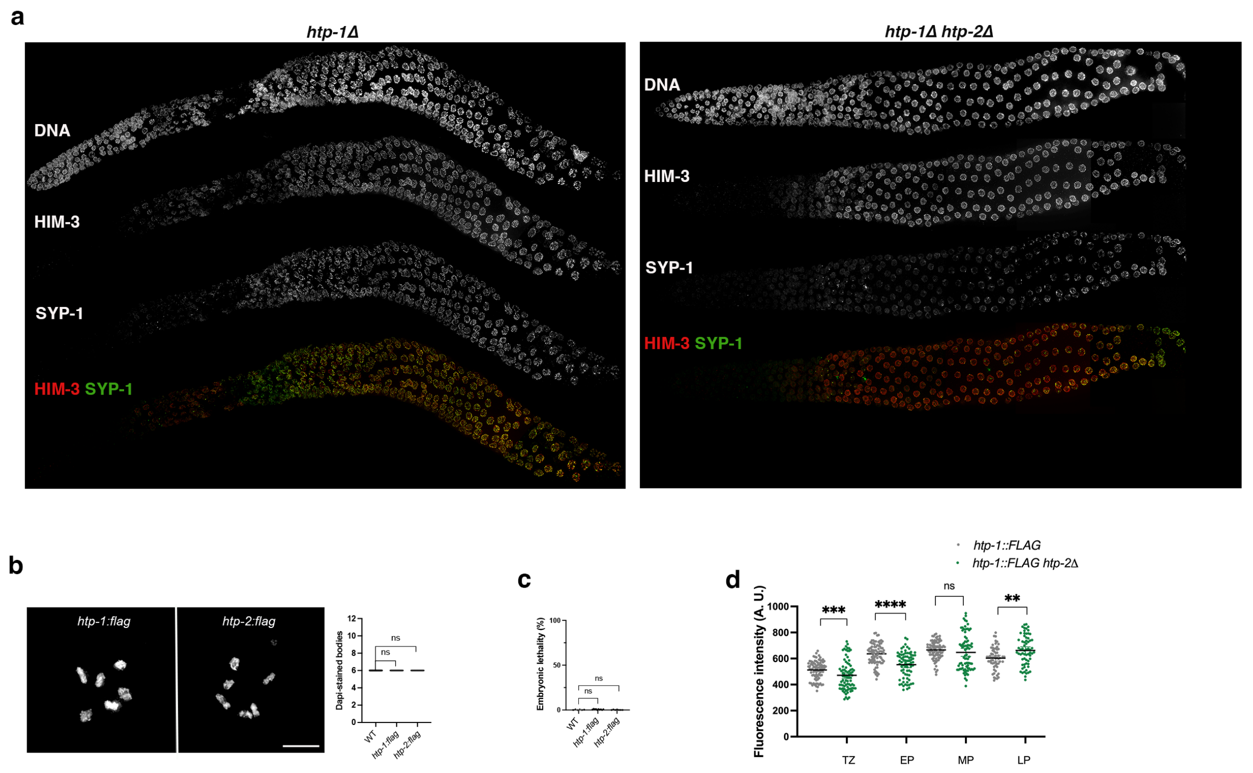

Figure S2. a) Projections of whole-mounted germlines of indicated genotypes stained with anti-HIM3 and anti-SYP-1 antibodies and DAPI. Note that SYP-1 staining is delayed and reduced in *htp-1Δ htp-2Δ* double mutants compared to *htp-1Δ* single mutants. **b)** Projections of diakinesis oocytes from *htp-1::FLAG* and *htp-2::FLAG* homozygous worms stained with DAPI. Note the presence of 6 DAPI-stained bodies, indicating normal crossover formation. Graph shows quantification of number of DAPI-stained bodies per genotype, between 20 and 26 oocytes were analysed per genotype, error bars indicate mean with 95% CI, p values were calculated using a two-tailed Mann-Whitney U test. Scale bar =5 µm. **c)** Quantification of embryonic lethality in strains of indicated genotypes. Number of worms and embryos analysed per genotype: WT= 6, 1707; *htp-1::FLAG*= 6, 1373; *htp-2::FLAG*= 7, 2177; error bars indicate mean with 95% CI, p values were calculated using a two-tailed Mann-Whitney U test. **d)** Graphs show intensity of anti-FLAG staining in nuclei of the indicated germline regions (transition zone (TZ), early pachytene (EP), mid pachytene (MP), and late pachytene (LP)) and genotypes, between 61 and 83 nuclei from four different germlines were analysed per stage and genotype, error bars indicate mean with 95% CI, p values were calculated using a two-tailed Mann-Whitney U test. Source data are provided as a Source Data file.

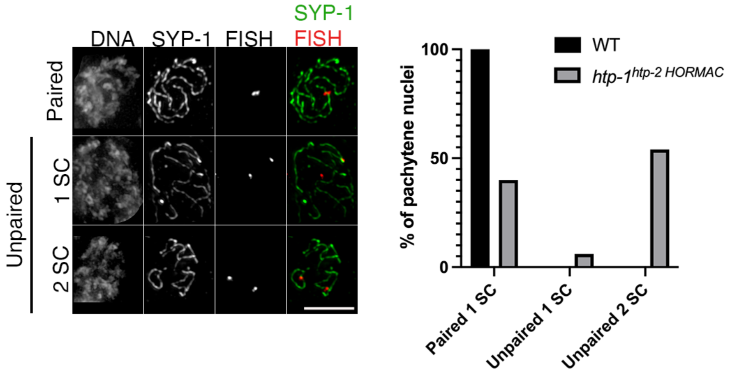

Figure S3. Projections of pachytene nuclei labelled with a FISH probe to visualise the 5S rDNA locus and anti-SYP-1 (Synaptonemal Complex component) antibodies. Two 5S foci, each colocalizing with a different SYP-1 track indicates non-homologous synapsis. Graph shows quantification of the % of nuclei with paired FISH signals (1 focus per nucleus) associated with a SYP-1 track (indicative of homologous synapsis), and unpaired FISH signals (two foci per nucleus) associated with either one or both FISH signals associated with SYP- tracks. Number of nuclei anlysed per genotype: 278 (WT) and 227 (*htp-1*$^{htp\text{-}2\ HORMAC}$). Source data are provided as a Source Data file.

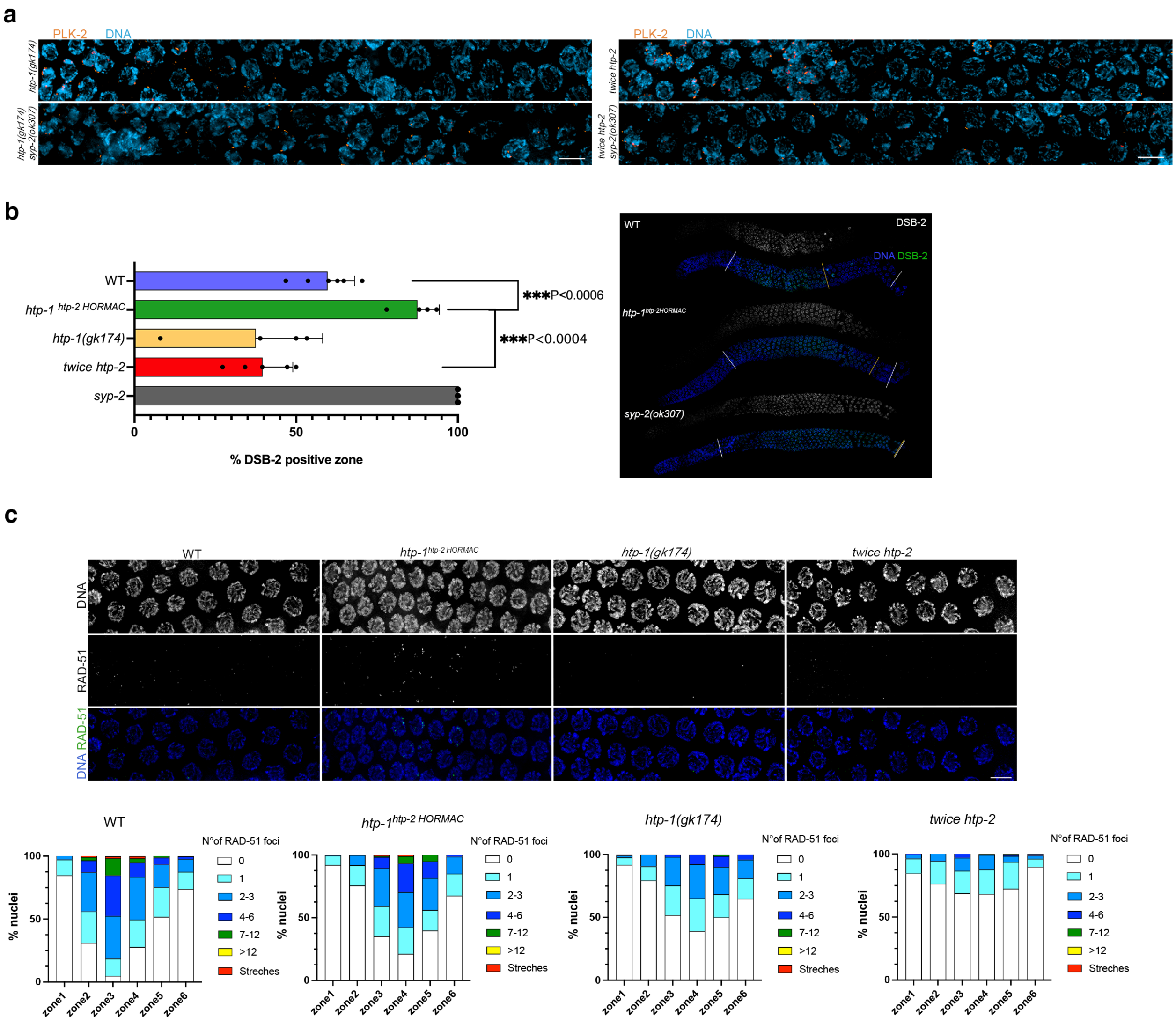

Figure S4. a) Projections of nuclei in transition zone and early pachytene regions of the germline of indicated genotypes stained with anti-PLK-2 antibodies and DAPI (examples of graphs shown in Fig 3c). Nuclei with more than 1 PLK-2 aggregate indicate high CHK-2 activity, 1 PLK-2 aggregate indicates intermediate CHK-2 activity and no PLK-2 aggregate indicates no CHK-2 activity. Note that in all genotypes nuclei with multiple PLK-2 aggregates are lacking in nuclei on the right-hand side of the panels, corresponding to early pachytene. **b)** *htp-1$^{htp\text{-}2\ HORMAC}$*, but not *htp-1(gk174)* or *twice htp-2* mutants accumulate nuclei positive for DSB-2. Graph shows the percentage of vertical rows of nuclei between transition zone and late pachytene that are positive of anti-DSB-2 staining Number of germlines scored: 6 (WT), 4 (*htp-1$^{htp\text{-}2\ HORMAC}$*), 4 (*htp-1(gk174)*), 5 *twice htp-2*, and 3 (*syp-2*). Error bars indicate mean plus SD, p values were calculated using one way ANOVA between *htp-1$^{htp\text{-}2\ HORMAC}$*, *htp-1 (gk174)* and *twice htp-2* and an unpair t test between *htp-1$^{htp\text{-}2\ HORMAC}$* and WT. Panel on the right shows projections of full

germlines stained with anti-DSB-2 antibodies and DAPI. Vertical white lines indicate region between start of transition zone and end of late pachytene, while an orange line indicates the end of the region containing DSB-2-positive nuclei. Number of germlines scored per genotype: WT= 6, *htp-1*$^{htp\text{-}2\ HORMAC}$= 4, *twice htp-2*= 5, *htp-1(gk174)*= 4 and *syp-2*= 3. **c)** Projections of pachytene nuclei (corresponding to zones 4 and 5 of the graphs shown below) from germlines of indicated genotypes stained with anti-RAD-51 antibodies and DAPI. **c)** Quantification of RAD-51 foci per nucleus in germlines of indicated genotypes divided into 6 zones between transition zone and late pachytene as indicated in the cartoon in Figure 3a. Number of nuclei (3 germlines per genotype) analysed per zone= 183, 148, 124, 115, 89, 88 (WT); 151, 136, 122, 118, 98, 74 (*htp-1*$^{htp\text{-}2\ HORMAC}$); 136, 107, 110, 92, 92, 74 (*htp-1(gk174));* 194, 161, 135, 145, 127, 79 (*twice htp-2*). Scale bar =5 µm in all panels. Source data are provided as a Source Data file.

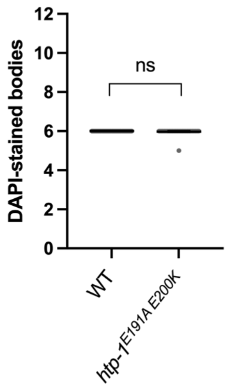

Figure S5. Graph shows quantification of number of DAPI-stained bodies per genotype in WT (n= 51) and *htp-1*$^{E191A\ E200K}$ (n= 49), p values were calculated using a two-tailed Mann-Whitney U test. Source data are provided as a Source Data file.

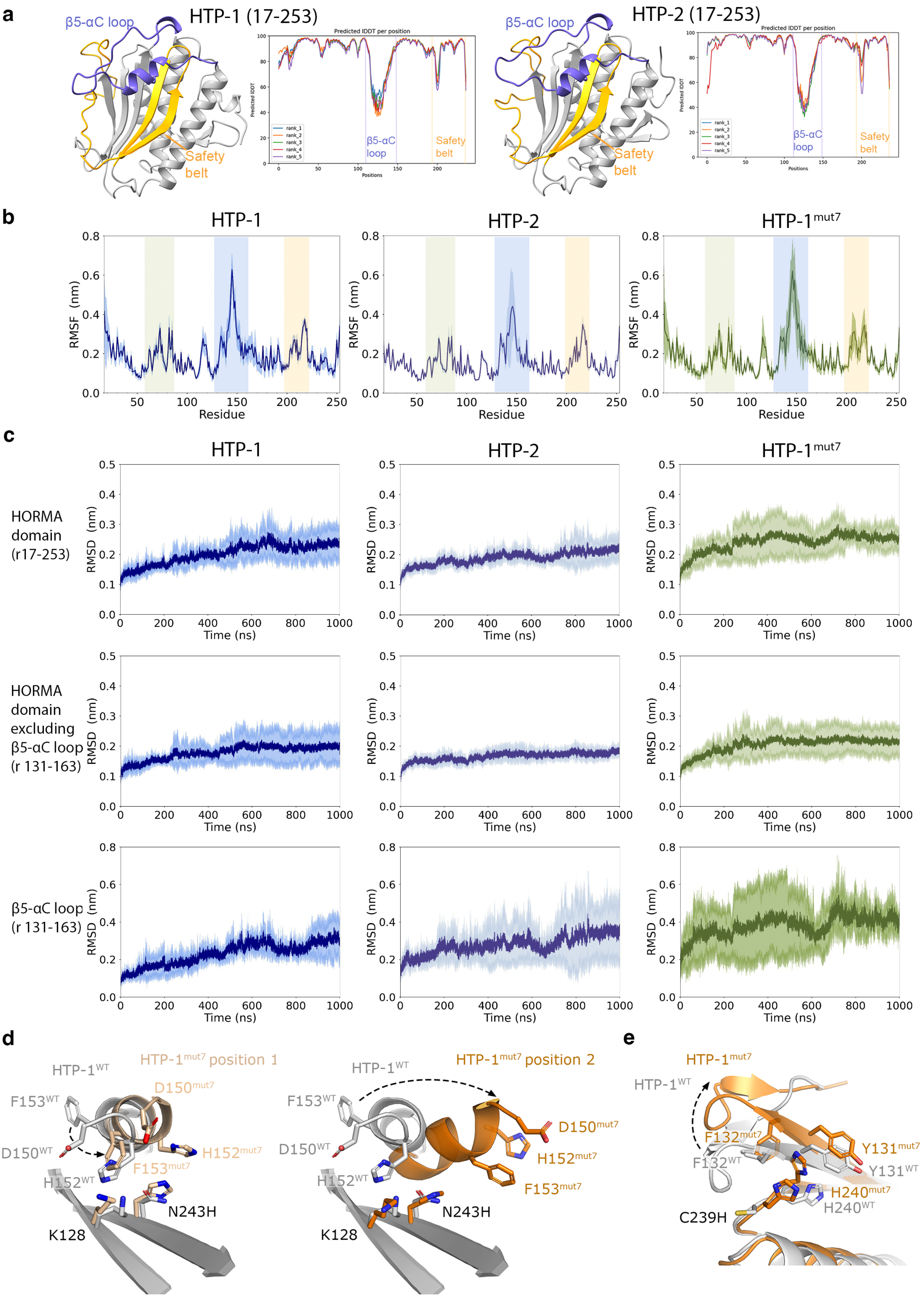
a
HTP-1 (17-253)
β5-αC loop
Safety belt
Predicted lDDT per position
HTP-2 (17-253)
b
HTP-1
HTP-2
HTP-1^mut7
RMSF (nm)
Residue
c
HORMA domain (r17-253)
HORMA domain excluding β5-αC loop (r 131-163)
β5-αC loop (r 131-163)
RMSD (nm)
Time (ns)
d
HTP-1^WT
HTP-1^mut7 position 1
HTP-1^mut7 position 2
F153^WT
D150^WT
H152^WT
D150^mut7
H152^mut7
F153^mut7
N243H
K128
e
HTP-1^mut7
HTP-1^WT
F132^mut7
F132^WT
Y131^mut7
Y131^WT
H240^mut7
H240^WT
C239H

Figure S6. a) ColabFold predictions of HTP-1 and HTP-2 HORMA domains (residues 17-253) indicating the β5-αC loop in purple and the safety belt region in orange. Note that HTP-1 and HTP-2 HORMA domains are predicted to display an empty closed conformation. Graphs show the predicted lDDT per position for the five models (ranks 1-5), indicating high confidence in the position of the safety belt region. **b)** Mean root mean square fluctuation (RMSF) of residues in HTP-1, HTP-2 and HTP-1^{mut7} in 5 independent 1000 ns simulations. Residues highlighted correspond to the β2-β3 hairpin in green, β5-αC loop in blue, and safety belt loop in orange. **c)** Mean root mean square deviation (RMSD) of Cα atoms in HTP-1, HTP-2, and HTP-1^{mut7} over 5 independent 1000 ns simulations. Shading indicates the standard deviation. **d)** Structural alignments of HTP-1 WT (grey) and HTP-1^{mut7} (orange) highlighting the movements observed in the β5-αC loop. During the HTP-1^{mut7} simulations 2 different positions were identified (light orange and dark orange). The alternate positions facilitated interactions of N243 with H152 and F153. **e)** Structural alignment of HTP-1 (grey) and HTP-1^{mut7} (orange) highlighting the movements observed at the end of β5.

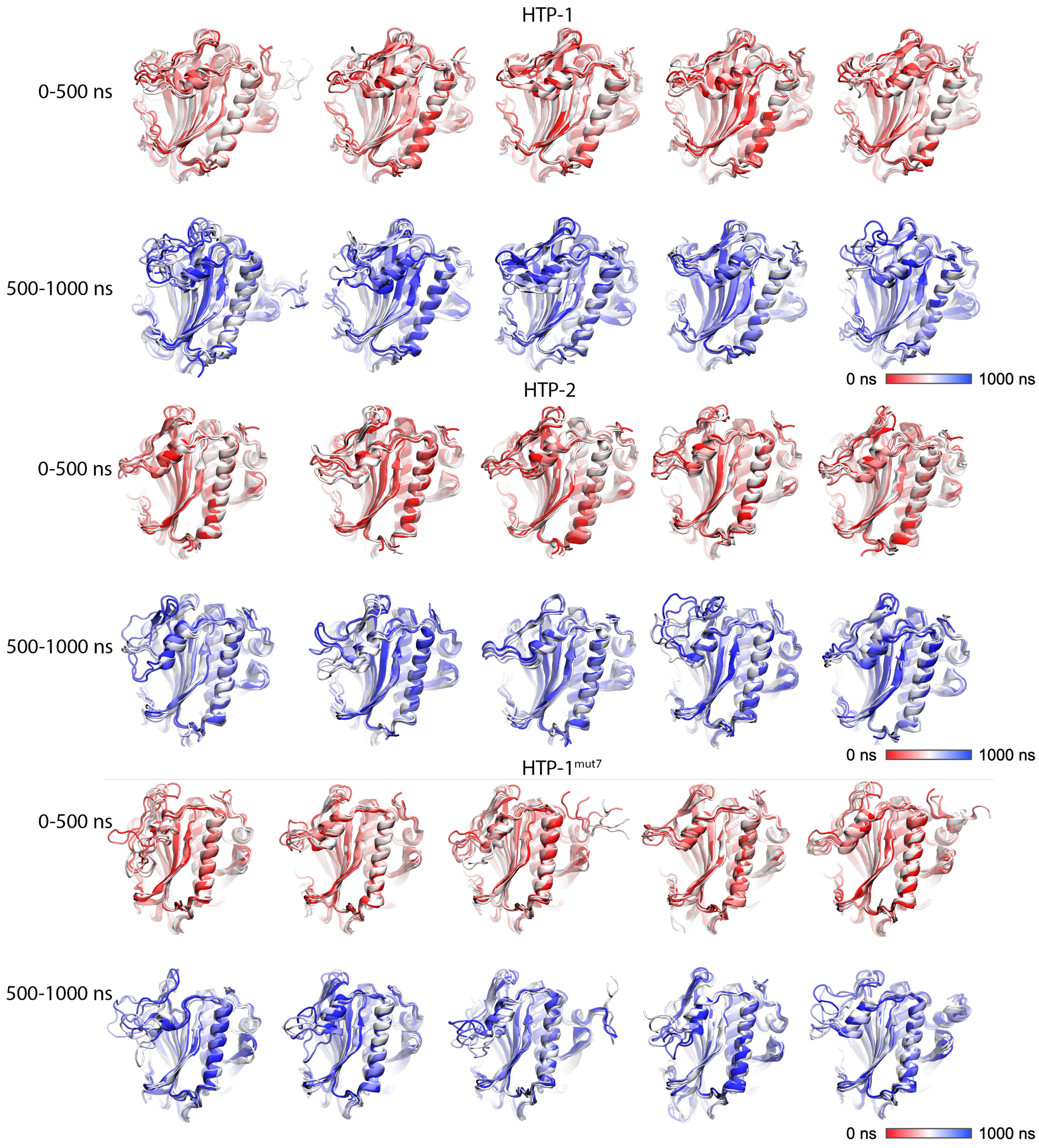

Figure S7. Protein dynamics of HTP-1, HTP-2, and HTP-1^{mut7} over the course of 5 independant 1000 ns simulations. Frames every 100 ns were superimposed and aligned using protein backbone excluding residues within the β5-αC loop. Each column represents one simulation of the protein.

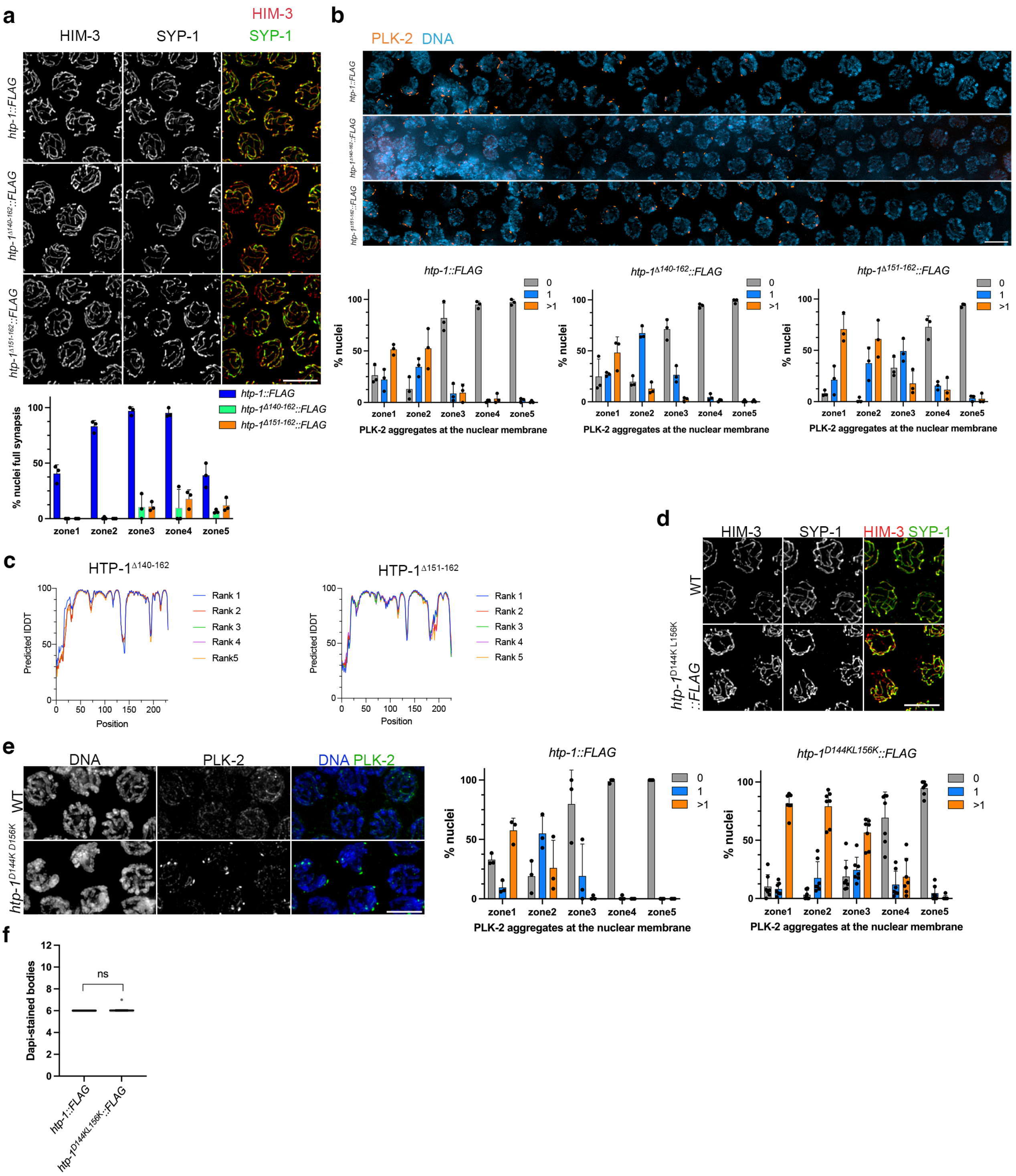

Figure S8. a) Projections of pachytene nuclei stained with anti-HIM-3 (axial element) and anti-SYP-1 (SC component) antibodies. Lines displaying only HIM-3 staining (red signal) indicate the presence of unsynapsed regions. Graph shows quantification of the percentage of nuclei displaying full synapsis (complete overlap of HIM-3 and SYP-1 signals) in five zones along the germline. Note the extensive presence of nuclei with unsynapsed regions in *htp-1*$^{Δ140-162}$*::FLAG* and *htp-1*$^{Δ151-162}$*::FLAG* mutants. Number of nuclei analysed per zone (3 germlines per genotype):

148, 150, 167, 133, 110 (*htp-1::FLAG*)*;* 153, 148, 131, 105, 95 (*htp-1$^{Δ140-162}$::FLAG*); 174, 187, 168, 120, 97 (*htp-1$^{Δ151-162}$::FLAG*) mutants. **b)** Projections of nuclei in transition zone and early pachytene regions of the germline stained with anti-PLK-2 antibodies and DAPI. Nuclei with more than 1 PLK-2 aggregate indicate high CHK-2 activity, 1 PLK-2 aggregate indicates intermediate CHK-2 activity and no PLK-2 aggregate indicates no CHK-2 activity. Graphs show quantification of the % of nuclei with a given number of PLK-2 aggregates in five zones along the germline. Number of nuclei (three germlines per genotype) analysed per zone= 176, 159, 148,138, 127 (*htp-1::FLAG*); 159, 183, 183, 129, 109 (*htp-1$^{Δ140-162}$::FLAG*); 147, 138, 130, 114, 102 (*htp-1$^{Δ151-162}$::FLAG)*. **c)** Graphs showing predicted IDDT per position for five models (ranks 1-5) of indicated HTP-1 loop mutants (models shown in Figure 6e). **d)** Projections of pachytene nuclei stained with anti-HIM-3 (axial element) and anti-SYP-1 (SC component) antibodies. Note that unsynapsed regions are present in *htp-1$^{D144K\ L156K}$::FLAG* mutants. **e)** Projections of pachytene nuclei of WT and *htp-1$^{D144K\ D156K}$* mutants stained with anti-PLK-2 antibodies and DAPI. Nuclei with more than 1 PLK-2 aggregate indicate high CHK-2 activity, 1 PLK-2 aggregate indicates intermediate CHK-2 activity and no PLK-2 aggregate indicates no CHK-2 activity. Graphs show quantification of the % of nuclei with a given number of PLK-2 aggregates in five zones along the germline. *htp-1$^{D144K\ D156K}$* mutants accumulate nuclei with multiple PLK-2 aggregates. Number of nuclei (3 to 7 germlines per genotype) analysed per zone= 176, 148, 129, 121, 96 (WT); 507, 412, 358, 296, 232 (*htp-1$^{D144K\ D156K}$*). **f)** Graph shows quantification of number of DAPI-stained bodies per genotype in *htp-1::FLAG* (n= 40) and *htp-1$^{D144K\ D156K}$::FLAG*= 57 (n= 28), error bars indicate mean with 95% CI, p values were calculated using a two-tailed Mann-Whitney U test. Source data are provided as a Source Data file.

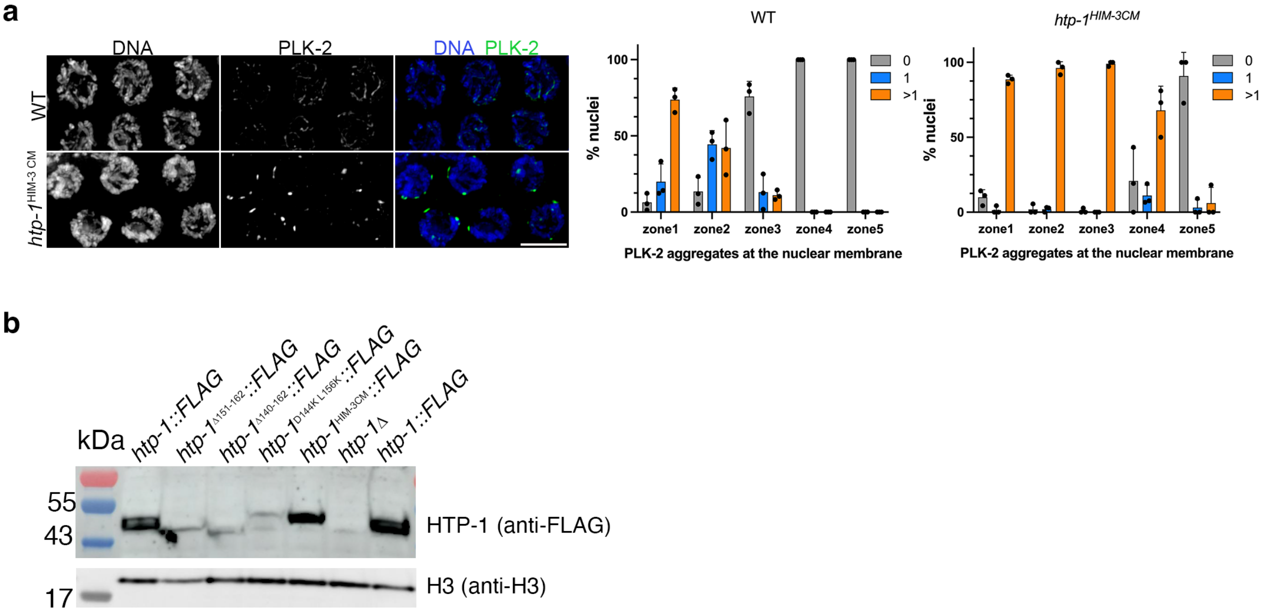

Figure S9. (a) Projections of pachytene nuclei of indicated genotypes stained with anti-PLK-2 antibodies and DAPI. Nuclei with more than 1 PLK-2 aggregate indicate high CHK-2 activity, 1 PLK-2 aggregate indicates intermediate CHK-2 activity and no PLK-2 aggregate indicates no CHK-2 activity. Graphs show quantification of the % of nuclei with a given number of PLK-2 aggregates in five zones along the germline. Note that *htp-1*$^{HIM\text{-}3\ CM}$ mutants accumulate nuclei with multiple PLK-2 aggregates. Number of nuclei (3 to 7 germlines per genotype) analysed per zone= 233, 207, 172, 163, 121 (WT); 183, 150,145, 98, 98 (*htp-1*$^{HIM\text{-}3\ CM}$). **(b)** Western blot of total proteins extracts from 100 worms of indicated genotypes probed with anti-FLAG antibodies. Note the reduced HTP-1 proteins leves in the three loop mutants (*htp-1*$^{\Delta 140\text{-}162}$*::FLAG*, *htp-1*$^{\Delta 151\text{-}162}$*::FLAG,* and *htp-1*$^{D144K\ L156K}$*::FLAG*) but that HTP-1 levels in extracts from *htp-1*$^{HIM\text{-}3\ CM}$*::FLAG* mutants are similar to WT (*htp-1::FLAG*) controls. This is a repetition of the experiment shown in Figure 7g. Source data are provided as a Source Data file.

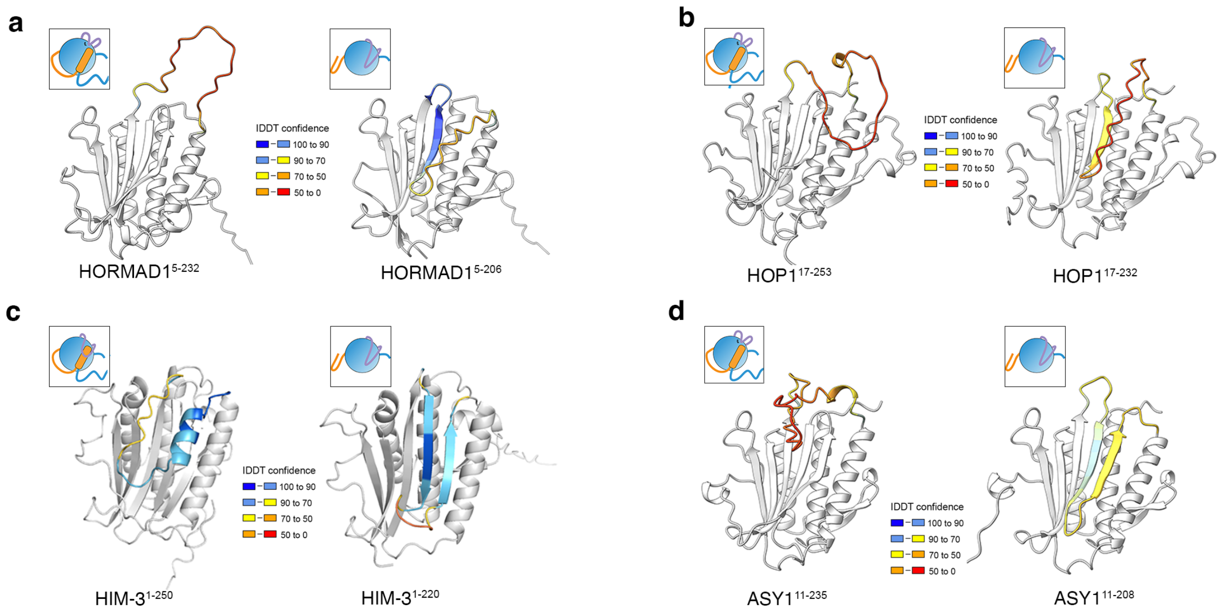

Figure S10. a-d) ColabFold models of the HORMA domain of indicated mHORMADs indicating IDDT confidence values for the β5-αC loop. Model on the left corresponds to WT sequence and model on the right corresponds to structure lacking the safety belt. Note that in all cases deletion of the safety belt resulted in the interaction of the β5-αC loop with β5.

Strains	Genotype	Origin
N2 Bristol	Wild type	CGC
AV393	*htp-1(gk174)/ nT1[unc-?(n754) let-? qIs50] (IV;V)*	(Martinez et al., 2005)[1]
AV276	*syp-2(ok307) V/ nT1[unc-?(n754) let-?(m435)] (IV;V)*	(Colaiácovo et al., 2003)[2]
EG6699	*ttTi5605 II MOSCI; unc119(ed3)III; oxEx1578*	CGC
ATG285	*htp-1 (fq24 [htp-1::FLAG]) IV*	(Sato Carlton et al 2020)[3]
ATG284	*htp-2 (fq26 [htp-2::FLAG]) IV*	This study
ATG224	*htp-2 (fq26[htp-2::FLAG])IV; htp-1(gk174)/ nT1[unc-? (n754) let-? qls50] (IV;V)*	This study
ATG718	*fqSi6 II; htp-1(gk174) IV*	This study
ATG711	*fqSi27 II; htp-1(gk174) IV*	This study
ATG712	*fqSi28 II; htp-1(gk174) IV*	This study
ATG713	*fqSi30 II; htp-1(gk174)/ nT1[unc-? (n754) let-? qls50] (IV;V)*	This study
ATG719	*fqSi22 II; htp-1(gk174) IV*	(Ferrandiz et al., 2018)[4]
ATG714	*fqSi141 II; htp-1(gk174) IV*	This study
ATG715	*fqSi181 II; htp-1(gk174) IV*	This study
ATG716	*fqSi167 II; htp-1(gk174)/ nT1[unc-? (n754) let-? qls50] (IV;V)*	This study
ATG540	*htp-1 (syb1225 [htp-2]) IV/ tmC25 [unc-5(tmIs1241)] IV*	This study, *syb1225* allele made by SunyBiotech.
ATG665	*htp-1 (fq162 [htp-2::FLAG]) IV/ tmC25 [unc-5(tmIs1241)] IV*	This study
ATG607	*htp-1 (fq141 [Δhtp-1]) IV/ tmC25 [unc-5(tmIs1241)] IV*	This study
ATG669	*htp-2 (fq163 [Δhtp-2]) IV*	This study
ATG717	*htp-1 (syb4096 [E191A E200K]) IV*	This study, *syb4096* allele made by SunyBiotech.
ATG643	*htp-1 (fq152 [htp-1C239H N243H E245Q::FLAG]) IV/ tmC25 [unc-5(tmIs1241)] IV*	This study
ATG650	*htp-1 (fq154 [htp-1 C239H N243H E245Q M249K L250S::FLAG]) IV/ tmC25 [unc-5(tmIs1241)] IV*	This study
ATG656	*htp-1 (fq157 [htp-1 D226G A230V C239H N243H E245Q M249K and L250S::FLAG])/ tmC25 [unc-5(tmIs1241)] IV*	This study
ATG677	*htp-1 (fq24 [htp-1::FLAG]) IV; htp-2 (fq163 [Δhtp-2]) IV*	This study
ATG491	*htp-1 (fq98 [htp-2 HORMA C E191-L250]) IV/ nT1[unc-? (n754) let-? qls50] (IV;V)*	This study

ATG493	*htp-1 (fq98 [htp-2 HORMA C E191-L250]) IV/ tmC25 [unc-5(tmIs1241)] IV*	This study
ATG565	*htp-1 (fq128 [htp-2 HORMA C E191-L250::FLAG]) IV/ tmC25 [unc-5(tmIs1241)] IV*	This study
ATG588	*htp-1 (fq98 [htp-2 HORMA C E191-L250]) IV; syp-2 (ok307) V/ nT1[unc-? (n754) let-? qIs50] (IV;V)*	This study
ATG582	*htp-1 (syb1225 [htp-2]) IV; syp-2 (ok307)/ nT1[unc-? (n754) let-? qIs50] (IV;V)*	This study
AV402	*htp-1(gk174) IV; syp-2(ok307) V)/ nT1[unc-? (n754) let-? qIs50] (IV;V)*	(Martinez-Perez et al., 2005)[1]
ATG696	*htp-1 (fq178 [htp-1 D144K L156K::FLAG]) IV/ tmC25 [unc-5(tmIs1241)] IV*	This study
ATG542	*htp-1 (fq115 [ΔP151-E162]) IV/ tmC25 [unc-5(tmIs1241)] IV*	This study
ATG543	*htp-1 (fq116 [ΔR140-E162]) IV/ tmC25 [unc-5(tmIs1241)] IV*	This study
ATG670	*htp-1 (fq164 [htp-1 ΔP151-E162::FLAG]) IV/ tmC25 [unc-5(tmIs1241)] IV*	This study
ATG671	*htp-1 (fq165 [htp-1 ΔR140-E162::FLAG]) IV/tmC25 [unc-5(tmIs1241)] IV*	This study
ATG691	*htp-3 (fq175 [ΔS136-V149]) I/ hT2[bli-4(e937) let-?(q782) qIs48] (I;III)*	This study
ATG654	*htp-1 (fq156 [him-3 S344D-K352G::FLAG]) IV/ tmC25 [unc-5(tmIs1241)] IV*	This study
ATG726	*htp-3 (fq185 [A137K]) I/ tmC18 [dpy-5(tmIs1236)] I*	This study
ATG748	*htp-1 (fq192 [htp-1 A139K::FLAG]) IV/ tmc25 [unc-5(tmIs1241)] IV*	This study
ATG777	*htp-1 (fq203 [htp-1 151aa-162aa scramble::FLAG]) IV/ tmc25 [unc-5(tmIs1241)] IV*	This study

Supplementary Table 1. List of strains used in this study.

References

1 Martinez-Perez, E. & Villeneuve, A. M. HTP-1-dependent constraints coordinate homolog pairing and synapsis and promote chiasma formation during C. elegans meiosis. *Genes Dev* **19**, 2727-2743 (2005). https://doi.org:10.1101/gad.1338505

2 Colaiacovo, M. P. *et al.* Synaptonemal complex assembly in C. elegans is dispensable for loading strand-exchange proteins but critical for proper completion of recombination. *Dev Cell* **5**, 463-474 (2003).

3 Sato-Carlton, A. *et al.* Phosphoregulation of HORMA domain protein HIM-3 promotes asymmetric synaptonemal complex disassembly in meiotic prophase in Caenorhabditis elegans. *PLoS Genet* **16**, e1008968 (2020). https://doi.org:10.1371/journal.pgen.1008968

4 Ferrandiz, N. *et al.* Spatiotemporal regulation of Aurora B recruitment ensures release of cohesion during C. elegans oocyte meiosis. *Nat Commun* **9**, 834 (2018). https://doi.org:10.1038/s41467-018-03229-5

Transgene	Genotype
fqSi6	[*Phtp-1 htp-1 wild type 3'UTR htp-1*; *cb-unc-119(+)*]
fqSi22	[*Phtp-1 htp-1::6HIS 3'UTR htp-1*; *cb-unc-119(+)*]
ieSi27	[*Phtp-1 htp-2 N-terminus(1-41aa) htp-1(42-352aa) 3' UTR htp-1; cb-unc-119(+)*]
fqSi28	[*Phtp-1 htp-1 (1-250aa) htp-2 C-terminus (251-352aa) 3' UTR htp-1; cb-unc-119(+)*]
fqSi30	*[Phtp-1 htp-1 (1-41aa) htp-2 HORMA (42-250aa) htp-1(251-352aa) 3' UTR htp-1*; *cb-unc-119(+)*]
fqSi141	[*Phtp-1 htp-1 (1-41aa) htp-2 HORMA A (42-90aa) htp-1 (91-352aa)::6HIS 3'UTR htp-1*; *cb-unc-119(+)*]
fqSi181	*[Phtp-1 htp-1(1-90aa) htp-2 HORMA B (91-165aa) htp-1 (166-352aa)::6HIS 3'UTR htp-1*; *cb-unc-119(+)*]
fqSi167	[*Phtp-1 htp-1 (1-165aa) htp-2 HORMA C (166-250aa) htp-1 (251-352aa)::6HIS 3'UTR htp-1*; *cb-unc-119(+)*]

Supplementary Table 2. Transgenes used in this study.

Allele	gRNA	Donor oligonucleotide for homologous recombination
htp-2::FLAG	GTAGTTGGTC TCATCTTGAT	FLAG sequence CTCGAGGATTACAAGGATGACGACGATAAG flanked by 800bp of *htp-2,* upstream of stop codon, and 800bp of 3’UTR of *htp-2*
***htp-1**$^{htp\text{-}2\ HORMAC}$*	sg1GCCGTGAATTCTTCTGGCAA<br>sg2 CAACTCTGCTTCGTATGAAT	Left oligo: GTGTTCAGTTCCTCTGCACAAAAGTTCTCGAACCGCTACCGGCGGAATTCACGGCTAATTTTCGA<br>TTGAAGTACACAAACGATGCTCCATCCAATTTCCGCATCGATGGCTTCGACGATTCTTCCACTTTTTACACAC<br>TTCCTGATGGTA<br>Right oligo:<br>CGATTCTTCCACTTTTTACACACTTCCTGATGGTATCCAATCGGTCACCATTGGTCACTTACGCCCGGGA<br>CACCACGCTGCACACATGCAATGCTGGAGCAAGTCGATGAGTGACTCATACGAAGCAGAGTTGACTCTCAAGC<br>GACACCT
htp-1[htp-2::FLAG]* <br>*Addition of FLAG tag to *htp-1 (syb1225 [htp-2])*	GCTAGGAAAAAAATTATTTA	AAACCAGATCTTCTCCATACTCTCGCTCCCGTAAACTCGAGGATTACAAGGATGACGACGATAAGTGATAAtttttttc<br>ctagctttcaatagattatagctt
htp-1Δ	sg1TGAGACTGTTTTTTTGCAGA<br>sg2GCTATAATCTATTGAAAGCT	caaactcttgcctaaaaatgagactgtttttttgcTACCCATACGATGTTCCAGATTACGCTtttcaatagattatagcttcattctgtaacttggc
htp-2 Δ	sg1ACAATTTGAAGACATTTTTA<br>sg2TATAGAACATGCCATGTTAC	ttttttttgaattcaaaaattaaactttgccctaaTACCCATACGATGTTCCAGATTACGCTacatggcatgttctatatcatcatgtacaatgttt
htp-1**$^{C239H\ N243H\ E245Q}$ **(htp-1**^{mut3}**)	AGGATGCCACGCTGCAAACA	tccaatcggcaaccattggtcacttgcgcccaggaCATcaTgcCgcGCATatgCAGtgTtggagTatgttgatgtccgattcatacgaagcagagttgac
htp-1**$^{C239H\ N243H\ E245Q\ M249K\ L250S}$ **(htp-1**^{mut5}**)	CAACTCTGCTTCGTATGAAT	ccaggaCATcaTgcCgcGCATatgCAGtgTtggagTaaatcaatgAGTgaCtcGtaTgaagcagagttgactctcaagcgacacctggacaa
htp-1**$^{D226G\ A230V\ C239H\ N243H\ E245Q\ M249K\ L250S}$**(htp-1**^{mut7}**)	ACTTCCTGATGATATCCAAT	cgaagattcttccactttttacacacttcctgatGGAatTcaaAGCGTCaccattggtcacttgcgcccaggaCATcaTgcCgc
***htp-1**^{D144KL156K}*	sg1CGTCTGAGCACTGACAAGAA<br>sg2TGAGCAAGCTTTGCGAAGTG	tcatttattttgagaatggcggcgtggttgctcgtTTAAGCACGAAGAAAAACGGCCAGGAAGATCCGCATTTTGCGAAGAAG<br>gctcaacttgtatacgagggtggcgacagcgtacg
***htp-1**$^{Δ151\text{-}162}$*	sg2GAGCACTGACAAGAATGGCC<br>sg3GCTCAACTTGTATACGAGGG	GGCGGCGTGGTTGCTCGTCTGAGCACTGACAAGAACGGTCAAGAGGACGGTGGCGACAGCGTACGAGATCAAA<br>TGGTGACGAT
***htp-1**$^{Δ140\text{-}162}$*	sg1CTTCATAGAGAAAACTTCGA<br>sg3GCTCAACTTGTATACGAGGG	GATCACAAATAATGAAGGAGATCTTGAAGCCATCGAAGTTTTCTCTATGAAGTTCATTTATTTTGAGAATGGCG<br>GCGTGGTTGCTGGTGGCGACAGCGTACGAGATCAAATGGTGACGAT
htp-1**$^{Δ140\text{-}162}$**::FLAG<br>***htp-1**$^{Δ151\text{-}162}$**::FLAG***	CTAGGAAAAAAATTATTTAC	AAACCAGATCGTCTCCATATTCTCGTTCCTGTAAACTCGAGGATTACAAGGATGACGACGATAAGTGATAAtttttttc<br>ctagctttcaatagattatagctt
***htp-1**$^{HIM\text{-}3\ CM}$*	TATTTACAGGAACGAGAATA	ctactcgcagaggagccgatggcagcaaaaccagaGATTCCCCTTACGGTCTCAGTCAGGGCATTACGAAGAAGAATAAGGACCTC<br>G<br>AGGATTACAAGGATGACGACGATAAGTAAtttttttcctagctttcaatagattatagctt
***htp-3**$^{Δ136\text{-}149}$*	sg1ACATCTGCAAGTGCCGAAAT<br>sg2 CAGGCGTAAATCTAAATTTG	CGTTTGTCTGGACCTTTGTCTATGACTCAACCACATCTGCAGTGAATTATTTAAACATGGATGATACTGC
***htp-3**^{A137K}*	ACATCTGCAAGTGCCGAAAT	GTCTGGACCTTTGTCTATGACTCAACCACATCTGCCTCTAAAGAGATTGGCTATGGAGGgttagatcatttatct
***htp-1**^{A139K}*	CGTCTGAGCACTGACAAGAA	TGAAGTTCATTTATTTTGAGAATGGCGGCGTGGTTAAGCGTTTATCAACGGATAAAAACGGACAGGAAGATCCT<br>CACTTCGCAAAGCTTGCTCAACT
***htp-1**$^{SCR151\text{-}162}$*	AAGAACGGTCAAGAGGACGG	AATGGCGGCGTGGTTGCTCGTCTGAGCACTGACAAAAACGGTCAAGAGGACCAAGTAGAGCACCTTGCTCCTTT<br>CAAGGCACTTTACGGTGGCGACAGCGTACGAGATCAAATGGTGACGAT

Supplementary Table 3. Single guide RNAs and dsDNA repair templates used for CRISPR-Cas9 alleles generated in this study.
