# Supplementary material for: Two structurally mobile regions control the conformation and function of metamorphic meiotic HORMAD proteins
Source: Nat Commun. 2026 May 19;17:6615. doi: 10.1038/s41467-026-72656-6 (PMC13381836; doi:10.1038/s41467-026-72656-6)
Supplement: Supplementary file 3 — Transparent Peer Review file [file 41467_2026_72656_MOESM3_ESM.pdf]

nature portfolio

Peer Review File

Two structurally mobile regions control the conformation and function of metamorphic meiotic HORMAD proteins

Corresponding Author: Dr Enrique Martinez-Perez

This file contains all reviewer reports in order by version, followed by all author rebuttals in order by version.

Attachments originally included by the reviewers as part of their assessment can be found at the end of this file.

Version 0:

Reviewer comments:

Reviewer #1

(Remarks to the Author)
The manuscript by Martinez-Perez and colleagues addresses a fascinating questions relating to the molecular and structural basis of the adaptations required for carrying out meiotic chromosome dynamics. HORMA domain proteins play a part in various chromosomal processes, and in meiosis they mold chromosomes into elongated structures by forming an 'axis', which in turn regulates almost every aspect of meiosis. These functions include interactions of the parental chromosomes with each other, to exchanging of genetic information, and correct segregation into the gametes.

The author provide elegant evidence as to the importance of a specific structural element in meiotic HORMA proteins - the beta5-alphaC loop - in mediating the various functions of metic HORMA proteins. This is achieved by a combination of elegant and thorough genetic domain analysis in C. elegans nematodes, which is complemented by molecular dynamic simulation.

The data is of high quality and is overall convincing. Several key conclusions are overstated, and should be toned down, removed or validated by additional experiments. Due to lack of expertise, I cannot critically analyze the results of the molecular dynamics simulations and other structural modeling, and I leave commenting on them to other, more qualified, reviewers.

Generally speaking, Figures 1-4 of the paper represent thorough genetic analysis of the specific contribution of two similar meiotic HORMA domain proteins in C. elegans, HTP-1 and HTP-2. The data is surprising and unexpected: rather than partial redundancy between these recently duplicated and very similar proteins, the authors demonstrate that these two proteins have distinct meiotic functions, and link them to a small number of a.a. substitutions in one region of the protein. Beyond a few minor issues (noted below), I find the conclusions drawn from this part well supported by the data.

My main concerns lie with the second part of this work, and specifically with how the authors test the models developed based on the simulation and modeling. All the mutants analyzed in Figs. 6 & 7 are characterized by failure to load onto meiotic chromosomes. The authors interpreted this results to reflect the formation of a particular fold that cannot associate with other meiotic HORMAs. However, it remains completely possible that these mutants affects HORMA folding or stability in other ways. Without clear demonstration that these are truly separation-of-function mutations (i.e., that these mutations specifically affects the function of the the beta5-alphaC loop in regulating the closed/open conformation of the HORMA domain), these conclusions need to be dramatically toned down. Obviously, any additional experiments that would address the specificity of these mutations on the structure and function of HTP-1 and HTP-3 will strengthens the conclusions of the manuscript.

Minor issues:
1. One of the hallmark phenotypes of htp-1 null worms (initially defined by the lead author) is extensive, non-homologous synapsis. While this configuration is clearly abnormal and leads to lack of crossovers and extensive gamete aneuploidy, it is distinct from other forms of meiotic dysfunction, such as complete lack of synaptonemal complex assembly. This important difference is mostly lost in much of the quantification and accompanying discussion. For example, the choice to score only

nuclei that are ‘fully synapsed’ (Fig. 3b) masks much of this complexity. This is particularly important considering the wide readership of Nature Communications.The authors should clarify this complexity in the text, in addition to potentially repeating their quantification in a manner that would capture it.

2. The analysis of PLK-2 foci is unclear and over interpreted. First, the images supplied (Fig. 3c & 6e) do not allow to discern what is being quantified. Please replace them with images with different colors or different saturation, and better legends. More importantly, the authors' interpretation of these results (the paragraph on page 11), oversimplify how these foci could be interpreted. While many meiotic mutants exhibit phenotypes that are consistent with the following statement - “mutants with synapsis defects accumulate nuclei with multiple PLK-2 aggregates, while mutants deficient in crossover formation but proficient in synapsis accumulate nuclei with a single PLK-2 aggregate” - the current state of knowledge in the field does not allow to directly deduce these effects based solely on the number of PLK-2 aggregates. I suggest the authors modify the text to soften their conclusions accordingly, or add additional analysis that would allow them to make these conclusions.

Reviewer #2

(Remarks to the Author)
This is an important, rigorous, exceedingly well-executed manuscript that identifies key differences between meiotic HORMADs and other HORMAD proteins to potentially explain the unique behavior of meiotic HORMADs during meiotic prophase. Exploiting the fact that C. elegans have a duplicated, non-essential version of HTP-1, HTP-2, the authors perform a series of creative swapping experiments to test what regions of HTP-1 and HTP-2 explain the meiotic requirement for HTP-1. Using a combination of structural analysis and in vivo testing, the authors change key residues in the safety belt region of HTP-1 to residues found in HTP-2 to demonstrate that safety belt region explains the functional differences between HTP-1 and HTP-2. Next, the authors used equilibrated molecular dynamics (MD) simulations to determine what effect these mutations have on the structure and mobility of portions of HTP-1/HTP-2. These analyses identified another structurally mobile region, the β5-αC loop, that is longer and more structured in meiotic HORMADs than in Mad2 and appears to play a role in stabilizing the closed conformation through an interaction with the safety belt. The authors test the functionality of this loop in HTP-1 and find that deletion of this loop either prevents or destabilizes chromosomal loading, affecting synapsis, crossover formation and feedback mechanisms. Moreover, the function of this loop appears to be functionally conserved in another essential meiotic HORMAD, HTP-3. Since removal of this loop in the budding yeast meiotic HORMAD, Hop1, produces a conformation where the HORMA domain adopts a closed conformation without binding a closure motif, the authors conclude that the defects they observe in these “loopless” mutants are a product of adopting the closed conformation without binding their partners, preventing their chromosomal loading. They test this possibility by installing a closure motif that HTP-1 binds with higher affinity (HIM-3CM) than its own and mimicking the htp-1 “loopless” mutation. They then test the possibility that this loop may further stabilize the closed conformation that meiotic HORMADs adopt when they bind closure motifs on their chromosomal partners by mutating residues identified in their simulations as important for this stabilization. These mutants produce protein that are less stably bound to chromosomes and exhibit defects in synapsis and the activation of feedback mechanisms. Finally, additional simulations with HTP-3 suggested that a version of HTP-3 that is not bound to a protein with a closure motif may also engage this loop, so that it contacts the open HORMA domain. They call this the “loop engaged” conformation and test the functionality of this proposed structure by making a mutation that is predicted to disrupt this interaction and find that HTP-3 fails to load and support synapsis and recombination. Simulations of other meiotic HORMADs in which the safety belt has been deleted show that they also adopt this “loop engaged” conformation, suggesting its general relevance.

The results are largely convincing, clear, appropriately interpreted and mostly communicated with clarity and accessibility. I have a few suggestions to improve the clarity and justify some of the interpretations of the manuscript.

Major
A figure showing the differences between the β5-αC loop in meiotic HORMADs and Mad2 and Rev7 would be useful, given the focus on this region in the study.

Why were the simulations in Figure 4 performed without the closure motifs? How do the authors imagine full length HTP-1 and HTP-2 structures maintain their closed conformations without the closure motifs at this stage of their analysis? This becomes more clear later in the paper but is distracting and unclear in this section.

What are the observed number of DAPI stained bodies in htp-1D144KL156K mutants, given their low level of embryonic lethality?

In general, the manuscript would benefit from more clarity and rationale for experiments and interpretations, especially with the CollabFold simulations. For example, the fact that htp-3A137K mutant phenotypically resembles the loopless htp-3 mutant (htp-3 136-149) could be explicitly highlighted to support the interpretation that the unliganded, closed engaged loop conformation has in vivo relevance before the discussion.

I was confused by the distinction between the closed capped conformation and the loop engaged conformation and concerned that the introduced mutations could be destabilizing both, particularly since all of these mutations are in the loop. Given the novelty of this striking finding, additional experiments would convince me of the strength of this claim. For example, do mutations in htp-1 that mimic htp-3A137K look different than htp-1D144KL156K and do mutations in htp-3 that mimic htp-1D144KL156K look different than htp-3A137K?

The stability and expression levels of mHORMAD mutants (htp-1 140-162, htp-1 151-162, htp-3 136-149, htp-1D144KL156K, htp-1HIM-3CM, and htp-3A137K) that fail to load or exhibit reduced loading on meiotic chromosomes should be verified by Western.

Minor:
Reference 32 should be updated to the peer reviewed paper published in Nature Communications

Reviewer #3

(Remarks to the Author)
Meiotic recombination is initiated, and regulated in part through the activity of meiotic HORMA domain proteins (mHORMADs). The HORMA domain is a metamorphic protein, capable of adopting at least two topological conformations that facilitate downstream signalling. In the majority of eukaryotes studied there appears to be a single mHORMAD, but some organisms have two (e.g. mammals) and nematodes have up to four mHORMADs.

Barroso show that, despite the high sequence similarity, the C.elgans mHORMADs HTP-1 and HTP-2 are not functionally redundant and that HTP-2 cannot replace the functionality of HTP-1 (htp-2Δ has no clear phenotype). Through rigorous mutational analysis the authors find that the difference between HTP-1 and HTP-2 lie in 7 amino acids within the HORMA domain of the protein. Using structural predictions combined with molecular dynamics the authors propose that the extended beta5-alphaC loop plays an important role in the conformation of the mHORMAS, ultimately broadening the range of downstream signalling that can be performed by mHORMADs.

Barroso et al. is of significant potential importance not only in the field of meiotic recombination, but also in the study of other HORMA domain containing systems and metamorphic signalling proteins in general. The in vivo studies are rigorous and well carried out. The manuscript is generally very well written, though the authors would be advised to make further use of cartoons and annotations throughout the figures to provide further clarity on the experimental data that is being presented.

My main concern is that the model for different conformations of the HORMA domain, as presented by the authors, is lacking in direct experimental evidence. In particular, studies on recombinant HTP-1 would be appropriate here. While the ideal experimental approach would utilise time-resolved NMR (as recently used by the Barford group for Mad2), I understand that this is likely unrealistic. I would be supportive of any in vitro approach that directly tested the model the authors propose. Alternatively, the authors should tone-down their claims, and present alternative models that would explain the phenotypes observed for the HTP-1 mutants.

Minor points
An introductory panel(s) for figure 1 would help the reader better understand the questions the authors are attempting to address
In Figure 2b the alphaA helix is coloured solid red in the sequence alignment, and partial red in the model. Also a different red tone would be welcome in the model, to match to sequence alignment colouring.
Figure 3d and 3e - Despite observing a larger zone of DSB-2 activity in the htp-1 htp-2HORMAC mutant, fewer RAD-51 foci were observed. This should be clearly explained in the corresponding results section.
Figure 4a - the amino acid substitutions used should be made more clear
Again, the authors should be consistent with the colouring. In figures 4 and 5 the safety belt is coloured in tan, whereas in figure 7 the safety belt is blue and the closure motifs are orange/tan

Reviewer #4

(Remarks to the Author)
In the manuscript, Barroso et al. investigated meiotic HORMAD proteins of C. elegans (htp-1, htp-2, and htp-3), suggesting a related but a specific bimodal functionality of these proteins. I have focused my review especially on the in silico part of the study. The authors have utilized MD simulations to study one of the states of these meiotic proteins, as well as used ColabFold structure predictions. The manuscript is interesting, but I have some concerns especially related to linking the information obtained from the simulations to the experiments. Also, the simulation data appears somewhat thin to draw the conclusions presented in the manuscript.

1) There seems to be a quite extensive gap between the conducted experiments (in vivo) and the MD simulations presented in the manuscript. The authors should provide at least some biophysical experimental evidence to support the proposed findings from the simulations.

2) There is variability among the simulation replicas; for instance, in specific regions in the RMSF plots. Based on this, 3 replicas seem insufficient to describe the system behaviour to be compared to other systems. Following the general recommendations 5-10 replicas for each system is clearly needed and would justify and solidify the findings/suggestions derived from the simulations.

3) It seems a bit questionable, if the HTP-1Mut7 simulations are stabilized within the simulation timeframe based on their RMSDs. Thus, it is doubtful if there is enough time for the proper system relaxation with the modified system to describe its biologically relevant configuration / conformational ensembles. Moreover, based on the analysis there is a large discrepancy

in the behaviour of one of the replicas compared to the other (heavily related to the point 2). This clearly indicates the need for more replicas and extended simulations. I would also suggest cutting the “equilibrium time” in the beginning (needed for systems to equilibrate) out from the analysis.

4) The authors should stress-test their ColabFold predictions, especially that of htp-3, how stable they are by MD simulations, as they are already studying other systems with this method. This could also provide additional interesting insights.

5) AlphaFold predictions with the full sequence predict an additional helix located with very high confidence on top of the htp-1 (AF-Q20305-F1) and htp-2 (AF-Q95XC8-F1) that could and would have a major impact on the authors predictions related to the loop movements. This would be highly conflicting the proposed model, and the authors should reflect/justify their results related to these predictions.

6) The authors note the role of PTMs in the discussion. The authors should clarify the relevance of the studied state without any PTMs, as now all the modelling (MDs and ColabFold) assume only unmodified proteins. What is the evidence that this state is preferred without any PTMs? How accessible the potential PTM sites are for modifications from the proposed configuration?

7) The authors point out the potential important role of histidine residues in the conformational behaviour with selected proteins. However, no information is given how the tautomeric / protonation states of the histidines are modelled into the structures. Is there some bias to the results from this and would the histidine states explain some of the behaviour?

Other points

1) Please provide the missing information related to the size of the simulation box.

2) “locally written code” It is totally unclear what is this code and to which analysis it refers to?

3) PCA analysis is insufficiently described. How was the PCA conducted? What are the contributions of PC1s and PC2s for each system? Which software/code was used to generate the Fig 5c images? Please label the most important structural elements also in the Fig 5c to improve its readability.

4) The authors do not state how the simulation data is made readily accessible for the readers. Please provide the simulation data available, e.g. into a selected repository.

5) Figure 2b, the sequences are not properly aligned, some letters are jumping in the rows. Please improve the figure and its readability.

6) Figure 5e: surface representation is unclear. This should be improved. I suggest displaying this surface with a single colour, and to display the surface of the rest of the protein (light grey) to provide improved presentation for the reader about the solvent accessibility.

7) Typos in fig 5 text: “Solvent assessable”; Protein structure prediction methods: “Supposition”; “PyMOL vX”

Version 1:

Reviewer comments:

Reviewer #1

(Remarks to the Author)
The additional experiments and changes to the manuscript address my main concerns. I would like to commend the authors on a much improved manuscript.

Below I note several minor comments and suggestions that could strengthen the manuscript and make it more accessible for a wide readership.

In figure 4a, I would recommend adding a.a. numbers for all the mismatched positions (and potentially for the whole range), given that they are referred to in the text.
I couldn’t find evidence or data supporting this statement: “A mutant strain carrying the E191A and E200K substitutions did not show obvious meiotic defects” on page 13. I recommend adding data or rephrasing.
On page 18, there’s a reference for Fig. 7b-e that should be for Fig. 7b-d.
Fig. 7g: I realize the trickiness of quantifying western blots. Given this, I recommend adding at least one additional example of this blot to demonstrate that the result is reproducible.
Fig. 9: I found the model, and esp. the graphical language used throughout, very helpful. I do however, suggest linking some of the in vivo phenotypes (or suggested outcomes) to the graphics. For example, adding to the figure which of the conformation the authors presume is competent for binding to the axis and which are prone to degradation would be quite helpful. In addition, referring to the labels in the text (e.g., “C-HORMAD”, etc.) would improve readability.

Fig. 5C-D: Any graphical indication of what exactly is quantified in the graphs would be helpful. In C, I *think* that the average of the value in the y-axis is quantified, but I'm not actually sure. This is especially important since, for example, it is the variability for mut7 that is the most striking difference. So I would suggest focusing the reader's attention on the relevant difference.
Throughout, the authors switch between discussing parts of the proteins based on the numbering system of the alpha helices and beta sheets, and the functional names ('safety belts'). The labeling of the figures is also not always consistent (e.g., Fig. 2b, 4a and 5a). Unifying this across the text and figures will improve the readability of the manuscript.

Reviewer #2

(Remarks to the Author)
The authors have addressed my experimental concerns. I particularly appreciated the analysis of the htp-1SCR 151-162 mutant, which was a particularly clever addition to the manuscript.

I have two additional suggestions to help with their Discussion, which nicely summarizes the experimental results but could address some caveats more concretely:

1. Can the authors provide information about which conformations in Figure 9 are stable (expressed at levels comparable with wildtype) or unstable and which are competent and incompetent for axis loading?

2. A major point of the paper seems to be that some mutants destabilize the protein, reducing protein levels, but not abrogating them, both in vivo and in vitro , either explaining or contributing to the axis loading defects they observe. The authors argue that since the htp-1 HIM-3CM mutant fails to bind meiotic chromosomes but is expressed at levels comparable to wildtype HTP-1, this demonstrates that loading onto meiotic chromosomes does not affect protein stability per se. While that is definitely one compelling argument, I think another compelling argument that these mutations are functionally relevant is that, critically, these mutants do not produce the same phenotype as the deletion of the htp-1 gene. These data demonstrate that even at reduced protein levels, these mutations produce striking effects on meiotic progression, pairing, synapsis and recombination distinct from complete loss of the protein, arguing against the possibility that the observed phenotypes are because these mutations destabilize the protein.

I'm concerned that this point is not made as plainly as it could, potentially obscuring the important results of the detailed biochemical and mutational analysis. I recommend that the authors make this point more clearly, earlier in the discussion. This will ensure that the rest of the discussion, with its compelling argument about a more expansive set of conformations associated with meiotic HORMADs and the consequences for function, is appreciated as much as it should be.

Reviewer #3

(Remarks to the Author)
The authors have satisfied my concerns, and really gone above and beyond in a very rigorous response. I recommend acceptance of this excellent, fascinating, and important manuscript.

Reviewer #4

(Remarks to the Author)
I thank the authors for revising the manuscript and addressing my comments. I am satisfied with their responses and modifications. As a side note, it is rather interesting to see how the pIDDTs seem to correlate with RMSFs (Figs S5a&b). I have listed below some minor points and errors that could be fixed to polish the manuscript.

Please use consistent colors for the highlighted structural regions throughout the manuscript. It is difficult to read while this change all the time from figure to figure and panel to panel. (This applies to the 3D-structures and how those regions are highlighted in the plots)

Fig 5B) It is unclear which replica is shown in the figure, please specify.

Fig 5C) I would suggest to either show the 3D structure in an orientation as it is in the other panels and/or highlight the β5-αC loop and the safety belt regions as in 5a.

Fig. S5a) Wrong colors in the figure text: "indicating the β5-αC loop in orange and the safety belt region in blue.".
Please highlight the β5-αC loop and the safety belt region also in the pIDDT plots.

Fig S5b) Please use consistent colors for the highlighted regions here with the presented 3D-structures to improve clarity and readability.

Fig S5d) Why the color of HTP-1mut7 position 1 in the left panel is not orange as indicated in the figure text?

Figure S6b) This figure is not necessary and can be omitted (as you provide the information in methods), and it allows you to fit the S6 in one page.

The authors write in p.33 “…the preferred tautomeric form of histidine in proteins at physiological pH” – I would argue that this is highly dependent on the environment and the preference is higher the more solvent exposed histidine is (also in the cited paper it is pretty evident that not all His will be in this form). But feel free to keep the text as it is if you wish.

Please add the version numbers for VMD and PyMOL and properly cite PyMOL.
(e.g. The PyMOL Molecular Graphics System, Version 3.0 Schrödinger, LLC.)

There are also still two typos of "PyMOL vX” in the methods.

Open Access This Peer Review File is licensed under a Creative Commons Attribution 4.0 International License, which permits use, sharing, adaptation, distribution and reproduction in any medium or format, as long as you give appropriate credit to the original author(s) and the source, provide a link to the Creative Commons license, and indicate if changes were made.
In cases where reviewers are anonymous, credit should be given to 'Anonymous Referee' and the source.
The images or other third party material in this Peer Review File are included in the article’s Creative Commons license, unless indicated otherwise in a credit line to the material. If material is not included in the article’s Creative Commons license and your intended use is not permitted by statutory regulation or exceeds the permitted use, you will need to obtain permission directly from the copyright holder.
To view a copy of this license, visit https://creativecommons.org/licenses/by/4.0/

We thank the reviewers for their overall support and constructive criticism of our manuscript. Below, we first provide a summary of the main additions that we have introduced in our revised manuscript in response to all reviewers' comments before addressing comments from each reviewer individually and point by point.

The main additions in this revised version are:

1- We have added in vitro experiments to evaluate how different HTP-1 mutants bind to a closure motif (CM) sequence from HIM-3. These experiments demonstrate that the β5-αC loop of HTP-1 is required for CM binding and that the ability to bind a CM is essential for protein stability (even for WT HTP-1). These experiments include HTP-1 mutants reported in the original version, plus additional mutants added in response to specific reviewer's comments to further dissect how specific regions/residues of the β5-αC contribute to the proposed mHORMAD conformations (see below).
2- We have created a new HTP-1 mutant, HTP-1^{A139K} (corresponding to HTP-3^{A137K}) that has confirmed that this single amino acid substitution predicted to hinder the interaction between the β5-αC loop and the HORMA core prevents axis loading in vivo as well as HIM-3 CM binding in vitro.
3- We have also created a new HTP-1 mutant (HTP-1$^{SCR\ 151\text{-}162}$) in which residues 151-162 of the β5-αC loop, which are not predicted to interact directly with the HORMA core in the loop engaged conformation, are replaced by a scrambled sequence in which each of the 12 amino acids is replaced by a different one. In contrast to HTP-1^{A139K} mutants, analysis of HTP-1$^{SCR\ 151\text{-}162}$ mutants demonstrates normal loading to axial elements and normal chiasma formation in vivo, as well as normal binding to HIM-3 CM in vitro, despite the 12 amino acid substitutions. These findings support the role of this flexible region as a spacer sequence that allows the N-terminal region of the β5-αC loop, which contains A139, to move downwards to interact with residues in the HORMA core, as proposed in the loop engaged conformation.
4- We have expanded our MD modelling experiments by increasing the number of simulations from 3 to 5 and by extending each simulation from 500 ns to 1000 ns. These expanded simulations support the dynamic behaviour of the β5-αC loop and its ability to move downwards towards the HORMA core.
5- We have extensively rewritten the discussion to better highlight our key findings and acknowledge limitations. We now provide comparison between our model and known Mad2 conformations in which a mobile, and structurally dynamic, region at the N-terminus competes with the safety belt for binding to β5 on the HORMA core to control Mad2 conformation. This provides a clear analogy for the proposed competition between the safety belt and the flexible β5-αC loop for binding β5 in mHORMADs.

Reviewer #1 (Remarks to the Author):

The manuscript by Martinez-Perez and colleagues addresses a fascinating questions relating to the molecular and structural basis of the adaptations required for carrying out meiotic chromosome dynamics. HORMA domain proteins play a part in various chromosomal processes, and in meiosis they mold chromosomes into elongated structures by forming an 'axis', which in turn regulates almost every aspect of meiosis. These functions include interactions of the parental chromosomes with each other, to exchanging of genetic information, and correct segregation into the gametes.

The author provide elegant evidence as to the importance of a specific structural element in meiotic HORMA proteins - the beta5-alphaC loop - in mediating the various functions of metic HORMA proteins. This is achieved by a combination of elegant and thorough genetic domain analysis in C. elegans nematodes, which is complemented by molecular dynamic simulation.

The data is of high quality and is overall convincing. Several key conclusions are overstated, and should be toned down, removed or validated by additional experiments. Due to lack of expertise, I cannot critically analyze the results of the molecular dynamics simulations and other structural modeling, and I leave commenting on them to other, more qualified, reviewers.

We thank the reviewer for their overall positive evaluation of the manuscript. As detailed below, we have added additional experiments to support some of the key points of the manuscript, in particular the importance of the β5-αC loop for the function of HTP-1 and HTP-3.

Generally speaking, Figures 1-4 of the paper represent thorough genetic analysis of the specific contribution of two similar meiotic HORMA domain proteins in C. elegans, HTP-1 and HTP-2. The data is surprising and unexpected: rather than partial redundancy between these recently duplicated and very similar proteins, the authors demonstrate that these two proteins have distinct meiotic functions, and link them to a small number of a.a. substitutions in one region of the protein. Beyond a few minor issues (noted below), I find the conclusions drawn from this part well supported by the data.

My main concerns lie with the second part of this work, and specifically with how the authors test the models developed based on the simulation and modeling. All the mutants analyzed in Figs. 6 & 7 are characterized by failure to load onto meiotic chromosomes. The authors interpreted this results to reflect the formation of a

particular fold that cannot associate with other meiotic HORMAs. However, it remains completely possible that these mutants affects HORMA folding or stability in other ways. Without clear demonstration that these are truly separation-of-function mutations (i.e., that these mutations specifically affects the function of the the beta5-alphaC loop in regulating the closed/open conformation of the HORMA domain), these conclusions need to be dramatically toned down. Obviously, any additional experiments that would address the specificity of these mutations on the structure and function of HTP-1 and HTP-3 will strengthens the conclusions of the manuscript.

We have performed additional experiments to provide further support for our proposal that the interaction of the β5-αC loop with the HORMA core, particularly with β5 to form the loop-engaged conformation, is an important aspect of mHORMAD function. These experiments, detailed below, include both in vivo and in vitro assays to test the function of different HTP-1 mutants.

First, we have added in vitro experiments to evaluate how different HTP-1 mutants bind to a closure motif (CM) sequence from HIM-3 by co-expressing HTP-1 and the HIM-3 CM linked to 6His-MBP in bacteria (Figures 7 e-f and 8o). These experiments demonstrate that the β5-αC loop of HTP-1 is required for closure motif binding and that the ability of HTP-1 to bind a closure motif is essential for protein stability. This last point is demonstrated by the reduced levels of HTP-1 protein when WT HTP-1 is co-expressed with a mutant version of HIM-3's CM that can't be bound by HTP-1. Conversely, expression of HTP-1 carrying HIM-3's CM at its C-terminus results in very stable protein despite failure in binding to the external HIM-3 CM linked to MBP. The effect of CM binding on protein stability in vitro is supported by new experiments monitoring HTP-1 and HTP-3 protein levels in vivo (whole worm protein extracts followed by western blot analysis). These experiments demonstrate that protein levels of HTP-1 and HTP-3 loop deletion mutants are severely reduced compared to WT HTP-1 or WT HTP-3 (Figures 7g and 8g). However, levels of HTP-1 carrying HIM-3's CM at its C-terminus are similar to WT HTP-1 levels (Figure 7g), despite failing to load on axial elements.

Second, we have created a new HTP-1 mutant, HTP-1^{A139K} (corresponding to HTP-3^{A137K}) that has confirmed that this single amino acid substitution, which is predicted to hinder the interaction between the β5-αC loop and the HORMA core of HTP-1, prevents axis loading in vivo and HIM-3 CM binding in vitro (Figures 7g and 8i-j). Moreover, this single amino acid substitution reduces in vivo and in vitro protein levels in a manner analogous to mutants carrying loop deletions (Figures 7g and 8k). These findings demonstrate that the role of A137 (A139 in HTP-1), located in the region of the β5-αC loop predicted to form a β strand that pairs with β5 in the loop engaged conformation, in ensuring axis loading is conserved between HTP-1 and HTP-3.

Third, we have also created a new HTP-1 mutant (HTP-$1^{SCR\ 151\text{-}162}$) in which residues 151-162 of the β5-αC loop (same residues that were deleted in the short loop mutant already reported in the first version of our manuscript) were replaced by a scrambled sequence in which each of the 12 amino acids is replaced by a different one (Figure 8l). In contrast to the region containing A139, this region of the loop is not predicted to form secondary structure or interact with the HORMA core in the loop engaged conformation. Analysis of HTP-$1^{SCR\ 151\text{-}162}$ mutants demonstrates normal loading to axial elements and normal chiasma formation in vivo, as well as normal binding to HIM-3 CM and protein stability in vitro (Figures 8m-o). All these despite the 12 amino acid substitutions and in clear contrast to the strong in vivo and vitro defects displayed by both HTP-1^{A139K} and by HTP-$1^{\Delta 151\text{-}162}$. These findings support that residues 151 to 162 constitute a flexible region that acts as a spacer sequence to allow the N-terminal region of the β5-αC containing A139 to interact with residues in the HORMA core, as proposed in the loop engaged conformation.

We have rewritten sections of the discussion to more clearly correlate our findings to previous research in Mad2 showing that a flexible region at the N-terminus competes with the safety belt for binding β5 in the HORMA core in a manner that determines Mad2 conformation. We also clearly state that further biophysical and structural studies will be needed to fully understand how the β5-αC loop interacts with the HORMA core to control the folding and function of mHORMADs.

Minor issues:
1. One of the hallmark phenotypes of htp-1 null worms (initially defined by the lead author) is extensive, non-homologous synapsis. While this configuration is clearly abnormal and leads to lack of crossovers and extensive gamete aneuploidy, it is distinct from other forms of meiotic dysfunction, such as complete lack of synaptonemal complex assembly. This important difference is mostly lost in much of the quantification and accompanying discussion. For example, the choice to score only nuclei that are 'fully synapsed' (Fig. 3b) masks much of this complexity. This is particularly important considering the wide readership of Nature Communications.The authors should clarify this complexity in the text, in addition to potentially repeating their quantification in a manner that would capture it.

As requested by the reviewer, we have performed FISH together with immunostaining of the SC component SYP-1 to monitor the presence of non-homologous synapsis in *htp-1*$^{htp\text{-}2HORMAC}$. Quantitative analysis of this phenotype confirms that *htp-1*$^{htp\text{-}2HORMAC}$ mutants display extensive non-homologous synapsis. This data is now shows in Figure S3.

2. The analysis of PLK-2 foci is unclear and over interpreted. First, the images supplied (Fig. 3c & 6e) do not

allow to discern what is being quantified. Please replace them with images with different colors or different saturation, and better legends. More importantly, the authors' interpretation of these results (the paragraph on page 11), oversimplify how these foci could be interpreted. While many meiotic mutants exhibit phenotypes that are consistent with the following statement - “mutants with synapsis defects accumulate nuclei with multiple PLK-2 aggregates, while mutants deficient in crossover formation but proficient in synapsis accumulate nuclei with a single PLK-2 aggregate” - the current state of knowledge in the field does not allow to directly deduce these effects based solely on the number of PLK-2 aggregates. I suggest the authors modify the text to soften their conclusions accordingly, or add additional analysis that would allow them to make these conclusions.

We now show high magnification images of single nuclei as examples of the different categories that we have quantified based in the number of PLK-2 aggregates in the nuclear envelope and organisation of chromosomes inside the nucleus (Figure 3c). We have also modified the text to clarify how different markers, including number of PLK-2 aggregates, nuclear morphology, and the presence of the DSB-promoting protein DSB-2, are used to determine the status of meiotic progression.

Reviewer #2 (Remarks to the Author):

This is an important, rigorous, exceedingly well-executed manuscript that identifies key differences between meiotic HORMADs and other HORMAD proteins to potentially explain the unique behavior of meiotic HORMADs during meiotic prophase. Exploiting the fact that C. elegans have a duplicated, non-essential version of HTP-1, HTP-2, the authors perform a series of creative swapping experiments to test what regions of HTP-1 and HTP-2 explain the meiotic requirement for HTP-1. Using a combination of structural analysis and in vivo testing, the authors change key residues in the safety belt region of HTP-1 to residues found in HTP-2 to demonstrate that safety belt region explains the functional differences between HTP-1 and HTP-2. Next, the authors used equilibrated molecular dynamics (MD) simulations to determine what effect these mutations have on the structure and mobility of portions of HTP-1/HTP-2. These analyses identified another structurally mobile region, the β5-αC loop, that is longer and more structured in meiotic HORMADs than in Mad2 and appears to play a role in stabilizing the closed conformation through an interaction with the safety belt. The authors test the functionality of this loop in HTP-1 and find that deletion of this loop either prevents or destabilizes chromosomal loading, affecting synapsis, crossover formation and feedback mechanisms. Moreover, the function of this loop appears to be functionally conserved in another essential meiotic HORMAD, HTP-3. Since removal of this loop in the budding yeast meiotic HORMAD, Hop1, produces a conformation where the HORMA domain adopts a closed conformation without binding a closure motif, the authors conclude that the defects they observe in these “loopless” mutants are a product of adopting the closed conformation without binding their partners, preventing their chromosomal loading. They test this possibility by installing a closure motif that HTP-1 binds with higher affinity (HIM-3CM) than its own and mimicking the htp-1 “loopless” mutation. They then test the possibility that this loop may further stabilize the closed conformation that meiotic HORMADs adopt when they bind closure motifs on their chromosomal partners by mutating residues identified in their simulations as important for this stabilization. These mutants produce protein that are less stably bound to chromosomes and exhibit defects in synapsis and the activation of feedback mechanisms. Finally, additional simulations with HTP-3 suggested that a version of HTP-3 that is not bound to a protein with a closure motif may also engage this loop, so that it contacts the open HORMA domain. They call this the “loop engaged” conformation and test the functionality of this proposed structure by making a mutation that is predicted to disrupt this interaction and find that HTP-3 fails to load and support synapsis and recombination. Simulations of other meiotic HORMADs in which the safety belt has been deleted show that they also adopt this “loop engaged” conformation, suggesting its general relevance.

The results are largely convincing, clear, appropriately interpreted and mostly communicated with clarity and accessibility. I have a few suggestions to improve the clarity and justify some of the interpretations of the manuscript.

Major
A figure showing the differences between the β5-αC loop in meiotic HORMADs and Mad2 and Rev7 would be useful, given the focus on this region in the study.

We have added a new supplemental figure (Figure S1) comparing the β5-αC loop of HTP-1 (used as a mHORMAD example) Mad2, and Rev7. The figure also includes the β5 and αC regions of this proteins to make the comparison of the loop clearer.

Why were the simulations in Figure 4 performed without the closure motifs? How do the authors imagine full length HTP-1 and HTP-2 structures maintain their closed conformations without the closure motifs at this stage of their analysis? This becomes more clear later in the paper but is distracting and unclear in this section.

The MD simulations were performed without closure motifs as ColabFold predictions of HTP-1 and HTP-2 (as well as other mHORMADs) consistently result in a closed conformation despite the absence of a closure motif (we refer to this conformation as empty closed) (Figures S5a and S9a-d). We now make this point clearer by making references to HTP-1 and HTP-2 structural predictions at the start of the MD results section. The

existence of the empty closed conformation is also supported by the fact that Mad2, Rev7, and Hop1can all be purified as empty closed monomers.

What are the observed number of DAPI stained bodies in htp-1D144KL156K mutants, given their low level of embryonic lethality?

Most diakinesis oocytes of htp-1 D144K L156K mutants have 6 bivalents in diakinesis. Quantification of this phenotype is now included as Figure S7f. We have also included this mutant in our new experiments determining HTP-1 protein levels in whole-worm extracts and have observed that protein levels of HTP-$1^{D144K\ L156K}$ are reduced compared to WT HTP-1 (Figure 7g). This observation, together with the reduced loading of HTP-$1^{D144K\ L156K}$ to axial elements reported in the original version of our manuscript support our finding that reduced ability to bind CMs (required for axis loading) reduces HTP-1 protein levels. Interestingly, the pool of HTP-$1^{D144K\ L156K}$ bound to axial elements must retain HTP-1 functionality given that *htp-1$^{D144K\ L156K}$* mutants are largely successful in chiasma formation.

In general, the manuscript would benefit from more clarity and rationale for experiments and interpretations, especially with the CollabFold simulations. For example, the fact that htp-3A137K mutant phenotypically resembles the loopless htp-3 mutant (htp-3Δ136-149) could be explicitly highlighted to support the interpretation that the unliganded, closed engaged loop conformation has in vivo relevance before the discussion.

We have revised the text accordingly to better explain the rationale for creating specific mutants and by highlighting the key conclusions derived from their analysis.

I was confused by the distinction between the closed capped conformation and the loop engaged conformation and concerned that the introduced mutations could be destabilizing both, particularly since all of these mutations are in the loop. Given the novelty of this striking finding, additional experiments would convince me of the strength of this claim. For example, do mutations in htp-1 that mimic htp-3A137K look different than htp-1D144KL156K and do mutations in htp-3 that mimic htp-1D144KL156K look different than htp-3A137K?

Following the suggestion by the reviewer we have created the HTP-1^{A139K} mutant mimicking the HTP-3^{A137K} mutant reported in the original version. This new mutant was analysed in vivo as well as by in vitro experiments designed to evaluate how different HTP-1 mutants bind to a closure motif (CM) sequence from HIM-3 by co-expressing HTP-1 and the HIM-3 CM linked to 6His-MBP in bacteria.

In vivo analysis of HTP-1^{A139K} shows that, as observed for HTP-3^{A137K}, the single amino acid substitution (A to K), which is predicted to hinder the interaction between the β5-αC loop and the HORMA core of HTP-1, prevents axis loading in vivo, causing a failure in chiasma formation (Figures 8i-j). This phenotype is much more severe than the reduced axis loading observed for HTP-$1^{D144KL156K}$. Moreover, HTP-1^{A139K} displays severely reduced binding to HIM-3 CM in our in vitro assay, displaying levels of binding similar to those observed for HTP-1 loop deletion mutants (Figure 7f). Also similar to loop deletion mutants, HTP-1^{A139K} protein levels are reduced in vivo and in vitro (Figures 7f and 8k), presumably due to the inability of HTP-1^{A139K} in acquiring a conformation competent in CM binding (see below). These findings demonstrate that the role in ensuring axis loading of A137 (A139 in HTP-1), located in the region of the β5-αC loop predicted to form a β strand that pairs with β5 in the loop engaged conformation, is conserved between HTP-1 and HTP-3.

Aiming to further investigate how different loop regions contribute to the activity of HTP-1, we have also created a new HTP-1 mutant (HTP-$1^{SCR\ 151\text{-}162}$) in which residues 151-162 of the β5-αC loop (same residues that were deleted in the short loop mutant already reported in the first version of our manuscript) were replaced by a scrambled sequence. Thus, in HTP-$1^{SCR\ 151\text{-}162}$ each one of the 12 amino acids between P151 and E162 is replaced by a different one. In contrast to the region containing A139, the P151-E162 region of the loop is not predicted to form secondary structure or interact with the HORMA core in the predictions showing the loop engaged conformation. Analysis of HTP-$1^{SCR\ 151\text{-}162}$ mutants demonstrates normal loading to axial elements and normal chiasma formation in vivo, as well as normal binding to HIM-3 CM in vitro (Figures 8i-o). All these, despite the 12 amino acid substitutions and in clear contrast to the strong in vivo and vitro defects displayed by both HTP-1^{A139K} carrying a single mutation and by HTP-$1^{\Delta 151\text{-}162}$ in which the 12 amino acids swapped in HTP-$1^{SCR\ 151\text{-}162}$ are deleted. These findings support that residues 151 to 162 constitute a flexible region that acts as a spacer sequence to allow the N-terminal region of the β5-αC loop, which contains A139, to interact with residues in the HORMA core, as proposed in the loop engaged conformation.

We have rewritten sections of the discussion to more clearly correlate our findings to previous research in Mad2 showing that a flexible region at the N-terminus competes with the safety belt for binding β5 in the HORMA core in a manner that determines Mad2 conformation. We also clearly state that further biophysical and structural studies will be needed to fully understand how the β5-αC loop interacts with the HORMA core to control the folding and function of mHORMADs.

The stability and expression levels of mHORMAD mutants (htp-1 Δ140-162, htp-1 Δ151-162, htp-3 Δ136-149,

htp-1D144KL156K, htp-1HIM-3CM, and htp-3A137K) that fail to load or exhibit reduced loading on meiotic chromosomes should be verified by Western.

We have addressed the stability of the HTP-1 and HTP-3 loop mutants in vivo as suggested by the reviewer and expanded this analysis to using our in vitro system described above. In vivo, all *htp-1* and *htp-3* loop mutants tested (*htp-1*$^{\Delta 140\text{-}162}$, *htp-1*$^{\Delta 151\text{-}162}$, *htp-1*^{D144KL156K}, *htp-1*^{A139K}, *htp-3*$^{\Delta 136\text{-}149}$, *and htp-3*^{A137K}) display a clear reduction in protein levels compared to WT HTP-1 or WT HTP-3 (Figures 7g and 8k). This could suggest that inability to bind axial elements is due to protein instability caused by the loop mutations or that HTP-1 and HTP-3 that don't associate with axial elements become unstable. However, two observations argue against these possibilities and instead suggest that inability to bind to a closure motif is the primary defect that leads to protein instability. First, in vivo levels of HTP-1 in *htp-1*$^{HIM\text{-}3\ CM}$ are similar to WT controls (Figure 7g), despite complete failure to bind axial to elements. Thus, failure in loading axial elements per se is not enough to induce HTP-1 instability. In our in vitro system the loop mutants mentioned above (co-expressed with the HIM-3 CM linked to MBP) displayed a clear reduction in total HTP-1 levels, as well as an inability to bind the MBP-bound HIM-3 CM (Figure 7f). However, this was also the case when WT HTP-1 was co-expressed with a mutant version of HIM-3's CM that can't be bound by HTP-1. Conversely, levels of HTP-1$^{HIM\text{-}3\ CM}$ are comparable to WT controls, despite its failure in binding to the external HIM-3 CM linked to MBP. Our in vitro experiments also revealed that overall levels of HTP-1 in total soluble protein extracts were very similar to the amount of HTP-1 detected following MBP-HIM-3 CM pulldown for all HTP-1 mutants tested, except for HTP-1$^{HIM\text{-}3\ CM}$ in which soluble HTP-1 levels were much higher than pulldown levels. This suggests that most HTP-1 in the soluble extracts is already bound to the HIM-3 CM, either in trans (MBP-HIM-3 CM) or in cis (HTP-1$^{HIM\text{-}3\ CM}$). These observations, show that HTP-1 protein that can't bind a CM becomes unstable in vivo and in vitro and that binding a CM is sufficient to stabilise HTP-1 independently of axis binding. They also suggest that the β5-αC loop and its ability to interact with the HORMA core (suggested by analysis of *htp-1*^{A139K} and *htp-3*^{A137K}) are required for the acquisition of a conformation competent in CM binding.

Minor:
Reference 32 should be updated to the peer reviewed paper published in Nature Communications

We have updated this reference.

Reviewer #3 (Remarks to the Author):

Meiotic recombination is initiated, and regulated in part through the activity of meiotic HORMA domain proteins (mHORMADs). The HORMA domain is a metamorphic protein, capable of adopting at least two topological conformations that facilitate downstream signalling. In the majority of eukaryotes studied there appears to be a single mHORMAD, but some organisms have two (e.g. mammals) and nematodes have up to four mHORMADs.

Barroso show that, despite the high sequence similarity, the C.elgans mHORMADs HTP-1 and HTP-2 are not functionally redundant and that HTP-2 cannot replace the functionality of HTP-1 (htp-2Δ has no clear phenotype). Through rigorous mutational analysis the authors find that the difference between HTP-1 and HTP-2 lie in 7 amino acids within the HORMA domain of the protein. Using structural predictions combined with molecular dynamics the authors propose that the extended beta5-alphaC loop plays an important role in the conformation of the mHORMAS, ultimately broadening the range of downstream signalling that can be performed by mHORMADs.

Barroso et al. is of significant potential importance not only in the field of meiotic recombination, but also in the study of other HORMA domain containing systems and metamorphic signalling proteins in general. The in vivo studies are rigorous and well carried out. The manuscript is generally very well written, though the authors would be advised to make further use of cartoons and annotations throughout the figures to provide further clarity on the experimental data that is being presented.

My main concern is that the model for different conformations of the HORMA domain, as presented by the authors, is lacking in direct experimental evidence. In particular, studies on recombinant HTP-1 would be appropriate here. While the ideal experimental approach would utilise time-resolved NMR (as recently used by the Barford group for Mad2), I understand that this is likely unrealistic. I would be supportive of any in vitro approach that directly tested the model the authors propose. Alternatively, the authors should tone-down their claims, and present alternative models that would explain the phenotypes observed for the HTP-1 mutants.

We have performed additional experiments to provide further support for our proposal that the interaction of the β5-αC loop with the HORMA core, particularly with β5 to form the predicted loop-engaged conformation, is an important aspect of HORMAD function. These experiments, detailed below, include both in vivo and in vitro assays to test function of different HTP-1 mutants.

First, we have added in vitro experiments to evaluate how different HTP-1 mutants bind to a closure motif (CM) sequence from HIM-3 by co-expressing HTP-1 and the HIM-3 CM linked to 6His-MBP in bacteria. These experiments demonstrate that the β5-αC loop of HTP-1 is required for closure motif binding and that the ability of

HTP-1 to bind a closure motif is essential for protein stability (Figure 7f). This last point is demonstrated by the reduced levels of HTP-1 protein when WT HTP-1 is co-expressed with a mutant version of HIM-3's CM that can't be bound by HTP-1. Conversely, expression of HTP-1 carrying HIM-3's CM at its C-terminus results in very stable protein despite failure in binding to the external HIM-3 CM linked to MBP. The effect of CM binding on protein stability in vitro is supported by new experiments monitoring HTP-1 protein levels in vivo (whole worm protein extracts followed by western blot analysis). These demonstrate that protein levels of HTP-1 loop deletion mutants are severely reduced compared to WT HTP-1, while levels of HTP-1 carrying HIM-3's CM at its C-terminus are similar to WT HTP-1 levels (figure 7g), despite failing to load on axial elements.

Second, we have created a new HTP-1 mutant, HTP-1^{A139K} (corresponding to HTP-3^{A137K}) that has confirmed that this single amino acid substitution, which is predicted to hinder the interaction between the β5-αC loop and the HORMA core of HTP-1, prevents axis loading in vivo and HIM-3 CM binding in vitro (Figures 7f and 8i). Moreover, this single amino acid substitution reduces in vivo and in vitro protein levels in a manner analogous to mutants carrying loop deletions (Figures 7f and 8k). These findings demonstrate that the role of the role of A137 (A139 in HTP-1), located in the region of the β5-αC loop predicted to form a β strand that pairs with β5 in the loop engaged conformation, in ensuring axis loading is conserved between HTP-1 and HTP-3.

Third, we have also created a new HTP-1 mutant (HTP-$1^{SCR\ 151\text{-}162}$) in which residues 151-162 of the β5-αC loop (same residues that were deleted in the short loop mutant already reported in the first version of our manuscript) were replaced by a scrambled sequence in which each of the 12 amino acids is replaced by a different one. In contrast to the region containing A139, this region of the loop is not predicted to form secondary structure or interact with the HORMA core in the loop engaged conformation. Analysis of HTP-$1^{SCR\ 151\text{-}162}$ mutants demonstrates normal loading to axial elements and normal chiasma formation in vivo, as well as normal binding to HIM-3 CM and protein stability in vitro (Figures 8m-o). All these despite the 12 amino acid substitutions and in clear contrast to the strong in vivo and vitro defects displayed by both HTP-1^{A139K} carrying a single mutation and by HTP-$1^{\Delta 151\text{-}162}$ carrying 12 amino acids deletion. These findings support that residues 151 to 162 constitute a flexible region that acts as a spacer sequence to allow the N-terminal region of the β5-αC containing A139 to interact with residues in the HORMA core, as proposed in the loop engaged conformation.

We have rewritten sections of the discussion to more clearly correlate our findings to previous research in Mad2 showing that a flexible region at the N-terminus competes with the safety belt for binding β5 in the HORMA core in a manner that determines Mad2 conformation. We also clearly state that further biophysical and structural studies will be needed to fully understand how the β5-αC loop interacts with the HORMA core to control the folding and function of mHORMADs.

Minor points

An introductory panel(s) for figure 1 would help the reader better understand the questions the authors are attempting to address

Panel "a" of figure 1 now contains a cartoon of a dissected germline showing examples of the number of chromatin bodies present in diakinesis oocytes of crossover competent (6 bivalents) or crossover deficient (12 univalents) genetic backgrounds.

In Figure 2b the alphaA helix is coloured solid red in the sequence alignment, and partial red in the model. Also a different red tone would be welcome in the model, to match to sequence alignment colouring.

We have modified panels "a" and "b" in Figure 2 to make colours consistent. The red tone used to label the HORMA-A region is now the same in the structure model in panel b, in the cartoon at the bottom of panel "a", and in the box indicating the different HORMA regions in panel "b". We have also enhanced the resolution of the HTP-1 HTP-2 alignment so it can be clearly viewed at higher magnification.

Figure 3d and 3e - Despite observing a larger zone of DSB-2 activity in the htp-1 htp-2HORMAC mutant, fewer RAD-51 foci were observed. This should be clearly explained in the corresponding results section.

We have modified the text to clearly state that numbers of RAD-51 during early prophase are lower in *htp-1*$^{htp\text{-}2\ HORMA\ C}$ mutants than wild-type controls. We also mention that this reduction is likely due to HTP-$1^{htp\text{-}2\ HORMA\ C}$ being only partially competent in supporting the DSB formation role of HTP-1.

Figure 4a - the amino acid substitutions used should be made more clear.
Again, the authors should be consistent with the colouring. In figures 4 and 5 the safety belt is coloured in tan, whereas in figure 7 the safety belt is blue and the closure motifs are orange/tan

We have modified Figure 4a to make the amino acid substitutions clearer by enlarging the font size and moving the position of some of the substitutions.

All panels depicting structural predictions and cartoons of different mHORMAD conformations have been revised to display consistent colours for the safety belt and the β5-αC loop.

Reviewer #4 (Remarks to the Author):

In the manuscript, Barroso et al. investigated meiotic HORMAD proteins of C. elegans (htp-1, htp-2, and htp-3), suggesting a related but a specific bimodal functionality of these proteins. I have focused my review especially on the in silico part of the study. The authors have utilized MD simulations to study one of the states of these meiotic proteins, as well as used ColabFold structure predictions. The manuscript is interesting, but I have some concerns especially related to linking the information obtained from the simulations to the experiments. Also, the simulation data appears somewhat thin to draw the conclusions presented in the manuscript.

1) There seems to be a quite extensive gap between the conducted experiments (in vivo) and the MD simulations presented in the manuscript. The authors should provide at least some biophysical experimental evidence to support the proposed findings from the simulations.

The main conclusion reached from our MD simulations is that the β5-αC loop is a highly flexible region that can move downwards towards the HORMA core. This finding, combined with structural predictions, particularly that of WT HTP-3 suggesting that the β5-αC loop directly interacts with the HORMA core in the same region bound by the safety belt in a closed conformation, led us to investigate how specific residues and protein regions contribute to the proposed loop engaged conformation. Our new data identifies a specific residue (A139) in HTP-1 β5-αC loop, predicted to mediate an interaction with β5, as essential for HTP-1 function in vivo and in vitro (Figures 7f and 8i-k). This residue corresponds to HTP-3 A137 and our original manuscript showed that mutation of this residue (HTP-3^{A137K}) ablated HTP-3 loading to chromosomes, inducing severe meiotic defects. These observations are consistent with an essential role for the conserved A137(HTP-3)/A139(HTP-1) residue in the acquisition a conformation competent in loading to chromosomes. In contrast, we now show that simultaneously mutating 12 amino acids on the C-terminal region of the β5-αC loop, which are not predicted to form stable interaction with the HORMA core in the loop engaged conformation, do not hinder HTP-1 functionality in vivo or in vitro (Figures 8l-o). These new findings provide strong support for the functional relevance of residues and domains that facilitate the β5-αC loop-HORMA core interaction. Although we agree that biophysical experiments would help to further clarify how the position of the flexible β5-αC loop contributes to the proposed HORMAD conformations, we feel that, as highlighted by reviewer 3, these experiments would be out of the scope of the current manuscript. We now clearly state in the discussion that future biophysical and structural studies will help elucidate how the β5-αC loop contributes to different mHORMAD conformations.

2) There is variability among the simulation replicas; for instance, in specific regions in the RMSF plots. Based on this, 3 replicas seem insufficient to describe the system behaviour to be compared to other systems. Following the general recommendations 5-10 replicas for each system is clearly needed and would justify and solidify the findings/suggestions derived from the simulations.

We now have 5 replicas per system and furthermore have extended each replica to 1 microsecond – this more than doubles the original simulation data reported. We show that observations such as distance between β5-αC loop (r144-148) and safety belt (r225-227) being longer in HTP-1 still stand.

3) It seems a bit questionable, if the HTP-1Mut7 simulations are stabilized within the simulation timeframe based on their RMSDs. Thus, it is doubtful if there is enough time for the proper system relaxation with the modified system to describe its biologically relevant configuration / conformational ensembles. Moreover, based on the analysis there is a large discrepancy in the behaviour of one of the replicas compared to the other (heavily related to the point 2). This clearly indicates the need for more replicas and extended simulations. I would also suggest cutting the “equilibrium time” in the beginning (needed for systems to equilibrate) out from the analysis.

The simulations have been doubled in length. The RMSD vs time plots now show clear plateaus in all simulations, indicating that the protein reaches a stable conformation across all systems. This is especially the case in RMSD calculated with the β5-αC loop excluded (Figure S5c), which remains flexible during the simulations. We have included the 5 replicas in all analysis.

4) The authors should stress-test their ColabFold predictions, especially that of htp-3, how stable they are by MD simulations, as they are already studying other systems with this method. This could also provide additional interesting insights.

While this is may be an interesting point to address, we do feel that here HTP-3 simulations are beyond the scope of this study - we have already more than doubled the original simulation data in response to the reviewer’s comments – in which we agreed. But the HTP-3 simulations, we feel would constitute a different study.

5) AlphaFold predictions with the full sequence predict an additional helix located with very high confidence on top of the htp-1 (AF-Q20305-F1) and htp-2 (AF-Q95XC8-F1) that could and would have a major impact on the authors predictions related to the loop movements. This would be highly conflicting the proposed model, and the authors should reflect/justify their results related to these predictions.

We are aware that AlphaFold predicts and additional alpha helix formed by sequences outside the HORMA domain of HTP-1 and HTP-2. However, this predicted helix is not present in other HORMADs, including HTP-3, and given that we now show that the HTP-1 mutation corresponding to HTP-3^{A137K} (HTP-1^{A139K}) mutation, which is predicted to hinder interaction between the β5-αC loop and β5 on the HORMA core, shows the same phenotypes in vivo (impaired loading to axial elements and reduced protein levels), we think it unlikely that helixD is affecting the ability of the β5-αC loop to interact with the HORMA core. In the AlphaFold prediction of full length HTP-1 mentioned by the reviewer (image included below), helix D (green) is located to the right of αC and based on this position we would not expect it to interfere with the ability of the β5-αC loop (blue) to move downwards towards the safety belt (cyan) or β5 (red) on the HORMA core. In addition, as part of a separate study, we have created a mutant lacking helix D, which displays extensive loading to chromosomes, in contrast to the loop mutants analysed here.

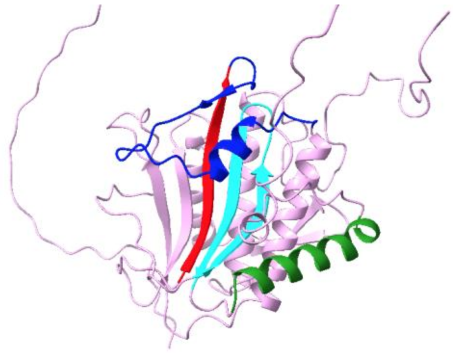

6) The authors note the role of PTMs in the discussion. The authors should clarify the relevance of the studied state without any PTMs, as now all the modelling (MDs and ColabFold) assume only unmodified proteins. What is the evidence that this state is preferred without any PTMs? How accessible the potential PTM sites are for modifications from the proposed configuration?

The potential involvement of PTMs affecting the behaviour of the β5-αC loop is raised as a hypothetical possibility in the discussion. There are no specific PTMs known to occur in the β5-αC loop that could be tested in our model. What we are proposing here is that PTMs may provide an additional layer of regulation to the behaviour of the β5-αC loop.

7) The authors point out the potential important role of histidine residues in the conformational behaviour with selected proteins. However, no information is given how the tautomeric / protonation states of the histidines are modelled into the structures. Is there some bias to the results from this and would the histidine states explain some of the behaviour?

We have added a panel (Figure S6b) indicating that histidine residues are modelled in the neutral tautomeric form, with a proton on the Nε2 atom of the imidazole ring. This reflects the expected protonation state and preferred tautomeric form of histidine in proteins at physiological pH.

We examined the hydrogen bonding interactions of histidine residues to assess if these are influenced by the choice of tautomer. Our analysis revealed that the distances between both protonated (Nε) and non-protonated nitrogen (Nδ) and their hydrogen bond partners are comparable, indicating that the selected tautomer does not introduce bias. We have included the distance plots for H152 over the course of 5 independent 1000 ns simulations below.

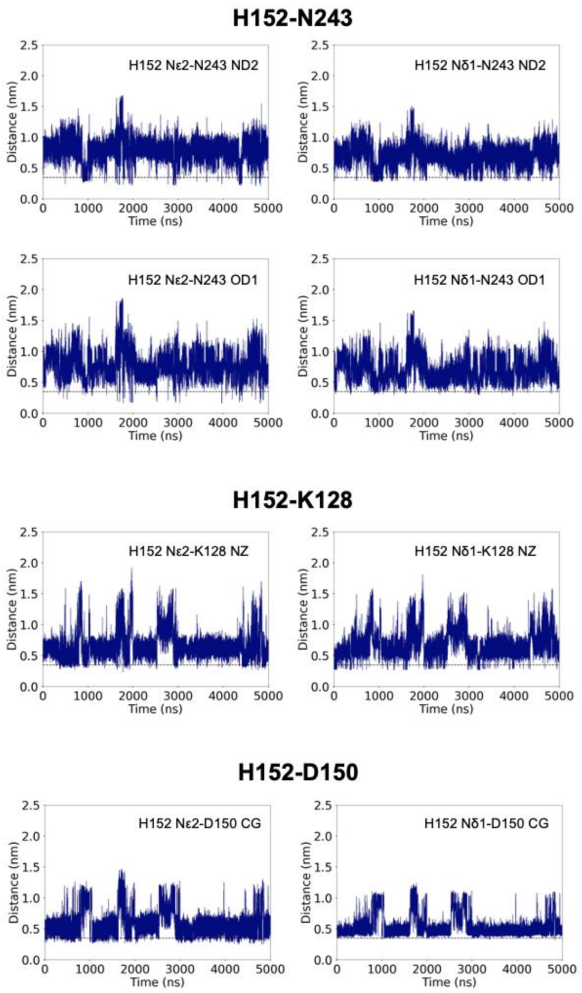

Other points

1) Please provide the missing information related to the size of the simulation box.

This has been added to the methods section.

2) “locally written code” It is totally unclear what is this code and to which analysis it refers to?

We have added more details to the methods section to clarify these points.

3) PCA analysis is insufficiently described. How was the PCA conducted? What are the contributions of PC1s and PC2s for each system? Which software/code was used to generate the Fig 5c images? Please label the most important structural elements also in the Fig 5c to improve its readability.

To better illustrate the full range of β5–αC loop motion (both upward and downward) more clearly, especially in HTP-2 and HTP-1mut7, we have replaced PCA data in Figure 5 with superimposed frames sampled every 100 ns from one representative simulation of each protein system (Figure S6a).

4) The authors do not state how the simulation data is made readily accessible for the readers. Please provide the simulation data available, e.g. into a selected repository.

We have uploaded 10 frames of each trajectory (each 100 ns) and corresponding mdp and itp files to Zenodo and provide the link to access these files in the methods section.

5) Figure 2b, the sequences are not properly aligned, some letters are jumping in the rows. Please improve the figure and its readability.

We have included a higher resolution alignment that has solved this issue.

6) Figure 5e: surface representation is unclear. This should be improved. I suggest displaying this surface with a single colour, and to display the surface of the rest of the protein (light grey) to provide improved presentation for the reader about the solvent accessibility.

We have changed figure 5e (now 5d) following the suggestions from the reviewer.

7) Typos in fig 5 text: “Solvent assessable”; Protein structure prediction methods: “Supposition”; “PyMOL vX”

We have made these corrections.

Reviewer #1 (Remarks to the Author):

The additional experiments and changes to the manuscript address my main concerns. I would like to commend the authors on a much improved manuscript.

Below I note several minor comments and suggestions that could strengthen the manuscript and make it more accessible for a wide readership.

In figure 4a, I would recommend adding a.a. numbers for all the mismatched positions (and potentially for the whole range), given that they are referred to in the text.

We have added amino acid numbers for all mismatched positions in Figure 4a.

I couldn't find evidence or data supporting this statement: "A mutant strain carrying the E191A and E200K substitutions did not show obvious meiotic defects" on page 13. I recommend adding data or rephrasing.

We have added a new supplemental figure (Figure S5) showing quantification of bivalents in diakinesis oocytes of WT controls and *htp-1*$^{E191A\ E200K}$, which demonstrates normal crossover formation, and therefore successful completion of pairing and recombination, in *htp-1*$^{E191A\ E200K}$ mutants.

On page 18, there's a reference for Fig. 7b-e that should be for Fig. 7b-d.

We have made this correction.

Fig. 7g: I realize the trickiness of quantifying western blots. Given this, I recommend adding at least one additional example of this blot to demonstrate that the result is reproducible.

We have added a new western blot (Figure S9b) showing another repeat of the experiment, which demonstrates consistency of the previous example shown in Figure 7g.

Fig. 9: I found the model, and esp. the graphical language used throughout, very helpful. I do however, suggest linking some of the in vivo phenotypes (or suggested outcomes) to the graphics. For example, adding to the figure which of the conformation the authors presume is competent for binding to the axis and which are prone to degradation would be quite helpful. In addition, referring to the labels in the text (e.g., "C-HORMAD", etc.) would improve readability.

We thank the reviewer for their positive comments of the model depicted in Figure 9. We have added to the figure a description of how different conformations relate to axis loading by binding CMs ("CM binding competent" in the case of U-HORMAD, "Bound to axis" for C-HORMAD, and "No axis binding" for SC-HORMAD). We have also added the labels used in the figure (U-HORMAD, C-HORMAD, and SC-HORMAD) to the detailed description of the conformations shown in Figure 9 to the second paragraph of the discussion.

Fig. 5C-D: Any graphical indication of what exactly is quantified in the graphs would be helpful. In C, I *think* that the average of the value in the y-axis is quantified, but I'm not actually sure. This is especially important since, for example, it is the variability for mut7 that is the most striking difference. So I would suggest focusing the reader's attention on the relevant difference.

We have modified the figure legend of Figure 5 to more clearly describe graphs in panels C and D.

Throughout, the authors switch between discussing parts of the proteins based on the numbering system of the alpha helices and beta sheets, and the functional names ('safety belts'). The labeling of the figures is also not always consistent (e.g., Fig. 2b, 4a and 5a). Unifying this across the text and figures will improve the readability of the manuscript.

We have made the labelling of the safety belt (orange) and the β5-αC loop (violet blue) consistent across all figures. Figures in which these two regions are not highlighted, for example when different regions of the HORMA domain of HTP-1 and HTP-2 used for in vivo mutational analysis (Figure 2b) or structures showing IDDT confidence scores (Figures 6e, 8b etc…), use different color schemes that are clearly indicated in the corresponding key and figure legends.

Reviewer #2 (Remarks to the Author):

The authors have addressed my experimental concerns. I particularly appreciated the analysis of the htp-1SCR

151-162 mutant, which was a particularly clever addition to the manuscript.

I have two additional suggestions to help with their Discussion, which nicely summarizes the experimental results but could address some caveats more concretely:

1. Can the authors provide information about which conformations in Figure 9 are stable (expressed at levels comparable with wildtype) or unstable and which are competent and incompetent for axis loading?

We have added to the figure a description of how different conformations relate to axis loading by binding CMs ("CM binding competent" in the case of U-HORMAD, "Bound to axis" for C-HORMAD, and "No axis binding" for SC-HORMAD). We have also added the labels used in the figure (U-HORMAD, C-HORMAD, and SC-HORMAD) to the detailed description of the conformations shown in Figure 9 to the second paragraph of the discussion. We think that adding the different mutations in the β5-αC loop of HTP-1 and HTP-3 found to reduce protein stability will make the model too complicated. Therefore, we prefer to keep the effect of specific mutations on protein stability and axis loading in the discussion.

2. A major point of the paper seems to be that some mutants destabilize the protein, reducing protein levels, but not abrogating them, both in vivo and in vitro, either explaining or contributing to the axis loading defects they observe. The authors argue that since the htp-1 HIM-3CM mutant fails to bind meiotic chromosomes but is expressed at levels comparable to wildtype HTP-1, this demonstrates that loading onto meiotic chromosomes does not affect protein stability per se. While that is definitely one compelling argument, I think another compelling argument that these mutations are functionally relevant is that, critically, these mutants do not produce the same phenotype as the deletion of the htp-1 gene. These data demonstrate that even at reduced protein levels, these mutations produce striking effects on meiotic progression, pairing, synapsis and recombination distinct from complete loss of the protein, arguing against the possibility that the observed phenotypes are because these mutations destabilize the protein.

I'm concerned that this point is not made as plainly as it could, potentially obscuring the important results of the detailed biochemical and mutational analysis. I recommend that the authors make this point more clearly, earlier in the discussion. This will ensure that the rest of the discussion, with its compelling argument about a more expansive set of conformations associated with meiotic HORMADs and the consequences for function, is appreciated as much as it should be.

We thank the reviewer for this suggestion. We have clearly stated in the results sections describing the phenotypes of mutant worms expressing HTP-1$^{\Delta 151\text{-}162}$ and HTP-1$^{\text{HIM-3 CM}}$ that both proteins retain some HTP-1 functionality despite their failure to associate with axial elements, and in the case of HTP-1$^{\Delta 151\text{-}162}$ despite the severe reduction in protein levels (pages 17 and 18). We now also state in the discussion that the in vivo phenotypes of some β5-αC loop mutants are different from those seen in worms lacking HTP-1, demonstrating that these mutations do not fully abrogate HTP-1 function despite their severe effect on overall protein levels.

Reviewer #3 (Remarks to the Author):

The authors have satisfied my concerns, and really gone above and beyond in a very rigorous response. I recommend acceptance of this excellent, fascinating, and important manuscript.

We thank the reviewer for their comments and support.

Reviewer #4 (Remarks to the Author):

I thank the authors for revising the manuscript and addressing my comments. I am satisfied with their responses and modifications. As a side note, it is rather interesting to see how the pIDDTs seem to correlate with RMSFs (Figs S5a&b). I have listed below some minor points and errors that could be fixed to polish the manuscript.

Please use consistent colors for the highlighted structural regions throughout the manuscript. It is difficult to read while this change all the time from figure to figure and panel to panel. (This applies to the 3D-structures and how those regions are highlighted in the plots)

We have revised the use of colors to ensure consistency across structural models and plots in which the β5-αC loop (blue) and the safety belt (orange) regions are highlighted. Figures in which these two regions are not highlighted, for example different regions of the HORMA domain of HTP-1 and HTP-2 used for in vivo mutational analysis (Figure 2b) or structures showing IDDT confidence scores (Figures 6e, 8b etc…), use different color schemes clearly indicated in the corresponding key and figure legends.

Fig 5B) It is unclear which replica is shown in the figure, please specify.

The example shown in Figure 5B corresponds to replica 1. This is now indicated in the corresponding figure legend.

Fig 5C) I would suggest to either show the 3D structure in an orientation as it is in the other panels and/or highlight the β5-αC loop and the safety belt regions as in 5a.

We have implemented these changes. The 3D structure is now in the same orientation and highlighting the same regions as those in Figure 5a.

Fig. S5a) Wrong colors in the figure text: "indicating the β5-αC loop in orange and the safety belt region in blue.". Please highlight the β5-αC loop and the safety belt region also in the pIDDT plots.

We have corrected the figure legend and, as requested, we have highlighted the β5-αC loop and the safety belt region in the pIDDT plots shown on panel a. Please note that this figure is now Figure S6, as we have added an additional supplemental figure (Figure S5) to address a comment from reviewer 1.

Fig S5b) Please use consistent colors for the highlighted regions here with the presented 3D-structures to improve clarity and readability.

We have changed the colors of the highlighted regions to ensure consistency with colors used in other panels (violet blue for the β5-αC loop and orange for the safety belt).

Fig S5d) Why the color of HTP-1mut7 position 1 in the left panel is not orange as indicated in the figure text?

We have modified the figure legend to explain that the two shades of orange depict the two different positions of the β5-αC loop in the HTP-1^{mut7} protein.

Figure S6b) This figure is not necessary and can be omitted (as you provide the information in methods), and it allows you to fit the S6 in one page.

We have removed panel b from Figure S6 as requested by the reviewer.

The authors write in p.33 "…the preferred tautomeric form of histidine in proteins at physiological pH" – I would argue that this is highly dependent on the environment and the preference is higher the more solvent exposed histidine is (also in the cited paper it is pretty evident that not all His will be in this form). But feel free to keep the text as it is if you wish.

We have edited this sentence to indicate that it refers to the Nτ nitrogen farthest from the side chain α-carbon.

Please add the version numbers for VMD and PyMOL and properly cite PyMOL.
(e.g. The PyMOL Molecular Graphics System, Version 3.0 Schrödinger, LLC.)

We have indicated the version numbers for VMD and PyMOL and cited PyMOL as requested.

There are also still two typos of "PyMOL vX" in the methods.

We have corrected this by indicating that PyMOL v2.5.0 was used.

This is an important, rigorous, exceedingly well-executed manuscript that identifies key differences between meiotic HORMADs and other HORMAD proteins to potentially explain the unique behavior of meiotic HORMADs during meiotic prophase. Exploiting the fact that C. elegans have a duplicated, non-essential version of HTP-1, HTP-2, the authors perform a series of creative swapping experiments to test what regions of HTP-1 and HTP-2 explain the meiotic requirement for HTP-1. Using a combination of structural analysis and in vivo testing, the authors change key residues in the safety belt region of HTP-1 to residues found in HTP-2 to demonstrate that safety belt region explains the functional differences between HTP-1 and HTP-2. Next, the authors used equilibrated molecular dynamics (MD) simulations to determine what effect these mutations have on the structure and mobility of portions of HTP-1/HTP-2. These analyses identified another structurally mobile region, the β5-αC loop, that is longer and more structured in meiotic HORMADs than in Mad2 and appears to play a role in stabilizing the closed conformation through an interaction with the safety belt. The authors test the functionality of this loop in HTP-1 and find that deletion of this loop either prevents or destabilizes chromosomal loading, affecting synapsis, crossover formation and feedback mechanisms. Moreover, the function of this loop appears to be functionally conserved in another essential meiotic HORMAD, HTP-3. Since removal of this loop in the budding yeast meiotic HORMAD, Hop1, produces a conformation where the HORMA domain adopts a closed conformation without binding a closure motif, the authors conclude that the defects they observe in these "loopless" mutants are a product of adopting the closed conformation without binding their partners, preventing their chromosomal loading. They test this possibility by installing a closure motif that HTP-1 binds with higher affinity (HIM-3CM) than its own and mimicking the htp-1 "loopless" mutation. They then test the possibility that this loop may further stabilize the closed conformation that meiotic HORMADs adopt when they bind closure motifs on their chromosomal partners by mutating residues identified in their simulations as important for this stabilization. These mutants produce protein that are less stably bound to chromosomes and exhibit defects in synapsis and the activation of feedback mechanisms. Finally, additional simulations with HTP-3 suggested that a version of HTP-3 that is not bound to a protein with a closure motif may also engage this loop, so that it contacts the open HORMA domain. They call this the "loop engaged" conformation and test the functionality of this proposed structure by making a mutation that is predicted to disrupt this interaction and find that HTP-3 fails to load and support synapsis and recombination. Simulations of other meiotic HORMADs in which the safety belt has been deleted show that they also adopt this "loop engaged" conformation, suggesting its general relevance.

The results are largely convincing, clear, appropriately interpreted and mostly communicated with clarity and accessibility. I have a few suggestions to improve the clarity and justify some of the interpretations of the manuscript.

Major

A figure showing the differences between the β5-αC loop in meiotic HORMADs and Mad2 and Rev7 would be useful, given the focus on this region in the study.

Why were the simulations in Figure 4 performed without the closure motifs? How do the authors imagine full length HTP-1 and HTP-2 structures maintain their closed conformations without the closure motifs at this stage of their analysis? This becomes more clear later in the paper but is distracting and unclear in this section.

What are the observed number of DAPI stained bodies in *htp-1*^{D144KL156K} mutants, given their low level of embryonic lethality?

In general, the manuscript would benefit from more clarity and rationale for experiments and interpretations, especially with the CollabFold simulations. For example, the fact that *htp-3*^{A137K} mutant phenotypically resembles the loopless *htp-3* mutant (*htp-3*$^{\Delta 136\text{-}149}$) could be explicitly highlighted to support the interpretation that the unliganded, closed engaged loop conformation has in vivo relevance before the discussion.

I was confused by the distinction between the closed capped conformation and the loop engaged conformation and concerned that the introduced mutations could be destabilizing both, particularly since all of these mutations are in the loop. Given the novelty of this striking finding, additional experiments would convince me of the strength of this claim. For example, do mutations in htp-1 that mimic *htp-3*^{A137K} look different than *htp-1*^{D144KL156K} and do mutations in htp-3 that mimic *htp-1*^{D144KL156K} look different than *htp-3*^{A137K}?

The stability and expression levels of mHORMAD mutants (*htp-1* $^{\Delta 140\text{-}162}$, *htp-1* $^{\Delta 151\text{-}162}$, *htp-3* $^{\Delta 136\text{-}149}$, *htp-1*^{D144KL156K}, *htp-1*$^{HIM\text{-}3CM,}$ and *htp-3*^{A137K}) that fail to load or exhibit reduced loading on meiotic chromosomes should be verified by Western.

Minor:
Reference 32 should be updated to the peer reviewed paper published in Nature Communications
